# Potential of different area-based governance mechanisms for achieving Global Biodiversity Framework goals

**DOI:** 10.1038/s41467-026-76225-9

**Published:** 2026-08-03

**Authors:** Pablo J. Negret, Victor J. Rincon-Parra, Kendall R. Jones, Vanessa R. Rathbone, Sidney Novoa, Marvin Quispe, Armando Valdés-Velásquez, German Forero-Medina, Judith Schleicher, Tatsuya Amano, Miguel Saravia, Julie Gwendolin Zaehringer

**Affiliations:** 1 Wyss Academy for Nature, Bern, Switzerland; 2 Centre for Development and Environment, University of Bern, Bern, Switzerland; 3 Institute of Geography, University of Bern, Bern, Switzerland; 4 Hawkesbury Institute for the Environment, University of Western Sydney, Richmond, Australia; 5 Département de biologie, Université de Sherbrooke, Sherbrooke, Canada; 6 Wildlife Conservation Society, Bronx, NY 10460 USA; 7 School of Agriculture, Food and Ecosystem Science, The University of Melbourne, VIC 3010 Melbourne, Australia; 8 Asociación para la Conservación de la Cuenca Amazónica, Lima, Perú; 9 Grupo de Investigación en Sistemas Socio-Ecológicos, Universidad Peruana Cayetano Heredia, Lima, Perú; 10 Wildlife Conservation Society, Cali, Colombia; 11 Deutsche Gesellschaft für Internationale Zusammenarbeit (GIZ) GmbH, Eschborn, Germany; 12 School of The Environment, The University of Queensland, Brisbane, QLD Australia; 13 Centre for Biodiversity and Conservation Science, The University of Queensland, Brisbane, QLD Australia

**Keywords:** Environmental impact, Climate-change mitigation, Conservation biology, Governance

## Abstract

The Kunming–Montreal Global Biodiversity Framework (GBF) includes a target to protect 30% of land by 2030, identifying other effective area-based conservation measures (OECMs) as complementary to Protected Areas (PAs). However, their contribution to conservation outcomes remains uncertain. Here we show the level of human pressure across the global OECM network and assess the impact of different governance mechanisms on deforestation and associated carbon emissions in the Peruvian Amazon from 2005 to 2021. We evaluate Indigenous Lands (ILs), Non-Timber Forest Product Concessions (NTCs), Logging Concessions (LCs), Mining Concessions (MCs), and PAs using statistical matching. Globally, OECMs show higher human pressure than strictly protected PAs, but similar levels to less strictly protected PAs. In the Peruvian Amazon, PAs are most effective at reducing deforestation (49–53% avoided), followed by NTCs and ILs (33% and 20%, respectively). In contrast, LCs and MCs are associated with increased deforestation (13% and 24%). These findings highlight the complementary role of OECMs and the effectiveness of different governance mechanisms in reducing deforestation and carbon emissions, informing efforts to achieve global biodiversity targets.

## Abstract (web summary)

This study shows that Indigenous Lands and sustainable-use concessions can effectively reduce deforestation alongside protected areas, while extractive concessions increase forest loss, highlighting the complementary role of alternative governance mechanisms in achieving global biodiversity targets.

## Introduction

Tropical forests are among the world’s most biodiverse ecosystems^1,2^. These forest also provide crucial services to humanity^3,4^, including storing and sequestering large amounts of atmospheric carbon, which is key to directly regulating climate^5^. Despite this, the loss and degradation of tropical forests has increased over time^6,7^, contributing up to 20% of the world’s greenhouse gas emissions^5,8^.

Protected areas (PAs) are a critical tool for the conservation of forests and the ecosystem services they provide. There is strong evidence that PAs reduce deforestation and forest degradation pantropically^9^, and more than 18% of global land is now under some kind of protection regime^10,11^. Despite their expanding coverage and success, PAs also face many challenges. One third of PAs globally are under intense human pressure^12^, more than half of protected forests have a substantial degree of anthropogenic disturbance^13^, and 3% of forest within PAs was lost in the first decade of the 20th century^14^. The establishment of PAs has also sometimes been associated with negative social and economic outcomes, including displacement of local communities and restriction of access to local resources^15–17^. Moreover, in some circumstances, the designation of a PA may not be appropriate or feasible^18^, and other area-based conservation measures, such as OECMs, Indigenous and Community Conserved Areas, and other locally governed initiatives, may provide effective and equitable conservation outcomes^19–21^. Collectively, these represent a subset of the diverse governance mechanisms available to support conservation objectives^22^. Here we define governance mechanisms as the system of laws, norms, rules, policies and practices that determine how landscapes and resources are managed and regulated in a spatially defined area^23^.

**Fig. 1:**
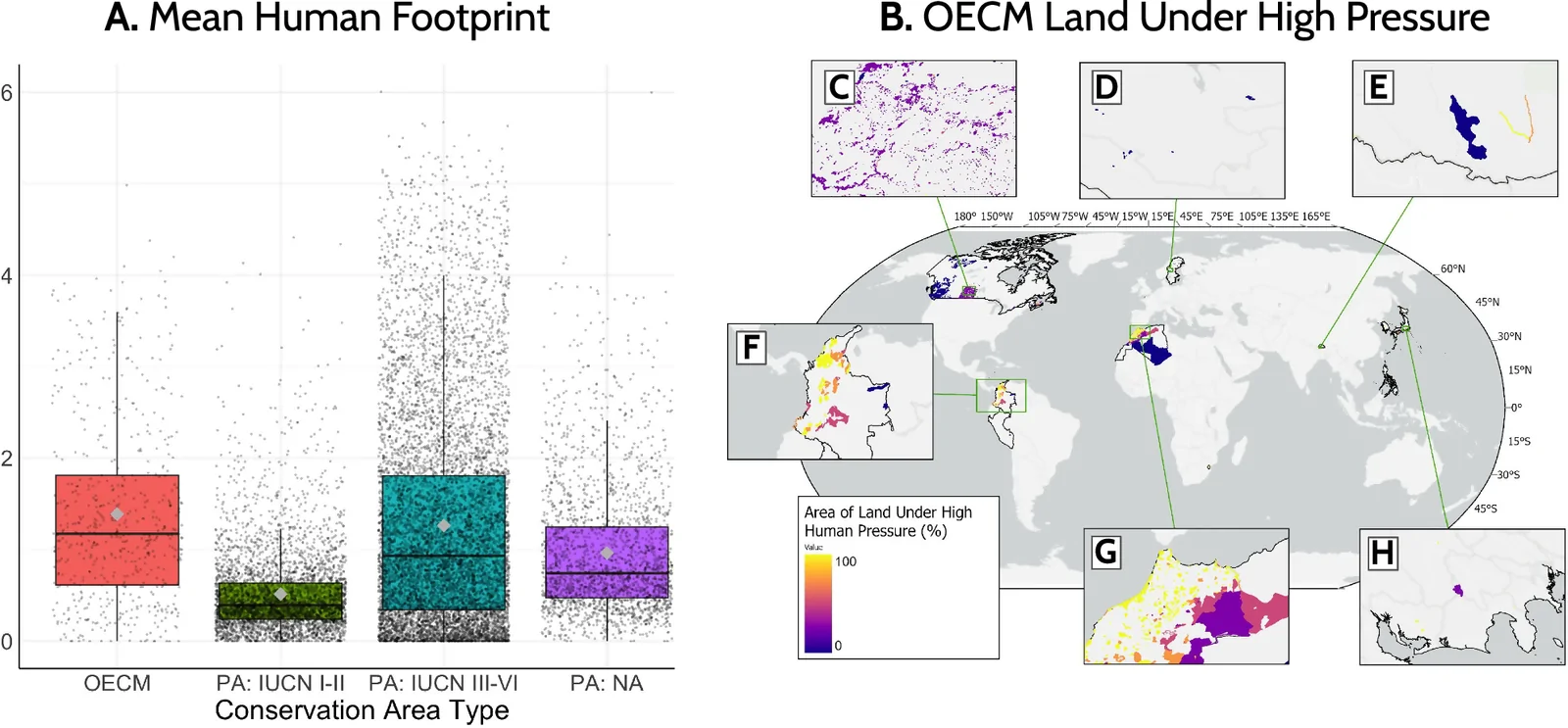
Human pressure across OECMs and Protected Areas. **A** Mean human footprint (HFP) values across OECMs and PAs, for countries that have submitted OECM data to WDPA. Black dots represent mean HFP within individual OECMS and PAs, with coloured boxplots showing grouped statistics. The grey diamond represents mean HFP values within sites in each group. PA: NA refers to PAs that do not report on their IUCN status within the WDPA. This plot represents mean HFP values within each individual OECM and does not consider the area of land within each site. **B** Proportion of land under high pressure (>6.69 HFP) within OECM boundaries. Note – there are OECMs contained in all highlighted countries, but some are very small and are unable to be visualised at the global scale. Inset maps show the proportion of land under high pressure (>6.69 HFP) within OECM boundaries for parts of **C** Canada, **D** Sweden, **E** Bhutan, **F** Colombia, **G** Morocco and Algeria, **H** Japan. Base map derived from Natural Earth data (public domain).

It is now clear that PAs alone are unlikely to be sufficient for meeting the targets of the Kunming–Montreal Global Biodiversity Framework (GBF)^24^, particularly target 3, which calls for the effective conservation and management of 30% of the planet by 2030^24^. In response, much attention is now being placed on other effective area-based conservation measures (OECMs) that can deliver positive conservation outcomes to complement those provided by PAs^16,22^. For a site to be listed as an OECM, it cannot be already classified as a PA, and must be a geographically defined area which is governed and managed in ways that achieve positive and sustained long-term outcomes for the in-situ conservation of biodiversity^25–27^. More than 1.9 million km² of the Earth’s surface has already been reported to the World Database on OECMs^11^, representing approximately 1% of total terrestrial area (Fig. 1). Two thirds of this area is governed by federal or national agencies^28^.

Beyond the formal designation of area-based governance mechanisms, an important challenge lies in understanding how effectively sites under different governance mechanisms deliver conservation outcomes over time. Regardless of the type of area-based governance mechanism, conservation outcomes may erode through processes of conservation abandonment, including both the abdication of management responsibilities (e.g., “paper parks”) and the reversal or weakening of formal protections, such as downgrading, downsizing, and degazettement^29–31^. Conservation abandonment should therefore be explicitly considered when planning and evaluating conservation actions^32^. These dynamics highlight the need to assess not only the extent but also the relative effectiveness of different governance mechanisms, particularly in the context of emerging frameworks such as OECMs, which emphasize sustained and demonstrable biodiversity outcomes^20,21,28^. In this context, understanding how sites under different governance mechanisms contribute to conservation outcomes across different settings becomes particularly important.

Sites recognized as OECMs can be governed through diverse mechanisms, but many governance arrangements that are not formally designated as PAs or OECMs may also deliver meaningful conservation outcomes^21,28^. For example, land management by Indigenous Peoples, including Indigenous Lands (ILs), has been shown, under suitable conditions, to be highly effective in limiting deforestation^9,33–35^. A study in the Brazilian Amazon found that deforestation and burned areas inside ILs were two and four times lower than outside, respectively^36^.

Forest concessions, in which a government temporarily provides public forested land access, management and exclusion rights to non-government actors, represent another governance mechanism that can provide positive outcomes for biodiversity conservation while generating income for local communities^37^. There are at least 122 million hectares of forest concessions in West and Central Africa, Latin America and Southeast Asia^37^, with different uses ranging from industrial extraction, including timber extraction or logging (logging concessions; LCs)^38–40^, to non-timber uses such as agroforestry^41^, tourism and conservation^42^, hereafter referred to as Non-Timber Forest Product Concessions (NTCs). The effectiveness of forest concessions in reducing deforestation varies depending on concession type and political context, but there is strong evidence of positive conservation outcomes under adequate management and supportive political circumstances^38,42,43^. Other governance mechanisms associated with positive conservation outcomes include sacred sites, military areas, private, urban and surfing reserves, and university research areas^21,44^. Whether such sites are formally recognized as PAs, reported as OECMs, or remain under other designations, their conservation contribution ultimately depends on their ecological outcomes and governance performance. In contrast, mining concessions (MC) are primarily established for extractive purposes and are generally associated with increased deforestation and habitat degradation^45,46^. Such activities can lead to substantial forest loss and fragmentation, with consequent negative effects on biodiversity and ecosystem integrity^47^. These differences underscore that governance mechanisms vary considerably in their conservation outcomes, depending on their objectives and management context.

Despite their growing importance, rigorous evaluations of the effectiveness of sites under alternative governance mechanisms with the potential to be designated as OECMs in preventing forest loss, particularly relative to PAs, remain relatively sparse^48–51^. Many assessments fail to account for the non-random location of PAs and OECMs. Some studies, for example, compare areas that are protected to unprotected land in the study area^14,52^, or to neighbouring buffers^53^. This can cause bias in their estimated impact of PAs as they tend to be located in places that are less likely to be cleared^18,54,55^. As countries continue to designate PAs and report OECMs in pursuit of the goals and targets in the UN Framework Convention on Climate Change^56^ and the GBF, it is crucial to understand their contributions and complementarity to achieve the best conservation and climatic outcomes^28,49^.

**Fig. 2:**
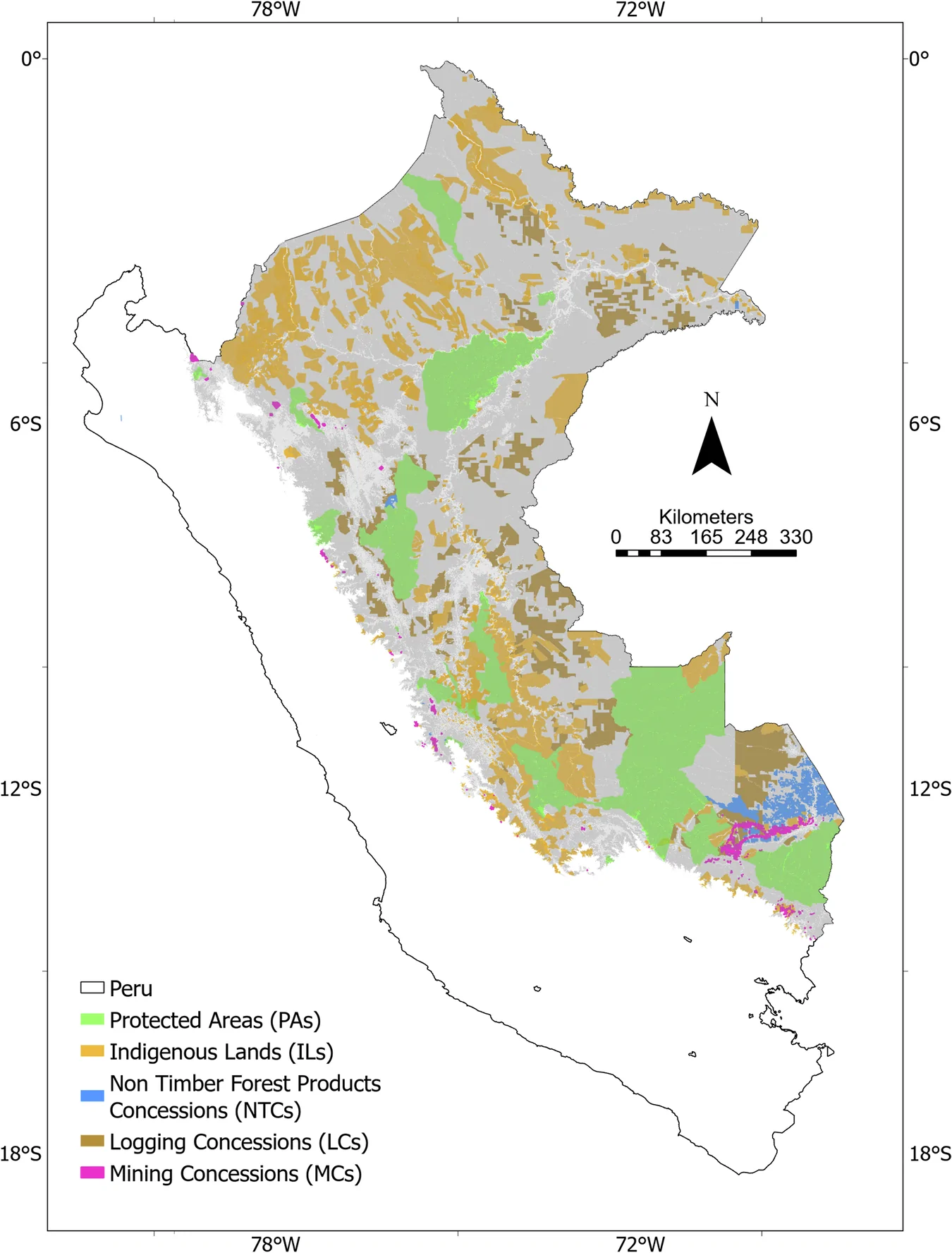
Map of the Peruvian Amazon showing the extent of protected areas, indigenous lands, non-timber forest product concessions, logging concessions, and mining concessions included in the analysis. Darker shades indicate areas that were forested in 2005, while lighter shades represent areas without forest in 2005. Base map derived from Natural Earth data (public domain).

Here, we generate robust empirical evidence on the effectiveness of diverse governance mechanisms in reducing forest loss and associated carbon emissions, thereby informing which mechanisms are best positioned to contribute to biodiversity and climate goals. At the global scale, we assess the extent and intensity of human pressure within sites already listed as OECMs compared to PAs. Focusing on the Peruvian Amazon, we conduct comparative impact evaluations across multiple governance mechanisms using statistical matching to account for their non-random spatial distribution and underlying deforestation risk. Specifically, we quantify and compare the impacts of PAs, ILs, NTCs, LCs, and MCs on forest loss and associated carbon emissions between 2005 and 2021. We use the Peruvian Amazon as our study area (Fig. 2) because Peru as a country is megadiverse^57^ and has multiple conservation governance mechanisms^50^. Additionally, the Peruvian Amazon, which accounts for 60% of the country’s land mass and 90% of its forest^58,59^, is experiencing high rates of forest loss and degradation^50,52,60^. We use statistical matching as this methodology allows to evaluate the effectiveness of conservation interventions by accounting for the non-random spatial distribution of those interventions and of deforestation risk^9,61–63^. We assess the impact of PAs and two governance mechanisms with potential to be designated as OECMs (i.e., ILs and NTCs) on forest loss and its associated carbon emissions in the Peruvian Amazon from 2005 to 2021. We also assess two other governance mechanisms with mostly commercial extractive uses, LCs and MCs, to determine how different governance mechanisms impact forest loss. MCs in particular have been strongly associated with forest^50,64,65^ and biodiversity loss^47^ in the tropics.

## Results

### Human pressure in OECMs and PAs globally

The average HFP score within PAs and OECMS is 1.00 and 1.39, respectively (Fig. 1A), compared to a global average of 6.06^66^. PAs in IUCN category I–II had lower average HFP scores (0.52) than those in category III–VI (1.27), or those without a reported category (0.97; Fig. 1A). Within individual OECMs, average HFP levels vary widely, ranging from 0 (no observed human pressure) to above 6 (high pressure; Fig. 1). Larger OECMs generally have lower human pressure levels, with a small number of very large sites with low human pressure (Supplementary Fig. 33). OECMs have higher mean human pressure than PAs across all IUCN categories, with significant differences found between all categories (Supplementary Table 9), and larger differences seen between OECMs and PAs in IUCN category I–II (Fig. 1A). When considering the total land area within PAs and OECMs under varying levels of human pressure, we find that PAs in IUCN categories I–II have 13% less land under high human pressure levels than OECMs, but OECMs have substantially more land under low human pressure compared to PAs under IUCN categories III–VI (Supplementary Table 10 and Fig. 1B). This is primarily driven by a small number of very large OECMs with a large proportion of land under low human pressure (Supplementary Fig. 33). While both PAs and OECMs have lower mean human pressure levels than the global average, many still face substantial levels of human influence. For OECMs, 69.4% of individual sites have more than half of their area under high human pressure levels (>6.69 HFP score; Fig. 1B), and in PAs, this amount decreases to 43.1% (Supplementary Fig. 34).

### Deforestation and carbon emissions in the Peruvian Amazon

**Fig. 3:**
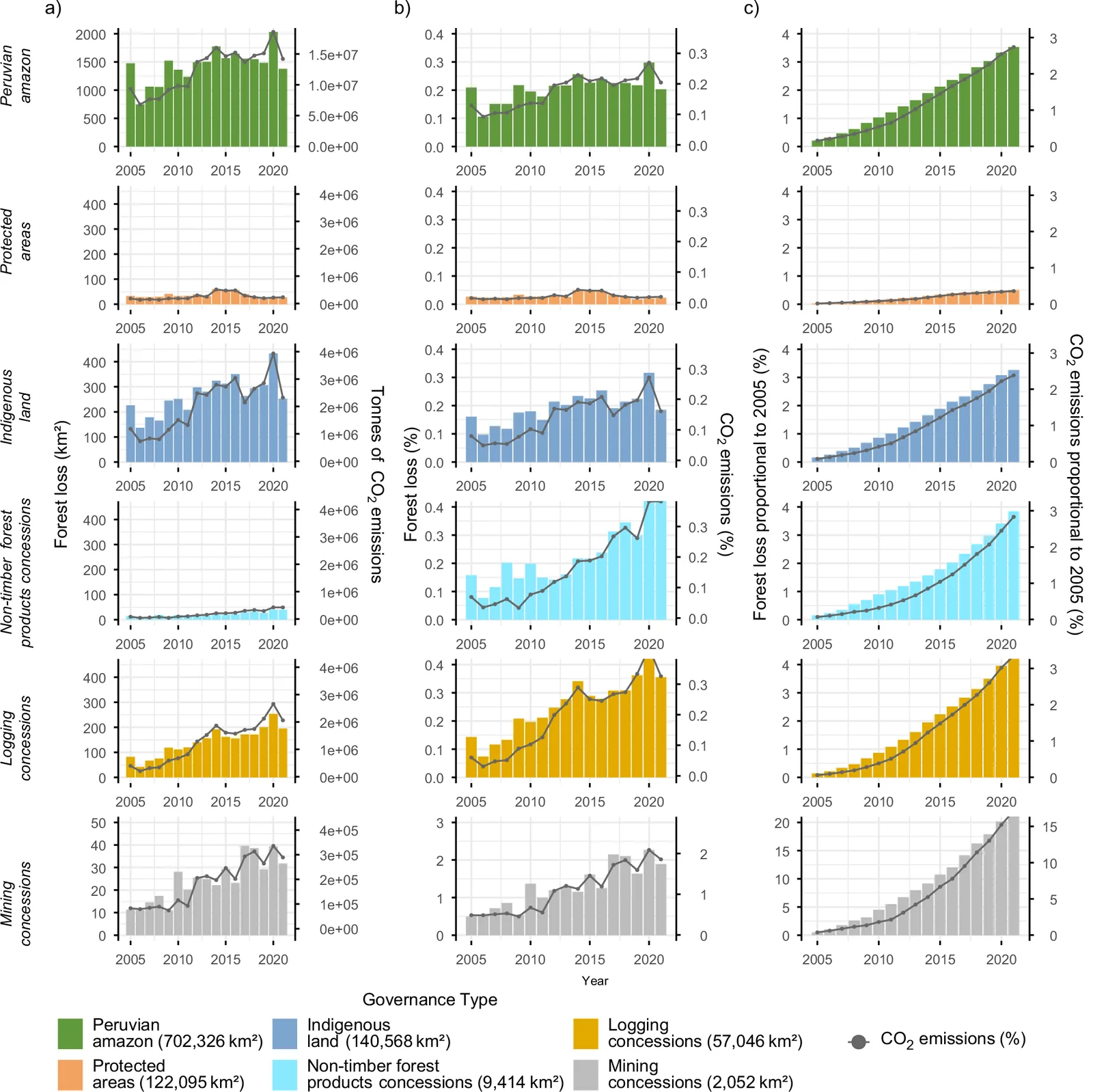
Forest loss and associated carbon emissions across governance mechanisms in the Peruvian Amazon (2005–2021). Yearly absolute area (**a**), percentage (**b**) and cumulative percentage (**c**) of forest lost and carbon emissions for the Peruvian Amazon and inside Protected Areas, Indigenous Land, Non-Timber Forest Products, Logging and Mining concessions in the study area between 2005 and 2021. In parenthesis is shown the total area of forest in 2005 in km² for each class. The left vertical axis shows absolute (km²) and proportional (%) forest loss. The right vertical axis shows absolute (tons) and proportional (%) carbon emissions. Vertical bars show forest loss and gray lines carbon emissions. All the vertical axes are standardized with the exception of the Peruvian Amazon and mining concessions.

Between 2005 and 2021, the Peruvian Amazon lost an average of 1435 km² of forest per year, representing a total net loss of 3.5% of forest since 2005. Annual deforestation fluctuated considerably by year but has generally increased since 2005 (Fig. 3a–c). Across governance mechanisms, ILs had the highest absolute yearly forest loss, followed by LCs (Fig. 3a). In proportional terms, MCs showed the highest yearly and accumulated loss since 2005. LCs experienced the second-highest cumulative loss. On the other hand, PAs showed the lowest yearly and cumulative proportional loss of forest between 2005 and 2021 (Fig. 3b, c).

Carbon emissions in the Peruvian Amazon have consistently increased in absolute and proportional terms since 2005 (Fig. 2a–c). This aligns with the increase in deforestation observed between 2005 and 2021 (Fig. 2a–c). The governance mechanisms with the highest yearly net carbon emissions were ILs, followed by LCs (Fig. 2a). MCs had the highest proportional carbon emissions yearly and cumulative to the carbon stock in 2005, while PAs had the lowest (Fig. 3b, c).

### Impact of the different governance mechanisms on forest and carbon emissions

**Fig. 4:**
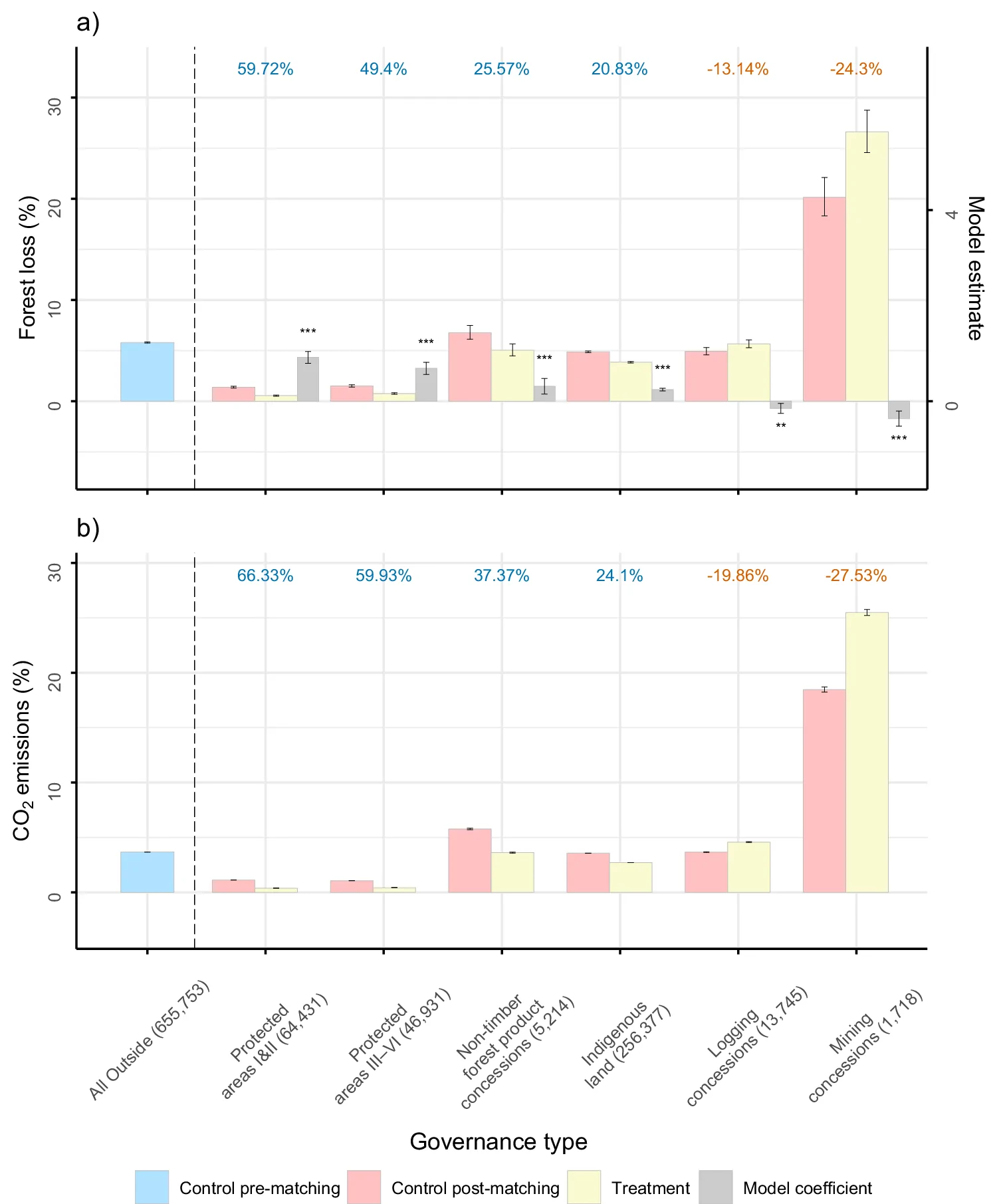
Impacts of different governance mechanisms on forest loss and carbon emissions. **a** Estimated proportional forest loss inside Protected Areas, Indigenous Lands, Non-Timber Forest Product Concessions, Logging Concessions, and Mining Concessions in the Peruvian Amazon from 2005 to 2021, compared against matched control areas. Black lines indicate 95% confidence intervals centred on the estimated proportions (Wilson score intervals for binomial proportions). Grey bars represent model coefficients from logistic regression analyses, with error bars indicating 95% confidence intervals of the estimated coefficients; statistical significance corresponds to the *p*-value of the treatment coefficient (Protected Areas I–II: *p* = 2.06 × 10⁻⁴⁸; Protected Areas III–VI: *p* = 3.68 × 10^−26^; Indigenous Lands: *p* = 1.53 × 10^−70^; Non-Timber Forest Product Concessions: *p* = 1.86 × 10^−4^; Logging Concessions: *p* = 0.00584; Mining Concessions: *p* = 8.07 × 10^−6^). For each governance type, an independent binomial logistic regression model was fitted comparing treated pixels and matched controls; Reported *p*-values are two-sided tests of the treatment coefficient, and no adjustments for multiple comparisons were applied. **b** Estimated proportional carbon emissions for the same governance mechanisms compared against matched controls. Blue and red percentage values above each governance mechanism indicate the relative percentage decrease (blue) or increase (red) in forest loss or carbon emissions within treatment areas compared to matched controls. Values are derived from aggregated 30 m pixel-level binary outcomes (deforested/not deforested between 2005 and 2021) following statistical matching and represent the proportion of pixels deforested within treatment and matched control groups for each governance mechanism. Uncertainty is shown by confidence intervals (estimated analogously for carbon loss). Numbers in parentheses indicate the sample size corresponding to the number of pixels included in the treatment and matched control groups for each governance mechanism.

Our results show that PAs, NTCs and ILs decrease forest loss compared to the matched control areas. PAs were the most effective in preventing forest loss with those in categories I and II having a higher effectiveness than those in categories III and VI (avoiding 60 and 49% of expected loss, respectively). NTCs and ILs also reduced deforestation by 26 and 21%, respectively. On the other hand, LCs and MCs showed a 13 and 24% increase in expected forest loss compared to matched control areas (Fig. 4a). The effect these governance mechanisms had on forest loss patterns was statistically significant (Fig. 4a and Supplementary Table 11). In relation to the carbon emissions represented by forest loss, PAs, ILs and NTCs had lower emissions than matched control areas. This decrease was 66% for PAs in categories I and II, 60% for PAs in categories III to VI, 37% for NTCs and 24% for ILs. On the other hand, LCs and MCs had a 20% and a 28% increase in carbon emissions compared to the matched control areas (Fig. 4b).

### Impact of different governance mechanisms on forest loss by site

**Fig. 5:**
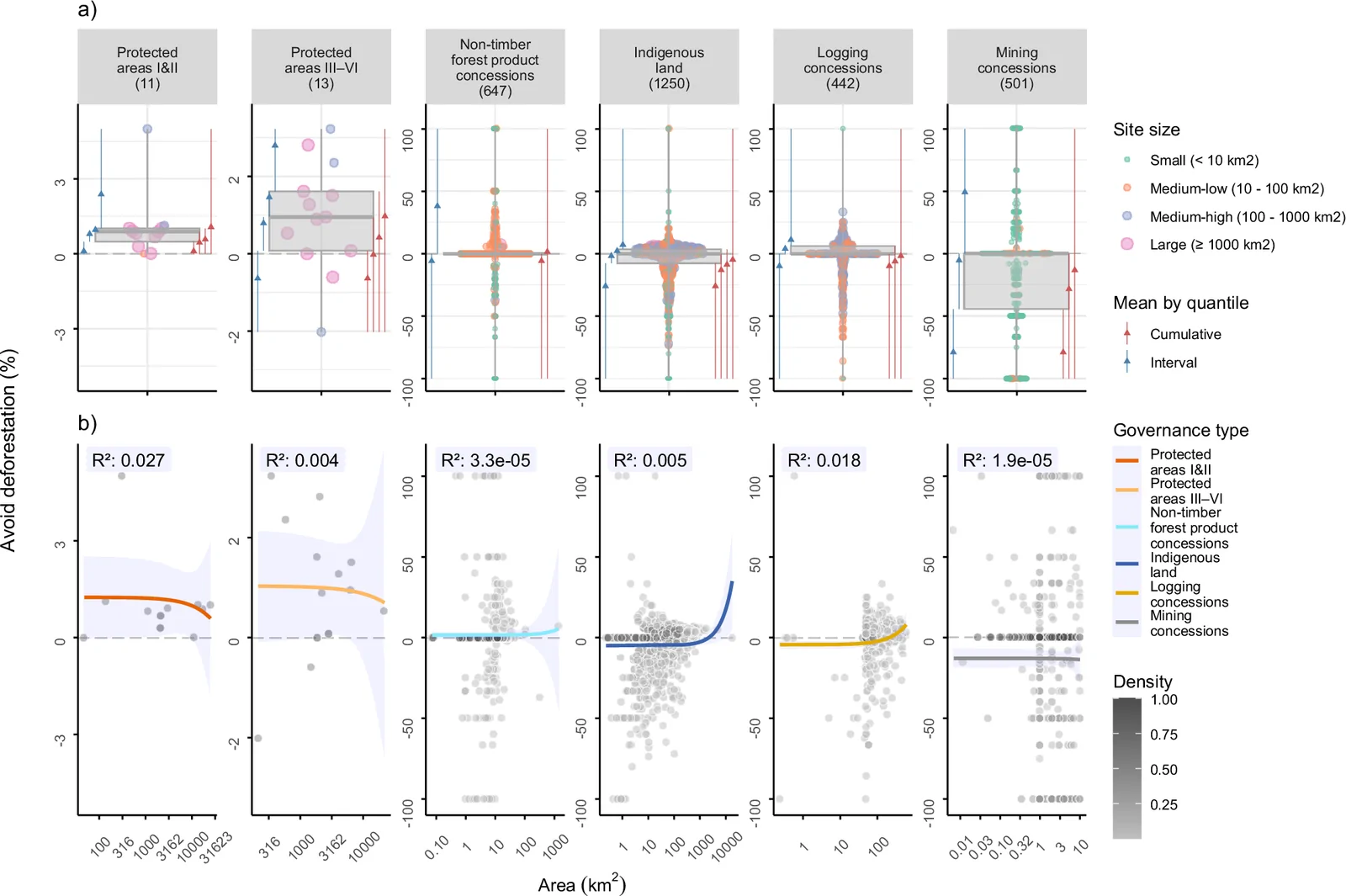
Site-level variation in avoided deforestation across governance mechanisms and site sizes. **a** Estimated avoided deforestation by individual sites under Protected Areas, Indigenous land, Non-Timber Forest Products, Logging and Mining Concessions governance in the Peruvian Amazon from 2005 to 2021. Each site is represented with a point. Point size reflect one of four size categories. The data distribution per governance type is shown in the boxplots, where central lines represent medians and lower and upper lines correspond the second and third quantiles, whiskers reflect the first and fourth quantiles. Blue triangles show the mean by quantiles and red triangles show the accumulated means from the first to the fourth quantile. Vertical lines associated with the triangles indicate the band interquartile range. **b** Scatterplot showing the area of the sites against the avoided deforestation by site. Darker colours reflect higher density of points. The line depicts the tendency predicted by a linear regression model of avoided deforestation against area and the shaded band represents the 95% confidence interval of the predicted mean relationship. The *R*² values reported in each panel indicate the proportion of variance in avoided deforestation explained by the model for each governance type. Numbers in parenthesis in the panel titles indicate the number of sites per governance mechanism. Sample sizes (*n*) correspond to the number of 30 m pixels (deforested/not deforested between 2005 and 2021) included in the treatment and matched control groups for each governance mechanism: Protected Areas I & II (*n* = 6,443,199), Protected Areas III to VI (*n* = 46,931), Indigenous Lands (*n* = 256,377), Non-Timber Forest Product Concessions (*n* = 5214), Logging Concessions (*n* = 13,745), and Mining Concessions (*n* = 1718).

When the results were aggregated at the site level, we found trends broadly consistent with the pixel-level analysis, with the exception of Indigenous Lands (ILs), which showed higher average forest loss per site in treatment areas than in controls (Supplementary Table 12, Fig. 5, and Supplementary Fig. 35). This divergence reflects how observations are weighted in pixel versus site level summaries. Pixel-level analyses up-weight larger sites because they contain more pixels and therefore capture the overall governance mechanism impact on landscape-level forest loss trends, whereas site-level analyses weight all sites equally and better reflect variation in effectiveness at the scale at which management decisions are implemented. As a result, site-level averages are more strongly influenced by the performance of numerous small sites, while pixel-level results are driven by fewer large sites that contain a greater share of forest area. For ILs, there is a higher number of smaller sites than larger ones (Supplementary Fig. 36) and those sites represent a higher proportion of the total pixels used in the analysis (Supplementary Fig. 37). These smaller sites tend to be ineffective preventing deforestation, so the mean avoided deforestation by site is strongly influenced by those small ineffective sites, despite larger sites tending to be effective in reducing deforestation (Fig. 5). At a site level, individual PAs mostly avoided deforestation in relation to the control pixels but the total amount of deforestation avoided by PA was in average low. For the other governance mechanisms, there was a high variation in the avoided deforestation by sites with MCs having the highest dispersion, followed by NTCs (Fig. 5a). For LCs, smaller sites tended to be ineffective at preventing deforestation, and some larger sites were effective. In contrast, MCs in general increased forest loss compared to matched control areas (Fig. 5b).

## Discussion

At the global scale, our results show that while OECMs and PAs face considerable human pressure levels, they still have far lower human pressure than unprotected land. Previous work has shown that PAs face widespread levels of intense human pressure^12^, but there is also evidence that they can reduce deforestation and habitat loss^9^, and contain higher biodiversity than unprotected areas^67^. Here, we showed that, on average, OECMs have similar human pressure levels to PAs, particularly those in IUCN categories III–VI. Less degraded ecosystems, such as those within many PAs and OECMs, support an exceptional confluence of globally significant environmental values relative to the ones that have experienced damaging human actions^68^, so both OECMs and PAs have strong potential to deliver positive conservation outcomes. However, delivering on this potential will require concerted actions to overcome the many challenges facing area-based conservation efforts, such as underfinancing and understaffing^69,70^, conservation abandonment^29,31,32,71^, and resource extraction^72^. In addition, recent work shows that 42% of reported OECMs were previously classified as PAs^31^. These changes reflect shifts in how sites are reported and categorized within international conservation frameworks, rather than necessarily changes in their legal protection or management. A clearer understanding of the implications of such reporting changes for conservation accountability and outcome assessment is therefore needed. Importantly, our global analysis does not assess the legal or governance changes associated with site reclassification, but instead evaluates human pressure within areas currently reported as PAs or OECMs. It also does not account for the non-random spatial location of these areas and how this may influence their effectiveness in preventing biodiversity loss. Addressing these limitations should be a priority for future research at the global scale.

In our analysis of the Peruvian Amazon, we found that 3.5% of 2005 forest extent was lost between 2005 and 2021. Despite this widespread loss, PAs and other governance mechanisms, including ILs and NTCs, have played a significant role in avoiding greater forest loss. The forest that is retained by these governance mechanisms stores large amounts of atmospheric carbon, which is key to climate regulation^73^. The estimation of the amount of carbon emissions avoided is a key measure to understand the role of these governance mechanisms in carbon cycles and regional climate regulation^22,73^. Additionally, these estimations are required to access REDD+ and other funding streams (e.g., from the Global Environmental Facility and the World Bank)^62,74^. Our results support recent findings that show that OECMs can be a valuable tool for achieving global climate and biodiversity goals^75^.

While our results show that different governance mechanisms in the Peruvian Amazon can reduce forest loss, we also found that conservation effectiveness varies substantially across sites within the same governance mechanism and that site size can be associated with this variation. Because sites are the operational units of management, understanding how site-level characteristics such as size, boundary configuration, accessibility, and governance capacity are associated with conservation outcomes is important for policy and practice. In this study, matching was conducted at the pixel level to control for spatial confounding in deforestation risk, which precludes the inclusion of site-level attributes such as size in the matching process. Further research examining how site-level characteristics relate to variation in effectiveness would improve understanding the conditions under which different governance mechanisms deliver conservation benefits.

Identifying potential OECMs and assessing their conservation performance is essential for advancing area-based conservation beyond formally designated PAs^22,48,49^, which will be required to achieve Target 3 of the GBF^24^. Our results show that several individual ILs and NTCs in Peru can achieve long-term in situ conservation of biodiversity, thus meeting criterion six and seven required for their identification as OECMs^26^ and criterion B and C for their recognition and reporting^25^. The contribution of sites under these governance mechanisms to regional and global conservation and climate change mitigation goals should be quantified and acknowledged, especially in economic terms. Conservation funding can come from different sources, including government programs and carbon and biodiversity markets^76^. This funding could then be channelled to monitor the contributions that these governance mechanisms provide to biodiversity conservation and carbon sequestration into the future as well as to better understand under which circumstances they provide the best conservation outcomes.

From 2005 to 2021, ILs avoided 21% of the forest loss and 24% of the carbon emissions that would be expected if those areas were unprotected. These results show that ILs can provide important conservation benefits in terms of preventing forest loss and reducing carbon emissions and our findings align with previous research conducted in the Amazon^35,50,77^ and at global level^9^. While the effectiveness of ILs in preventing deforestation and carbon emission is lower than that of PAs, we found that bigger sites managed by Indigenous People tend to be more effective preventing forest loss. IL cover approximately 19% of the Peruvian Amazon (Supplementary Table 1). It is thus key to support Indigenous Communities in co-designing and accessing financial mechanisms that allow them to receive forest-based income through activities that do not harm biodiversity. It is also crucial to support these Indigenous Communities in the monitoring of biodiversity outcomes and in the control of unsustainable extractive activities like mining or logging, and of illegal activities like cultivation of illicit crops, which are harmful to biodiversity^78–80^. The influence that Indigenous territorial governance and management has on the regional trends of deforestation and carbon emissions is substantial. In an environmental justice logic, it is therefore also crucial to support the claims by Indigenous Peoples in the Amazon and globally, who frequently advocate for better recognition of their contribution to global conservation goals^9,78^. Acknowledging their contribution through the opening of spaces for their direct participation in environmental and forestry policy-making and through the provision of funding to support their manifold approaches to land governance is necessary. At the site level, the identification of the specific management practices that are effective in preventing deforestation and which factors stimulate this effectiveness is crucial, so that they can be scaled to other comparable contexts in the Amazon and beyond.

Further, while we found that LCs were not effective at preventing deforestation overall, several individual sites (especially large ones) had a positive effect in reducing deforestation. While LCs do not meet all the criteria to be recognized as OECMs, these concessions do constitute a considerable proportion of the Peruvian Amazon (approximately 7%), so can still make a significant contribution to other conservation goals like target 10 of the GBF^24^, which aims to ensure the sustainable management of forestry. Previous research shows that these concessions can have a positive impact on reducing forest loss, especially those certified under responsible forestry schemes^50,81–83^. These certified LCs can prevent widespread illegal logging activities due to an incentive to defend forest assets, and timber profits, by excluding other illegal and legal actors interested in extractive activities (e.g., logging, mining, or land-grabbing)^82,83^. Despite the potential benefits of well-managed LCs in comparison to other extractive activities, it is still important to note that any logging has a negative impact on tropical forest ecosystems, as the extraction of trees degrades the forest^39,84^.

To further enhance the beneficial outcomes of LCs to people, beyond the economic benefits from the extraction of wood^81,83^, there could be other economic activities, such as agroforestry and tourism, with a comparatively lower impact on ecosystems than through other land uses like large-scale agriculture or mining. By turning LCs into multifunctional forest concessions, they could be contributing more to a sustainable and diversified regional economy. However, verification and monitoring must be enforced for these schemes to deliver benefits for people and nature^85^. While there is evidence that the effectiveness of LCs in preventing forest loss is higher in areas with higher deforestation pressure^82^, more studies on LCs are needed to understand what factors and which management conditions help reducing forest loss.

Our analysis comparing the effectiveness of potential OECMs preventing forest loss in the Peruvian Amazon provides an overview of the impact of these governance mechanisms avoiding deforestation and its associated carbon emissions, but there are some caveats. First, for this analysis, we assessed the most widely distributed governance mechanisms in the Peruvian Amazon, but other governance mechanisms that could potentially be listed as OECMs, including private^86^ and regional^87^ conservation areas, were not included in our assessment. In future studies, it would be valuable to evaluate their impact on biodiversity conservation and assess their potential to be listed as OECMs. Second, while there were no PADDD events in the Peruvian Amazon during the time of our analysis, there is evidence showing that these events are associated with increased deforestation^71^, human pressure^88^, and ineffective protection^89^. It is thus crucial to improve the understanding of how abandonment events may impact the achievement of the GBF goals^24^, and to assess the impacts of abandonment on OECMs and potential OECMs globally^31,32^. Third, while for our analysis we excluded cases where there was an overlap between sites with different governance mechanisms, it is important to highlight that previous studies have found that in areas of overlapping land governance, there is lower deforestation^90^. Cases of overlapping land allocations in Peru are mostly unintentional and commonly due to lack of coordination between different government bodies. This problem shows the need for an unified cadastre in the country^91^. More broadly, there is a need for a better understanding of the causes and consequences of overlapping land governance for biodiversity conservation^90^.

The GBF includes a target of 30% of land protected by 2030 and refers to OECMs as a complementary conservation approach to PAs^24^. Our study highlights the importance of PAs and OECMs as tools for achieving global climate and biodiversity goals. At the global level, we show that OECMs have higher human pressure levels than PAs, but much lower levels than unprotected land, and that many individual OECMs have very low human pressure levels. Our analysis at the level of the Peruvian Amazon provide robust evidence of long-term conservation impacts in both PAs and other governance mechanisms that have the potential to be listed as OECMs. While our results show that PAs are the most effective mechanism for preventing forest loss and carbon emissions, we also found that there are other governance mechanisms that can deliver conservation benefits at different extents and with different management characteristics. We also show how the variation in effectiveness preventing forest loss is considerable between sites and that this effectiveness can vary depending on the type of management and the size of the sites managed. These types of analysis could be replicated in other countries and landscapes to identify sites under governance mechanisms that can deliver conservation benefits. The development of a unified framework for conducting effectiveness assessments and for consistently monitoring the biodiversity benefits provided by these sites over time and across different contexts would facilitate the designation and long-term monitoring of OECMs globally^20,85^. In parallel, it is crucial to ensure long term financing and the development of mechanisms that address conservation abandonment to ensure that both OECMs and PAs deliver sustained conservation benefits over the long term^31,32^. Building on this, further research is needed to better understand how the effectiveness of different governance mechanisms varies across regions, particularly within South America and more broadly across the Global South. Governance arrangements operate within distinct socio-political, economic, and ecological contexts, which may influence their conservation performance and long-term stability. Comparative analyses across countries and regions would help identify the institutional, financial, and socio-environmental conditions under which area-based conservation measures are most effective. Such work would not only improve understanding of regional variation in conservation outcomes but also support more context-sensitive policy design and implementation, ensuring that governance mechanisms are tailored to local realities while contributing to global biodiversity and climate goals. To allow nations to more effectively achieve the different targets from the GBF and the UN Framework Convention on Climate Change^56^, it will be crucial to (i) better understand how different types of governance mechanisms operate and under which circumstances they can provide the most beneficial conservation outcomes, and (ii) strengthen their management and the monitoring of their biodiversity benefits^85^ through funding mechanisms specific to each governance type.

## Methods

### Human pressure in OECMs and PAs globally

Data on PA and OECM location, boundaries, and year of designation were obtained from the February 2025 World Database on PAs (WDPA)^10^ and OECMs (WDOECM)^11^. Following similar global studies^12^, we extracted areas from these databases by selecting those areas that have a status of “designated”, “inscribed”, or “established”, and that were not designated as UNESCO Man and Biosphere Reserves. We included only areas with detailed geographic information in the database, excluding those represented as one point only. Because few countries have so far reported on OECMs, we restricted our OECMs and PAs comparison to those countries that have reported OECMs, so we extracted PA data only for countries that also contained reported OECMs.

Many PAs overlapped spatially but contained different IUCN categories. To eliminate these overlaps and avoid double-counting PAs, we used the wdpar package^92^ in *R* (version 3.5.1) software^93^, assigning each overlapping area the IUCN status from the most recently designated polygon in that area. From this base dataset, we selected only those OECMs and PAs polygons bigger than 1 km² in an attempt to minimise miscalculations due to data resolution issues. The human pressure data used in our calculations is 300 m² resolution, so excluding areas smaller than 1 km² was used to remove areas that contain less than 3 pixels of human pressure data, on average. We removed any OECM or PA without a designation date, and those with less than 5% overlap with the human pressure data from ref. ^66^. (described in detail below). For the analysis, we grouped PAs into two groups. The ones with strict biodiversity conservation objectives (IUCN categories I and II) and the ones permitting certain human activities and sustainable resource extraction (IUCN categories III to VI)^12^.

We used data from ref. ^66^ to measure human pressure within PAs. This data, known as the revised human footprint map, is a globally-standardized and temporally comparable measure of cumulative human pressure on the terrestrial environment at a 300 m² resolution. It follows the human footprint methodology first established in refs. ^94,95^, but updates the methods to cover the period 2001–2020 by making use of new datasets and remote sensing advances. We used the human footprint information for the year 2020 as it provides the most up to date information on the extent and intensity of human pressure within the sites already listed as OECMs.

The human footprint provides a cumulative score of a range of anthropogenic pressures within five major categories: population density, land cover/land use, built infrastructure, accessibility (roads, railways and navigable waterways) and nighttime lights. To create the human footprint map, individual pressures are weighted according to estimates of their relative impacts on the environment, ranging from 0 to a maximum of 10. Some pressure span the full 0–10 range, whereas others have lower maximums (e.g., 8). The standardized scores are then summed, giving a cumulative score of human pressure ranging from 0 to 64 for each 300 m² area (while some pressures are mutually exclusive, others can co-occur). We refer to these 0–64 scores as Human Footprint (HFP) hereafter.

To examine the area of land under different levels of human pressure, we followed ref. ^96^ and divided the data into three illustrative pressure categories, which we describe as areas of low, medium and high human pressure. Previous work has shown that the area of species ranges with a HFP value > 3 (using ref. ^95^. data) is a strong indicator of extinction risk in mammals globally^97^. However, because refs. ^96,97^. used data from ref. ^95^ rather than ref. ^66^, and the scales of the two human footprint datasets used vary at the upper limit (0–50 vs 0–64), we ran a spatial regression between the two datasets to determine appropriate thresholds to use with the data from ref. ^66^ (Supplementary Fig. 1). The results show a high and significant correlation between the two human footprint datasets (*p*-value < 0.001; *R*^2^ = 0.7966), so we used the equation from this linear model to determine values from ref. ^66^. that correspond with previously used values from ref. ^95^. This analysis showed that values from ref. ^95^ of 3 and 7 correspond with HFP values of 3.13 and 6.69 in ref. ^66^, respectively. As such, we used these values to develop three categories of human pressure based on ref. ^66^: low pressure (HFP 0–3.13), medium pressure (HFP 3.13–6.69), and high pressure (HFP > 6.69). For each OECM and PA, we used the terra package^98^ in *R* (version 3.5.1) software^93^ to calculate mean HFP scores, as well as the area under each pressure category described above.

To examine differences in human pressure levels within OECMs and PAs, we conducted Wilcoxon rank-sum tests to assess the significance of differences in mean HFP values across OECM and PA categories. OECM and PA category was treated as the predictor variable and mean HFP as the response variable. The Wilcoxon rank-sum test, a nonparametric alternative to a one-way ANOVA, was used because the data did not meet the normality assumption required for parametric analysis.

### Potential of alternative governance mechanisms to be listed as OECMs

For the Peruvian case study, we defined the study area by using the Peruvian Ministry of Environment delimitation of the Peruvian Amazon^99^, which includes low elevation areas as well as the Amazon Andes foothills. We used information on the spatial distribution and establishment dates of government-managed PAs defined by the Peruvian PA Service—SERNANP^100^. As it was the case for the global analysis, here we also grouped PAs into the ones with strict biodiversity conservation objectives (IUCN categories I and II) and the ones permitting certain human activities and sustainable resource extraction (IUCN categories III to VI)^12^. For the spatial distribution of ILs, we made a request of information to the Ministry of Culture^101^. Details on ILs recognition dates were obtained from the database of Indigenous and Native Peoples^102^. For the spatial distribution of forest concessions, we used information from the National Forest and Wildlife Service—SERFOR^103^ and from a request for information to the regional governments of Madre de Dios, Loreto and Ucayali. When there were discrepancies with SERFOR, information from regional governments was used, as these entities were judged to have a more detailed record of their jurisdiction than national entities. Details on forest concession’s establishment dates were obtained from the Forestry and Wildlife Resources Supervision Agency^104^. Information on MCs spatial distribution, start, and closure dates was obtained from the mining and geological geoportal from the Ministry of Energy and Mines^105^.

We divided forest concessions into two groups based on the purpose of the concession and the definition of potential OECMs^25–27^. We aggregated those concessions that in principle do not use timber products as a main source of income, including conservation, ecotourism, reforestation, restoration and agroforestry into a single category (NTCs). These concessions meet the criteria for potential OECMs^25,26^. LCs, on the other hand, were assessed separately as their main objective is the commercial extraction of wood, which does not align with criterion six required for their identification^26^ and criterion C required for their recognition as OECMs^25^. These two criteria specify that sites that are subject to environmentally damaging industrial activities cannot be listed as OECMs.

To track deforestation, we used maps of forest cover for 2000 and forest loss from 2000 to 2021, developed by the Peruvian Ministry of the Environment and the Ministry of Agriculture and Irrigation^106^. These maps are at 30 m resolution base grid (WGS 84/UTM zone 18S), with each pixel classified as either forest or non-forest in the year 2000, and forest loss years represented as values of 1–21. For deforested areas, pixel values represent the year when the forest cover in that pixel was lost. The classification of a pixel into either forest or no forest is based on different Landsat images of the Peruvian Amazon for each year, which are then processed at a pixel level. The accuracy of these maps was calculated by randomly selecting 3000 sample pixels and contrasting the estimated transitions from 2000 to 2011 (no change or forest loss) with visual observation of high-resolution satellite images^60^. The estimated accuracy was 92.2% for forest loss and 99.5% for no change^60,107^. We quantified the extent and rate of forest loss for each year between 2005 and 2021 and the total cumulative loss between 2005 and 2021 for the whole study area and individually for each governance mechanism and each site.

For carbon, we used high-resolution above ground carbon density estimations for Peru, generated by ref. ^108^, to calculate metric tons (Mg) of carbon by forest pixel. These carbon estimations integrate countrywide ecosystem sampling using airborne light detection and ranging (LiDAR) with high-resolution satellite imaging^108^. Previous studies using LiDAR to estimate carbon density have found a precision and accuracy similar to estimates based on field measurements^109^. The carbon estimations in ref. ^108^ had an agreement of 74% with field-based carbon estimations from 51 vegetation plots in the study area. We assumed that if a pixel was deforested, all its carbon was emitted into the atmosphere. Then we summed the amount and proportion of carbon emissions by year and cumulative between 2005 and 2021 for the whole landscape, and for each governance mechanism as well as for the selected control and treatment pixels after the matching analysis. In this study, “carbon emissions” refers to emissions “committed” at the time of disturbance or clearing, noting that there may be a time lag until they are “realized”. Below ground carbon is not included in these estimations, but has been estimated to account for an average 22% in forest ecosystems^110^.

To account for the determinants of deforestation pressure and the location of the sites under different governance mechanisms, we used a suite of biophysical and socioeconomic confounding factors that have been found to be associated with the non-random spatial distribution of these interventions and of the distribution of deforestation risk in the Peruvian Amazon and other tropical landscapes^50,62,63,111^. These confounding factors were estimated at the pixel level and included in the matching analysis. They included measures of forest accessibility (i.e., distance to roads, rivers and previous deforested areas), anthropogenic pressure (i.e., distance to settlements, travel time to cities, human population density), suitability of conversion to agricultural land (i.e., precipitation, precipitation seasonality, ecoregion, elevation and slope) and the socio-political environment (i.e., administrative region). We also included the deforestation trends around each pixel between 2000 and 2005 at local (one-kilometre radius) and landscape (ten-kilometre radius) scale as covariates in the matching analysis to ensure our matched pixels had comparable deforestation trends prior to the study period (Table 1). Spatial information that was at a different spatial resolution was resampled to the forest pixels’ resolution of 30 by 30 m.

**Table 1:** Variables used for the matching analysis

Variable description	Variable name	Year	Resolution	Source
Annual precipitation	Annual_Prec	Historical	30 m	https://www.worldclim.org/
Precipitation seasonality	Prec_Seas	Historical	30 m	https://www.worldclim.org/
Distance to nearest previous deforested area	Dis_Def	2005	30 m	Ministerio del Ambiente (MINAM) & Ministerio de Agricultura y Riego (MIDAGRI)
Distance to nearest navigable river	Dis_Rivers	2021	30 m	Sistema Nacional de Información de Recursos Hídricos (SNIRH) from the Autoridad Nacional del Agua (ANA)
Distance to nearest district road	District_R	2018	30 m	Ministerio de Transportes y Comunicaciones (MTC)
Distance to nearest department road	Departme_R	2018	30 m	Ministerio de Transportes y Comunicaciones (MTC)
Distance to nearest national road	National_R	2018	30 m	Ministerio de Transportes y Comunicaciones (MTC)
Distance to nearest settlement of more than 10 people	D7Set10	2007	30 m	Instituto Nacional de Estadística e Informática (INEI)
Distance to nearest settlement of more than 1000 people	D7Set1000	2007	30 m	Instituto Nacional de Estadística e Informática (INEI)
Distance to nearest settlement of more than 5000 people	D7Set5000	2007	30 m	Instituto Nacional de Estadística e Informática (INEI)
Distance to nearest settlement of more than 10,000 people	D7Set10000	2007	30 m	Instituto Nacional de Estadística e Informática (INEI)
Distance to nearest settlement of more than 10 people	D17Set10	2017	30 m	Instituto Nacional de Estadística e Informática (INEI)
Distance to nearest settlement of more than 1000 people	D17Set1000	2017	30 m	Instituto Nacional de Estadística e Informática (INEI)
Distance to nearest settlement of more than 5000 people	D17Set5000	2017	30 m	Instituto Nacional de Estadística e Informática (INEI)
Distance to nearest settlement of more than 10,000 people	D17Se10000	2017	30 m	Instituto Nacional de Estadística e Informática (INEI)
Ecoregions	Ecoregions	–	–	Ministerio del Ambiente (MINAM)
Departments of the Peruvian Amazon	Department	–	–	Plataforma Nacional de Datos Abiertos
Elevation	Elevation	–	30 m	NASA Shuttle Radar Topography Mission
Slope	Slope	–	30 m	NASA Shuttle Radar Topography Mission
Population density	Pop2000	2000	1 km	https://www.worldpop.org/
Population density	Pop2020	2020	1 km	https://www.worldpop.org/
Travel time to major cities	Tra_Time00	2000	1 km	https://forobs.jrc.ec.europa.eu/gam
Travel time to major cities	Tra_Time15	2015	1 km	https://www.nature.com/articles/s41597-019-0265-5
Deforestation trends from 2000 to 2005 in 1 km buffer	Defor_2000_2005_1km2	2000	30 m	This study
Deforestation trends from 2000 to 2005 in 10 km buffer	Defor_2000_2005_5km2	2000	30 m	This study

We used forest loss between 2005 and 2021 as the outcome variable for our matching analysis. We excluded from the analysis those sites with governance mechanisms established after 2005, areas of overlap between sites with different governance mechanisms and sites that previously had one of the governance mechanisms in place but did not during the study period. Sites that stopped operating between 2005 and 2021 were included in the analysis. A supplementary analysis excluding the sites that stopped operating between 2005 and 2021 provided similar results (Supplementary Fig. 2). To avoid potential spill-over effects, particularly leakage^112,113^, we excluded all pixels within a buffer of 1 km (NTCs, LCs, MCs and ILs) and 5 km (PAs) around treatment sites^50^. The amount (km²) and percentage of land excluded from the control and treatment areas as well as the number of pixels in those areas is shown in Supplementary Table 1. In order to reduce autocorrelation, we generated a set of random samples at least 500 m apart from each other for all the sites under a particular governance mechanism and for the potential control units^62,114^. Potential control pixels were drawn from forested areas that were not assigned to any of the assessed governance mechanisms. While additional governance mechanisms with conservation potential exist in the Peruvian Amazon, they were not included in the analysis because they were implemented after 2005 or lack consistent spatial information at the Amazon scale^115^ (Supplementary Table 2). A flow diagram of the process for the definition of the pool of control pixels is presented in Supplementary Fig. 3.

To assess the impacts of the sites under each governance mechanism (treatments) on forest loss, we identified pixels with similar characteristics to the ones from the sites under a particular governance mechanism (controls) based on a set of defined covariates^63,116^. For each pixel, we extracted the values of the confounding variables associated with deforestation and the site location. This was done both for the treatment group (all the pixel in sites under a particular governance mechanism) and the potential control group (pixels that were not classified under any of the assessed governance mechanism). To determine the best minimum set of confounding variables, we first ran a binomial logistic regression model using each governance mechanism as the response variable (whether a pixel is inside that particular governance mechanism or not) and the confounding factors as predictor variables^117^. Then we ran a multicollinearity test to the coefficients of the covariates of each governance mechanism model. Through this analysis, we identified groups of variables with a collinearity higher than 0.65. The variable with the lowest Variance Inflation Factor for each group was retained in the model^50,118^ (Supplementary Figs. 4–9 and Supplementary Tables 3–8). We then used “backward” and “forward” techniques in an iterative stepwise process for model selection, to identify the optimal model. The model that achieved the greatest reduction in the Akaike’s Information Criterion (AIC) was selected^50^ (Supplementary Figs. 10–16). For this, we used the leaps package^119^ in *R* (version 3.5.1) software^93^. The variables from the best model were used in the subsequent analyses and for the matching procedure for each governance mechanism.

We explicitly accounted for spatial autocorrelation by estimating the residual spatial autocorrelation of these models (Moran’s I) and then removing the pixels highly autocorrelated from the analysis. We did this through the use of a global Moran’s I to estimate the level of spatial autocorrelation between pixels in our case study area (the Peruvian Amazon) and a local Moran’s I to identify locally the pixels with the highest spatial autocorrelation^120^. We then iteratively removed the pixels with the highest autocorrelation until the global Moran’s I was non different from 0 at a distance below 1000 meters^63,117^ (Supplementary Figs. 17–22).

We then used the MatchIt package^121^ in the *R* statistical software (version 3.5.1)^93^ to pair treatment and control pixels with similar values for each of the covariates^93^. We used Propensity Score Matching to create a statistically balanced counterfactual sample to evaluate the effect of each governance mechanism on forest loss. Despite its limitations, Propensity Score Matching remains the most widely used matching approach^62,63,116,117^. For each governance mechanism, we conducted an independent matching analysis in which each treatment pixel was paired with a single control pixel from the pool of eligible controls. Matched pairs of pixels had to be within 0.2 SD of the propensity scores^122,123^. Matching was performed without replacement within each governance mechanism analysis, meaning that a control pixel could only be used once in that specific matching exercise. However, the same overall pool of eligible control pixels was used across the separate matching analyses conducted for each governance mechanism.

We discarded any treatment pixel where the distance in propensity scores between treatment and control was >0.2 to remove potential outliers^61,122^. We then calculated the indices of covariate imbalance for every matching analysis for each governance mechanism to assess the robustness of the matching analysis, reducing bias in the control units. Absolute scores >25% are considered an indication of a possible imbalance for that specific variable^63,117,124^. All covariate imbalances had absolute scores >25% post-matching with notable improvements reducing bias in the control units (Supplementary Figs. 23–29). By including deforestation trends between 2000 and 2005 at both the local (1-km radius) and landscape (10-km radius) scales as matching variables, we ensured that treatment and control pixels had similar patterns of forest loss before the study period began. In other words, matched pixels were already experiencing comparable deforestation trends prior to 2005. This improves confidence that differences observed after 2005 are attributable to governance mechanisms rather than pre-existing differences in forest loss patterns (Supplementary Figs. 30–32). We then compared forest loss (2005–2021) within control and treatment areas. We estimated summary statistics by calculating average deforestation based on pixels (which up-weigh larger sites). We also did the summary statistics at site level by first summarizing deforestation rates in all pixels within each site before estimating the overall effects based on average values across sites (which up-weighs small sites)^61^. We also calculated the avoided deforestation by each site and by governance mechanism by subtracting the average amount of forest loss in the control pixels from the average amount of forest loss in the treatment pixels. Positive avoided deforestation values imply that the site or governance mechanism was effective avoiding deforestation (higher deforestation in the controls than in the treatment areas), while negative avoided deforestation values imply that the site or governance mechanism was ineffective preventing deforestation (higher deforestation in the treatment than in the control pixels). To assess the effect of each governance mechanisms reducing deforestation, we used general linear mixed models with a binomial response (loss = 1, no loss = 0) and the status of each pixel as the predictor variable (inside vs. outside a site with a particular governance mechanism).

### Reporting summary

Further information on research design is available in the Nature Portfolio Reporting Summary linked to this article.

## Supplementary information


Supplementary Information
Reporting Summary
Transparent Peer Review file


## Data Availability

All data used in this study are publicly available or can be obtained from the sources listed in Table 1. Some datasets provided by Peruvian government agencies are not publicly available and must be requested directly from the relevant authorities, as indicated in Table 1.

## Code availability

The code and files to run the analysis in this study can be found in the repository^125^; https://zenodo.org/records/20077873.
