# Supplementary material for: Potential of different area-based governance mechanisms for achieving Global Biodiversity Framework goals
Source: Nat Commun. 2026 Aug 3;17:9337. doi: 10.1038/s41467-026-76225-9 (PMC13529737; doi:10.1038/s41467-026-76225-9)
Supplement: Supplementary file 1 [file 41467_2026_76225_MOESM2_ESM.pdf]

nature portfolio

Corresponding author(s): Pablo Negret

Last updated by author(s): May 22, 2026

Reporting Summary

Nature Portfolio wishes to improve the reproducibility of the work that we publish. This form provides structure for consistency and transparency in reporting. For further information on Nature Portfolio policies, see our Editorial Policies and the Editorial Policy Checklist.

Statistics

For all statistical analyses, confirm that the following items are present in the figure legend, table legend, main text, or Methods section.

n/a	Confirmed	
☐	☒	The exact sample size (*n*) for each experimental group/condition, given as a discrete number and unit of measurement
☐	☒	A statement on whether measurements were taken from distinct samples or whether the same sample was measured repeatedly
☐	☒	The statistical test(s) used AND whether they are one- or two-sided<br>*Only common tests should be described solely by name; describe more complex techniques in the Methods section.*
☐	☒	A description of all covariates tested
☐	☒	A description of any assumptions or corrections, such as tests of normality and adjustment for multiple comparisons
☐	☒	A full description of the statistical parameters including central tendency (e.g. means) or other basic estimates (e.g. regression coefficient) AND variation (e.g. standard deviation) or associated estimates of uncertainty (e.g. confidence intervals)
☐	☒	For null hypothesis testing, the test statistic (e.g. *F*, *t*, *r*) with confidence intervals, effect sizes, degrees of freedom and *P* value noted<br>*Give P values as exact values whenever suitable.*
☒	☐	For Bayesian analysis, information on the choice of priors and Markov chain Monte Carlo settings
☒	☐	For hierarchical and complex designs, identification of the appropriate level for tests and full reporting of outcomes
☐	☒	Estimates of effect sizes (e.g. Cohen's *d*, Pearson's *r*), indicating how they were calculated

Our web collection on statistics for biologists contains articles on many of the points above.

Software and code

Policy information about availability of computer code

Data collection	Remote sensing and geospatial datasets used in this study were obtained from publicly available sources. Forest loss data were derived from Landsat-based satellite imagery products provided by the Peruvian Ministry of the Environment (MINAM) and related national forest monitoring systems. Spatial layers for governance mechanisms (Protected Areas, Indigenous Lands, and forest concessions) were obtained from official national geospatial databases and the World Database on Protected Areas (WDPA). Global datasets used for descriptive analyses, including the Human Footprint Index and the World Database on OECMs, were accessed from publicly available repositories. No custom software was used for data collection.
Data analysis	All spatial processing and statistical analyses were conducted using standard geospatial and statistical software. Spatial analyses and raster processing were performed using Geographic Information System (GIS) software and the R statistical environment. Matching analyses were implemented using established propensity score matching approaches described in the Methods section. Statistical analyses and regression models were implemented using R packages commonly used for causal inference and spatial analysis. Code used for the analysis will be made available at this link once the review process is finished; https://github.com/vicjulrin/Matching_Counterfactual_Forest/tree/Analysis_2005_2021

For manuscripts utilizing custom algorithms or software that are central to the research but not yet described in published literature, software must be made available to editors and reviewers. We strongly encourage code deposition in a community repository (e.g. GitHub). See the Nature Portfolio guidelines for submitting code & software for further information.

Data

Policy information about availability of data

All manuscripts must include a data availability statement. This statement should provide the following information, where applicable:

- Accession codes, unique identifiers, or web links for publicly available datasets
- A description of any restrictions on data availability
- For clinical datasets or third party data, please ensure that the statement adheres to our policy

The datasets used in this study are derived from publicly available sources. Forest loss data were obtained from the Peruvian Ministry of the Environment (MINAM) national forest monitoring system. Spatial data on protected areas were obtained from the World Database on Protected Areas (WDPA), and information on Other Effective Area-Based Conservation Measures (OECMs) was obtained from the World Database on OECMs maintained by UNEP-WCMC and IUCN. Spatial data on Indigenous Lands and forest concessions were obtained from official national geospatial databases of the Peruvian government. Derived datasets and analytical outputs supporting the findings of this study are available from the corresponding author upon request.

Research involving human participants, their data, or biological material

Policy information about studies with human participants or human data. See also policy information about sex, gender (identity/presentation), and sexual orientation and race, ethnicity and racism.

Reporting on sex and gender	Not applicable. No human participants were involved in this research.
Reporting on race, ethnicity, or other socially relevant groupings	Not applicable. No human participants were involved in this research.
Population characteristics	Not applicable.
Recruitment	Not applicable.
Ethics oversight	Not applicable.

Note that full information on the approval of the study protocol must also be provided in the manuscript.

Field-specific reporting

Please select the one below that is the best fit for your research. If you are not sure, read the appropriate sections before making your selection.

☐ Life sciences ☐ Behavioural & social sciences ☒ Ecological, evolutionary & environmental sciences

For a reference copy of the document with all sections, see nature.com/documents/nr-reporting-summary-flat.pdf

Ecological, evolutionary & environmental sciences study design

All studies must disclose on these points even when the disclosure is negative.

Study description	This study evaluates the effectiveness of different area-based governance mechanisms in preventing forest loss and associated carbon emissions. The analysis combines a global descriptive assessment of human pressure within areas reported as OECMs with a quantitative impact evaluation conducted in the Peruvian Amazon. For the regional analysis, we compare forest loss and carbon emissions across several governance mechanisms, including protected areas, Indigenous Lands, non-timber forest concessions, logging concessions and mining concessions. A matched control-intervention design was used to estimate avoided deforestation by comparing treated pixels with statistically matched control pixels with similar environmental and accessibility characteristics.
Research sample	For the regional impact evaluation in the Peruvian Amazon, the analytical units are 30 m forest pixels derived from Landsat-based remote sensing datasets, which allow fine-scale estimation of forest loss and carbon emissions within governance mechanisms. Governance mechanisms analysed include protected areas (IUCN categories I–VI), Indigenous Lands, non-timber forest concessions, logging concessions and mining concessions established prior to the study period.For the global descriptive analysis, spatial units correspond to 300 m resolution grid cells derived from the Human Footprint Index, which provides a temporally comparable measure of cumulative human pressure on the terrestrial environment. These data were used to evaluate levels of human pressure within globally reported OECMs and protected areas.
Sampling strategy	The study uses a quasi-experimental matching design to estimate causal impacts of governance mechanisms on forest loss. Propensity score matching was used to match treatment pixels with control pixels based on covariates related to deforestation pressure, including accessibility, environmental characteristics and baseline conditions. Matching ensured that treatment and control samples had similar observable characteristics prior to analysis. Sample size was determined by the spatial extent of governance mechanisms and available forest pixels within the study area.
Data collection	Forest loss was derived from satellite-based remote sensing products generated from Landsat imagery. Carbon density estimates were obtained from previously published high-resolution carbon maps for Peru. Spatial datasets describing governance mechanisms were compiled from national geospatial databases and international conservation databases. All spatial data were processed and analysed using GIS and statistical software.

the global descriptive analysis uses the Human Footprint Index, a temporally comparable measure of cumulative human pressure on the terrestrial environment at a 300 m resolution, to assess levels of anthropogenic pressure within areas reported as OECMs and protected areas.

Timing and spatial scale	Forest loss and carbon emissions were analysed for the period 2005–2021 across the Peruvian Amazon, using spatial units of 30 m forest pixels derived from satellite imagery. The global descriptive analysis of OECMs used the most recent available datasets on OECM (2025) coverage and human pressure (2020).
Data exclusions	Sites with governance mechanisms established after 2005 were excluded from the analysis to ensure comparability across the study period. We also excluded areas where different governance mechanisms spatially overlapped and sites that previously had a governance designation but did not during the study period. Sites that ceased operating between 2005 and 2021 were retained in the primary analysis; however, a supplementary analysis excluding these sites produced similar results (Supplementary Fig. 3).To avoid potential spill-over effects, particularly leakage, we excluded all pixels within a buffer of 1 km around non-timber forest concessions, logging concessions, mining concessions and Indigenous Lands, and 5 km around protected areas. The total area and percentage of land excluded from treatment and control areas are reported in Supplementary Table 1.
Reproducibility	All datasets used in the analysis are derived from publicly available sources or national geospatial databases. The analytical workflow relies on standard spatial analysis and statistical methods described in the Methods section. The use of publicly available datasets and established statistical methods enables replication of the analysis.<br>Code used for the analysis will be made available at this link once the review process is finished;<br>https://github.com/vicjulrin/Matching_Counterfactual_Forest/tree/Analysis_2005_2021
Randomization	Randomization was not applicable because this study uses an observational quasi-experimental design. Instead, statistical matching techniques were used to control for observable differences between treatment and control pixels.
Blinding	Blinding was not applicable because the study relies on observational spatial data derived from remote sensing and publicly available geospatial datasets.

Did the study involve field work? ☐ Yes ☒ No

Reporting for specific materials, systems and methods

We require information from authors about some types of materials, experimental systems and methods used in many studies. Here, indicate whether each material, system or method listed is relevant to your study. If you are not sure if a list item applies to your research, read the appropriate section before selecting a response.

Materials & experimental systems

n/a	Involved in the study
☒	☐ Antibodies
☒	☐ Eukaryotic cell lines
☒	☐ Palaeontology and archaeology
☒	☐ Animals and other organisms
☒	☐ Clinical data
☒	☐ Dual use research of concern
☐	☒ Plants

Methods

n/a	Involved in the study
☒	☐ ChIP-seq
☒	☐ Flow cytometry
☒	☐ MRI-based neuroimaging

Dual use research of concern

Policy information about dual use research of concern

Hazards

Could the accidental, deliberate or reckless misuse of agents or technologies generated in the work, or the application of information presented in the manuscript, pose a threat to:

No	Yes
☒	☐ Public health
☒	☐ National security
☒	☐ Crops and/or livestock
☒	☐ Ecosystems
☒	☐ Any other significant area

Experiments of concern

Does the work involve any of these experiments of concern:

No	Yes	
☒	☐	Demonstrate how to render a vaccine ineffective
☒	☐	Confer resistance to therapeutically useful antibiotics or antiviral agents
☒	☐	Enhance the virulence of a pathogen or render a nonpathogen virulent
☒	☐	Increase transmissibility of a pathogen
☒	☐	Alter the host range of a pathogen
☒	☐	Enable evasion of diagnostic/detection modalities
☒	☐	Enable the weaponization of a biological agent or toxin
☒	☐	Any other potentially harmful combination of experiments and agents

Plants

Seed stocks	Not applicable
Novel plant genotypes	Not applicable
Authentication	Not applicable
