# Supplementary material for: Potential of different area-based governance mechanisms for achieving Global Biodiversity Framework goals
Source: Nat Commun. 2026 Aug 3;17:9337. doi: 10.1038/s41467-026-76225-9 (PMC13529737; doi:10.1038/s41467-026-76225-9)
Supplement: Supplementary file 2 [file 41467_2026_76225_MOESM3_ESM.pdf]

nature portfolio

Peer Review File

Potential of different area-based governance mechanisms for achieving Global Biodiversity Framework goals

Corresponding Author: Dr Pablo Negret

This file contains all reviewer reports in order by version, followed by all author rebuttals in order by version.

Attachments originally included by the reviewers as part of their assessment can be found at the end of this file.

Version 0:

Reviewer comments:

Reviewer #1

(Remarks to the Author)
This is an interesting ms but one which still needs a deal of work. Detailed comments are below, but overall, the ms could do with reframing and sharpening the arguments. More needs to be explained as well as to how a geographically narrow focus could be upscaled.
In Abstract: Introduce acronyms in full first e.g. PAs
L46 if Pas only avoid 88% rather than the expected 100%, how is forest lost in a PA?
what is the problem here?
L50 – they are actual, not potential. And what other mechanisms?
L60 Tropical forests do have much species richness, not just in vertebrates. It is also true they store carbon, but increasing work shows wetlands (even ones in temperate zones) are the most effective ecosystems at sequestering carbon.
L66 the figure is closer to 18%.
L67 if Pas are not effective then maybe they are not working? What does low or medium integrity mean?
L75: What type of socioeconomic conflicts?
L80 - 82: This is great, but some examples could be helpful.
L80 perhaps cite the ipbes global assessment (2019) which has detail on this.
L104 seems to repeat some of the discussion above.
L111 conservation and carbon surely?
L 156 OECMs are defined by the CBD in Decision 14/8 as “a geographically defined area other than a Protected Area, which is governed and managed in ways that achieve positive and sustained long-term outcomes for the in-situ conservation of biodiversity, with associated ecosystem functions and services and where applicable, cultural, spiritual, socio–economic, and other locally relevant values”. This is the reference that is preferable to use.
L162 – such sites as you mention may have long-term potential for returning positive biodiversity and Carbon results?
L173: How was the accuracy assessment conducted?
L177 et sq this sems a very rough way to deal with Carbon…
L179 – it would be important to understand the extent of soil carbon that is left after clearing. Maybe it is extremely low, but it should be mentioned.
L185 – I am unfamiliar with cofounding factors – should it be confounding?
L198 – the statistical matching process seems appropriate but there are doubts as to the robustness of data being analysed based on the earlier descriptions.
L216 should be were, not where
L251 – it is interesting that PAs showed any loss at all – I think more could be made of this. See l67 comments
L283 et seq – these are interesting findings! I think more explanation would be warranted.
L300 this does not chime with the statements in 283 noted above or am I missing something?
L314 – conservation funding... this is a complex topic – what funding is foreseen here? National? International? And it is surely positive conservation and carbon outcomes that are sought?
L322 this seems a little confused – is the idea for carbon credits< or direct funding from GEF/WB?
L331-37 – this seems at variance with the lines above? And rather confused argumentation.
L341 – here the paper moves into the delicate territory of ILK, and the need for FPIC. I would suggest this reformulated or even omitted.

L351 et seq. Yes, these conclusions are significant. The problem with OECM is that it is difficult to see where they end…
L357 – here it depends on intensity. Limited and targeted logging may have positive effects.
L375-76 this needs reformulating previous, and posterior appear to mean the same?
L385 this may not be universally true… disturbed forest can have dense regrowth.
L392 – this is a bold claim that may not be accurate.

(Remarks on code availability)

Reviewer #2

(Remarks to the Author)

Major comments
1. This manuscript addresses an important topic – the relative impact on deforestation rates and carbon emissions of different land management regimes. The topic has been studied by others (see Lambin, Blackman, etc.), but scientific understanding remains in its infancy.
2. The manuscript does not address *survivorship bias* in its sample of governance systems. That is, the sample does not appear to include interventions that were established and subsequently abandoned or revoked. If not, then the study likely overstates the impact of these interventions (e.g., protected areas) because it examines the impacts of ONLY those sites that persisted throughout the study period – and excludes those that did not "survive" until 2021 (the end of the study period). At the least, this should be acknowledged as a limitation of the study. See the robust literature on PADDD (Protected Area Downgrading, Downsizing, and Degazettement) by Mascia and others, including Mascia and Pailler (2010), Forrest et al (2014), Pack et al (2016), Tesfaw et al (2018), Golden Kroner et al (2019), and Keles et al (2020, 2023) and the PADDD data for Peru (available via PADDDtracker.org). We also know that forest certification/concessions, IL rights, and other forms of governance studied here can be revoked or modified.
3. More generally, the temporal elements of the study are not clear. Is the conservation impact measured at t+1 (where t = date of establishment for the protected area, etc) or assessed based on the full time period of the ecological data (2000-2001)? Baseline is presumably pre-establishment year or years, with impact as post-establishment, but it isn't clear that the data are analyzed in this way. At the least, clarification is necessary; if the impact isn't estimated on purely post-establishment years, then modifications to the analyses are required. This comment is relevant to all treatments/governance systems studied.
4. The manuscript would be stronger if it clarified what residual forms of governance are considered as potential control sites. Given all of the governance systems that are considered treatments, it isn't clear what other forms of governance exist in the Peruvian Amazon.
5. It would strengthen the manuscript if the discussion looked beyond Peru to consider the scientific and policy implications for tropical forests generally. Given the title focuses on a global question, the manuscript (including the discussion) should speak to the global.

Other comments
6. Note that Lambin et al have examined the impact of "stacked" interventions. At the least, this is worth mentioning. "stacked interventions" may also merit inclusion in this analysis as a form of governance.
7. Note the work of Pfaff and others on the impacts of FSC certification on deforestation in Peru and elsewhere.
8. Note that OECMs are a label applied to area-based governance systems for conservation accounting purposes, not a form of governance themselves. The manuscript generally avoids conflating the governance system (e.g., "forest concessions") with the OECM label – please continue to make this distinction and perhaps even make the point more clearly and forcefully.
9. Lines 72-74 imply that protected areas = "strict protection". In fact, protected areas include a wide range of governance and management systems, ranging from "fortress" to sustainable use.
10. The manuscript seems to focus on above ground carbon. Please clarify and acknowledge below ground carbon…
11. What is meant by "artificial control groups"? (lines 212) This makes me think of synthetic controls, yet lines 229-240 suggest a more traditional propensity score approach to matching. Perhaps clarify, especially for readers with only a basic understanding of statistical matching methods.
12. In the results, there are several points at which differences are reported… but then the manuscript notes that the differences are "not significant" (e.g., lines 280-281). If we're using statistical methods, do we talk about differences as meaningful if they aren't statistically significant? Please modify or justify.
13. The results talk about total and % forest loss across governance types without normalizing these values to account for discrepancies in the total area governed. This leads the reader astray, especially since the total area (and proportion) of land area held under these different regimes is not emphasized in the manuscript. Better to have the normalized data in the main text and a supplemental figure or table with the absolute values.
14. The writing is generally ok. Occasionally, however, awkward phrasing is distracting and can hinder understanding. It would strengthen the manuscript to have a careful copy edit by a native English speaker who is a very strong writer.

(Remarks on code availability)

Reviewer #3

(Remarks to the Author)

I co-reviewed this manuscript with one of the reviewers who provided the listed reports. This is part of the Nature Communications initiative to facilitate training in peer review and to provide appropriate recognition for Early Career Researchers who co-review manuscripts.

(Remarks on code availability)

Version 1:

Reviewer comments:

Reviewer #1

(Remarks to the Author)
I value the intense work undertaken by the authors in response to the reviews. I am content now that this could be a good ms for publication.

(Remarks on code availability)

Reviewer #2

(Remarks to the Author)
I appreciate the effort that has been invested in the revised manuscript. Some points raised in the original reviews have been resolved, some remain unresolved, and some new issues have emerged that need to be addressed to merit publication.

Critical issues to address –

1. Methods: Further clarification is required regarding treatment and control site selection. As I understand it: (i) the study area is the biogeographic boundary of the Peruvian Amazon; (ii) the treatment sites were all established prior to 2006 and fall into one of 5 governance systems (PAs, ILs, NTCs, LCs, or MCs); and (iii) potential control sites include the entire non-treatment area of the Peruvian Amazon, except for a buffer of 1 km (NTCs, LCs, MCs and ILs) and 5 km (PAs) around treatment areas.

This implies that the following areas are potential control sites:
a. Sites with multiple “stacked” treatments (e.g., indigenous lands with a logging concession)
b. Sites with other area-based governance systems in place with conservation intent or potential (e.g., payments for ecosystem services)
c. Sites that previously had one of these 5 “treatment” governance systems in place, but did not during the study period (e.g., PADDDed protected areas, canceled forest concessions).
d. Sites that established one of these 5 “treatment” governance systems during the period 2006-2025.

Is this list of potential control sites accurate? None of these potential control sites (a-d) can be considered true controls that are independent of the treatment, since they themselves are governance treatments that may affect the endpoints examined in this study (forest cover and carbon emissions). The estimated impacts resulting from such an analysis is confounded by such an analysis of “false controls”.

Please omit any false controls from the analysis and, in the methods, describe the residual forms of forest governance that remain within the pool of prospective control sites/pixels. Also, please clarify whether the pool of potential control sites is the same for all treatments or if there are different pools of potential control sites for each of the 5 treatments.

2. Methods. Line 265. As noted in my previous review, survivorship bias (i.e., only examining impact of interventions that survive/persist until the conclusion of the study period) can bias research findings (since “survivors” are dissimilar from non-survivors, given that the survivors have survived to the end of the study period!). Accordingly, in this case, mining concessions that stopped, were revoked, etc., during the study period should remain within the “treatment” group and results should be reported accordingly. (Since the team has already completed analysis with some discontinued mining concessions examined for impact, these “survivors only” results should be included in the supplemental materials.)

3. Methods: A control-intervention study design with matching is stronger than such a design without matching, but obviously not as strong as before-after-control-intervention. One method that helps ensure the validity of control-intervention designs is to test for and show parallel trends in outcome variables prior to the study period, yet I do not see mention of this in the methods. Am I missing it? If not, please conduct and include such a parallel trends analysis in the study design or justify why such an analysis is not necessary in this study.

4. Methods: lines 275+. Although commonly used when evaluating forest outcomes of conservation initiatives, the use of pixel-level comparisons to estimate treatment effects raises questions about pseudo-replication. That is, because protected areas, indigenous lands, and other area-based forest governance systems are managed at the site/location/entire jurisdiction level (e.g., an entire protected area, rather than at the level of a pixel within a protected area), the pixel-level

observations within a protected area are not independent of each other… and, thus, measurements of 500 pixels across 5 protected areas is an assessment of 5 independent treatments (not 500).

With these concerns in mind, the manuscript needs to specify the number of sites/locations/jurisdictions (e.g., protected areas) included for each treatment category, as well as the number of total observations made (pixels) for each treatment category and the distribution of these observations for each category across the number of sites. The manuscript also needs to address the issue of pseudo-replication, either by modifying its methods and/or by justifying its approach to sampling and analysis.

5. Results/methods – statistical matching (lines 343-353). The covariate imbalances for non-timber concessions (NTCs) are quite high, making use of these statistical models to estimate treatment effects highly concerning. Given the sensitivity of estimated treatment effects to match specifications, and the match imbalances reported here, further model refinement is necessary and/or further justification is required for reporting results that do not adhere to basic guidelines for use of propensity score matching methods. It may make sense to omit the NTC treatment group, since including a bad match could bias the reader's view of the entire study.

Likewise, given the substantial imbalance in "distance to settlement" for protected areas and their controls, further model refinement is necessary and/or further justification is required for inclusion of results from the protected area (PA) treatment.

6. Results & discussion – All mention of PAs and NTCs in the results and discussion need to be reconsidered based on refinements to study design and statistics. Discussion of logging concessions (LCs) and their potential as a conservation strategy also needs to be revisited, given the non-significant impact on forests and carbon emissions reported.

7. Discussion – Please discuss the study's findings in the context of previous research, rather than reiterating/re-summarizing the study's results (e.g., lines 387-393). There is mention of prior research by others, but it would strengthen the manuscript to go farther. What previous findings does this study reaffirm? For both science and policy, what prior findings/conventional wisdom does this study challenge, what new questions does it raise, and new directions does it suggest?

Other major comments:
8. Methods: The global analysis distinguishes between protected areas in IUCN categories I-II and those in categories III-VI. Given the differences among protected areas in these two subgroupings at the global level, the Peru-specific component of the manuscript should make a similar distinction or justify why such a distinction is not appropriate in the study design and analysis. At the least, descriptive data and discussion of regarding the Peruvian conservation treatments should include IUCN categories for the relevant protected areas.

9. Results: Lines 320-322 – The numbers here seem high (e.g., 2590 Indigenous Lands); do these numbers represent designated territories/legal designations, individual pixels, or something else? Please clarify. See Qin et al (2024): One Earth 7(6) 962-975

10. Results – line 346-347: imbalanced matching for PAs. Does this mean that pixelated observations within PAs were closer to settlements or farther from settlements, relative to the pixels against which they were matched? This obviously matters, given the impact estimate of dramatically reduced deforestation because of protected area establishment.

11. Discussion. Lines 380-381. "…we can conclude that…" This conclusion seems misguided, based on inference from the analysis. We know that OECMs and PAs are nonrandomly located (e.g., Joppa & Pfaff's "high and far" analysis), so this observation re: pressure is perhaps simply another way to confirm their observation, rather than an estimate of the impact of these conservation initiatives on human behavior/land use. Appropriate inference and interpretation of the global analysis is particularly important in this manuscript, given the use of matching methods to control spatial biases and allow for more rigorous causal inference.

Minor comments
12. Title: "…area-based governance mechanisms…"?
13. Keywords: include "protected areas"? omit something else?
14. Lines 55-57: the MS notes later that logging concession cannot be classified as OECMs because of their extractive orientation, yet highlight them here with "…potential to be listed as OECMs…" Please clarify/reconcile discrepancy.
15. Lines 78, 80, etc. Is "protection" used intentionally? "Protection" sometimes implies an exclusionary fortress model, whereas 'conservation' often implies a more diverse and potentially inclusive set of governance systems/strategies.
16. Lines 84-85. Note Fedele, et al. 2021. Global Environmental Change (71): more than 2 billion people in tropics depend on nature to meet their basic needs.
17. Lines 92-93. Note that Cook et al (OneEarth 7(3): 412-430) find that many protected areas around the world that have been reclassified as OECMs, now that there is a new accounting system that will allow governments to get credit for them. This observation should be considered and addressed with respect to the global analysis of human pressures within OECMs and PAs.
18. Line 96: "this land" – which land? unclear antecedent. Does the MS refer to the entirety of the earth's surface or to the 1% that is within recognized OECMs?
19. Line 100. This is misleading. Not all indigenous lands are recognized as OECMs. Please clarify.

20. Lines 100-116: reference to Qin et al (2024): One Earth 7(6) 962-975 seems relevant here and throughout the manuscript.
21. Line 108: “ranging from”, rather than “including”?
22. Lines 114-115: “could be listed..” could all of these governance systems be recognized as OECMs, given the criteria governing what can be recognized and what cannot?
23. Method: Lines 231. In what way is reforestation/forest regrowth addressed within the study design and analysis?
24. Lines 262+. Phrasing about “governance mechanisms” is misleading/confusing. “Governance mechanism” or “governance system” should refer an entire class or category of initiatives (e.g., “protected areas” or “indigenous lands”), with individual “designations” or “sites” or “locations” or “individual protected areas” representing the manifestation/implementation of these mechanisms on the ground. As currently phrased, "mechanism” appears to refer to individual sites, rather than the general systems/classification/family of initiatives to which these sites belong.
25. Line 268. The phrase “once designated they acquire this status in perpetuity” can probably be omitted. Protected areas are also designated “in perpetuity” and are conceived as such, yet we have seen widespread PADDD in Peru and around the world during the 20th and 21 centuries. Assertions of legal designations in perpetuity is not the same as empirical research to verify this point; please ensure empirically that no indigenous lands have been downsized or disestablished during the study period.
26. Lines 356-358. It seems inappropriate to say that the “results show that … LCs decrease forest loss compared to matched controls” but that these differences were not statistically significant.

Description of the impact of mining concessions is similarly problematic, especially since no p-value is reported in the results.

See this article on reporting numeric differences that are not statistically significant: https://onlinelibrary.wiley.com/doi/pdf/10.1111/jan.14283.

27. Writing: Further precision in language is necessary to communicate clearly with readers. Please re-read for vague/unclear antecedents, misleading statements, etc. (e.g., Line 54: “their” is unclear; line 74 “they” is unclear, line 111 “their”, etc.) Please review MS in full and specify clearly. Perhaps ask non-authors to read the manuscript and highlight ambiguous language.

(Remarks on code availability)

Reviewer #3

(Remarks to the Author)
I co-reviewed this manuscript with one of the reviewers who provided the listed reports. This is part of the Nature Communications initiative to facilitate training in peer review and to provide appropriate recognition for Early Career Researchers who co-review manuscripts.

(Remarks on code availability)

Version 2:

Reviewer comments:

Reviewer #2

(Remarks to the Author)
1. Thank you for the extensive careful revisions. The manuscript is much stronger. See below for remaining questions and concerns.

Major comments:
2. Potential control sites: Lines 294-295 are the most critical in the entire paper, as the matching process for control sites will dramatically shape estimated treatment effects. Though the pool of potential control sites/pixels is probably clear in the minds of the authors, these potential control sites are still not clear to the reader. Please include one or more figures (e.g., real or cartoon map; flow diagram, etc) that illustrate the residual forests/governance mechanisms that are eligible for matching as control pixels.
3. Spatial extent of land classifications used in the analysis: Please include a supplemental table that lists the total area (km2) and percentage of the study area (i.e., Peruvian Amazon) represented by each of the classifications used to identify or exclude potential treatment pixels and control pixels. In other words, how much land was within the buffer zones for each governance mechanism? How much land was within the “established after 2005” category for each mechanism? How much land was a viable treatment for each mechanism? How much was a potential control? Etc. It’s hard to understand exactly what’s going on in the analysis (and how to interpret the results) without this descriptive info.
4. Lines 394-400 – Please clarify why the pixel-level results differ from the site-level results. Is there a disproportionate

distribution of pixels to a small number of large sites that skews the analysis at the pixel level? Need to explain site level variation in efficacy… this is important both scientifically and from policy/practice standpoint, since “sites” are managed (not pixels). And if site size matters, then shouldn’t this be captured within the matching methods?? If not, justify why not… b/c size (or distance to boundary of area-based governance system?) is either an explanatory variable or a matching variable.

Other comments
5. Abstract: Talk about findings for extractive governance systems, too?
6. Introduction: highlight “conservation abandonment” generally as a challenge in conservation, as it affects all forms of conservation initiatives through both abdication of management responsibilities (e.g., paper parks) and rollbacks of formal protections (e.g., PADDD). See/cite new paper by Pienkowski et al on this. (Pienkowski, et al. Conservation abandonment is a policy blind spot. Nat Ecol Evol (2025). https://doi.org/10.1038/s41559-025-02910-5).
7. Probably makes sense to mention “paper parks” in this intro… and long-term financing for “permanence” in the discussion/conclusion.
8. Note that “reclassification” of PAs as OECMs is not PADDD. Reclassification is a change in conservation accounting and reporting; PADDD represents a substantive change in law/policy/regulations, with substantive impacts on the rules governing a site. (Reclassification may lead to PADDD, but they are procedurally and substantively different.)
9. Probably makes sense to cite the original paper on PADDD (Mascia & Pailler, 2011, Conservation Letters 4:1.)
10. Lines 353-364 – please report human pressure values for both sets of PAs (1-2 and 3-6) in the text.
11. Lines 372-373: Clarify please. Is this true for PAs in proportional or absolute terms or both?
12. Lines 382-84: Clarify please. Is there a difference or not (i.e., “similar”)? Seems like the manuscript is saying both “yes” and “no”.
13. 405-6: MCs were “ineffective”? or did they accelerate deforestation?
14. Table 2 – report PA results both aggregated (as shown) and disaggregated into the subcategories used elsewhere in the manuscript (e.g., Fig 1; I-II, III-VI).
15. Figure 2. Unclear what “darker colours” are – they all look the same. To make this point, show both “darker” and “lighter” in the figure legend.
16. Figure 3 panels A, B, and C are hard to read and interpret, since each panel includes multiple labeled Y axes. Suggest splitting fig 3 into 2 figures – one figure with panels a, b, and c vertically arrayed and a second figure with panels D and E.

(Remarks on code availability)

Reviewer #4

(Remarks to the Author)
Scientific research is relevant.
The proposed methodology is clear in each of its steps and provides sufficient detail for replicating the work in other areas of interest.
It presents its results adequately and illustrates its findings in the figures.
Its results are compared with recent research on the topic.
Regarding its conclusions, they highlight the importance of evaluating these governance mechanisms. Further research could be conducted on South America and how these mechanisms are effective in the Global South compared to other parts of the world.

(Remarks on code availability)

Reviewer #6

(Remarks to the Author)
The revised manuscript “Potential of different area-based governance mechanisms for achieving Global Biodiversity Framework goals” presents a global analysing of human footprint in protected areas and OECMs (as per WDPA database) and a region impact evaluation of multiple governance mechanisms avoided deforestation and carbon within the Peruvian Amazon. As a new reviewer I have largely tried to focus on whether the revisions successfully address the reviewer requests. The authors have addressed reviewer queries mostly but some further revisions are required in particular to address the substantive request around considering baseline trends before intervention dates. However, I did find that some of the new text introduced in response to reviewer 2 is problematic and ask for some further major revision to that text. I also note that I have major reservations around the inclusion of the global analysis and that my view is it detracts from the robust impact evaluation results. I encourage the authors to remove the analysis or move it solely to supplementary materials and to further address the reviewer 2 comments on the global results and consider my additional points. Lastly, I make line edit comments on the introduction which I found could be improved further. Please see my attached word document for my detailed comments to support this further revision.

(Remarks on code availability)

Version 3:

Reviewer comments:

Reviewer #6

(Remarks to the Author)
Thank you for your further revisions. You have addressed my substantive comments well and navigated at times competing reviewer demands. The manuscript is improved but retains it's core messages well. I did notice a few potential typographical errors that the authors may wish to attend to.
Line 87 - swapping between PAs (acronym) and spelled out protected areas - at times you swap between these or mix. Sentence on line 87 is a good example of this. Starts with "One third of PAs globally" and ends with "and 3% of forest within protected areas". I'd suggest trying to search text and clean up for consistency to make for a tighter read.
Line 96-101 - this new text reads well but doesn't flow (in my opinion) well in this paragraph. I'd suggest this text might work better perhaps at line 116 and with an additional bridging sentence to frame how you will address here an understanding of relative effectiveness of governance mechanisms (that might potentially be aligned with OECM criteria).
Line 100 - you introduce the term downgrading, downsizing, and degazettement (DDD) as one part of abandonment but then in discussion revert back to just referencing DDD. I find the use of DDD instead of full words very distracting. Although I am very familiar with PADDD, encountering DDD as an acronym within sentences made communication disjointed. Given your introduction defines abandonment and groups DDD within that definition I suggest in remainder of text replacing DDD with 'abandonment'.
Table s4 and Figure S1A - I found it hard to understand how these number match -given a much larger % of OECM area is in low pressure (~71%) relative to IUCN class III - VI (46%) I was expecting the box plots of visually have a clearer pattern of this density of points in lower class for OECMs and an equivalent or lower mean for OECM relative to multiple use PAs but Figure S1A seems to portray the opposite. Double check numbers and perhaps add some text in legends for clarity and include the mean HF values in Table S4 so a reader can at least verify across figures that the mean in the table matches the mean in the box plots.
Figure S4 - does a good job of complementing text methods. I did still find it hard to follow the volume of pixels in each of these stages kept or excluded and personally would have found it helpful to add that in so it's easier to then read results alongside this. However, as a methods workflow this figure does the job needed

(Remarks on code availability)

Open Access This Peer Review File is licensed under a Creative Commons Attribution 4.0 International License, which permits use, sharing, adaptation, distribution and reproduction in any medium or format, as long as you give appropriate credit to the original author(s) and the source, provide a link to the Creative Commons license, and indicate if changes were made.
In cases where reviewers are anonymous, credit should be given to 'Anonymous Referee' and the source.
The images or other third party material in this Peer Review File are included in the article’s Creative Commons license, unless indicated otherwise in a credit line to the material. If material is not included in the article’s Creative Commons license and your intended use is not permitted by statutory regulation or exceeds the permitted use, you will need to obtain permission directly from the copyright holder.

To view a copy of this license, visit https://creativecommons.org/licenses/by/4.0/

Response to reviewers' comments

Thank you for the opportunity to respond to the valuable feedback received. We have responded in **bold** to all the comments.

Reviewer #1 (Remarks to the Author):

This is an interesting ms but one which still needs a deal of work. Detailed comments are below, but overall, the ms could do with reframing and sharpening the arguments. More needs to be explained as well as to how a geographically narrow focus could be upscaled.

We thank the reviewer for their feedback and suggestions. We have now modified the manuscript extensively to include the reviewer comments and suggestions. We have now included a new analysis at the global level describing the extent and intensity of human pressure within territories already designated as OECMs. We have also modified the abstracts, introduction and discussion sections to provide a more global view of all the results and to contextualize how these types of assessments can be standardized and replicated in other countries to identify and integrate OECMs more effectively into national and global conservation planning. Specifically, we modified the abstract to discuss the Peruvian Amazon case study results in the light of the global analysis. Now the abstract reads;

"The Kunming-Montreal Global Biodiversity Framework (GBF) includes a target for protecting 30% of land by 2030 and refers to other effective area-based conservation measures (OECMs) as complementary to Protected Areas (PAs) for this target. To explore their utility for conservation, here we investigated the level of human pressure across the global OECM network. We also assessed the impact of different governance mechanisms with potential to be listed as OECMs - Indigenous Lands (ILs), Non-Timber Forest products Concessions (NTCs) and Logging Concessions (LCs) - as well as PAs on forest loss and its associated carbon emissions in the Peruvian Amazon from 2005 to 2021. We used a robust control intervention study design, with statistical matching, to account for the non-random spatial distribution of deforestation pressure and the governance mechanisms analysed. Globally, our results show that OECMs generally have higher human pressure levels than strictly protected PAs, but similar pressure levels to less strictly protected PAs. At the Peruvian Amazon level, we show that PAs were highly effective at preventing forest loss (88% avoided), but that NTCs and ILs were also effective (66% and 42%, respectively). Our study provides evidence of the complementarity of OECMs to PAs for biodiversity protection and of the long-term positive impacts of different governance mechanisms at preventing forest loss and reducing carbon emission. This information is key to advance in the identification and declaration of OECMs and to more effectively achieve global environmental goals."

We have also included a section in the introduction on lines 83 to 87 describing the state of OECMs globally and the need to assess the condition and quality of land within territories already designated as OECMs;

"More than 1.9 million km² of the Earth's surface has already been reported to the World Database on OECMs [1], representing approximately 1% of terrestrial areas (Fig. 1). Two thirds of this land is governed by federal or national agencies [2]. With OECMs rapidly gaining

attention and traction in conservation policy, a clear understanding on the effectiveness of various OECM designations as conservation mechanisms is needed [2].”

Additionally, we have modified some sections of the introduction to showcase previous work on different governance mechanisms and their contribution to biodiversity conservation in the light of global patterns of OECM coverage. For example, in line 88 to 92 for the description of ILs;

“There are a wide range of governance mechanisms currently recognised as OECMs [2,3]. One of those is Indigenous Lands (ILs). There is growing evidence showing that ILs can be effective at reducing deforestation under certain management conditions [4–7]. For example, a study in the Brazilian Amazon found that deforestation and burned areas inside ILs were two and four times lower than outside, respectively [8].”

Also, we modified some sections of the Discussion to describe the results of the Peruvian Amazon in the light of the global trends on OECM coverage. Including in lines 466 to 482;

“The GBF includes a target of 30% of land protected by 2030 and refers to OECMs as a complementary conservation approach to PAs [9]. Our study highlights the importance of PAs and OECMs as tools for achieving global climate and biodiversity goals. At the global level we show that OECMs have higher human pressure levels than PAs, but much lower levels than unprotected land, and that many individual OECMs have very low human pressure levels. Our analysis at the level of the Peruvian Amazon provides robust evidence of long-term conservation impacts in both PAs and other governance mechanisms that have the potential to be listed as OECMs. While our results show that PAs are the most effective mechanism for preventing forest loss and carbon emissions, we also found that there are several other governance mechanisms that can deliver conservation benefits at different extents and with different management characteristics. These types of analysis could be replicated in other countries and landscapes to identify governance mechanisms that can be listed as OECMs. The creation of a unified framework on how to conduct these assessments under different conditions would facilitate such process in a consistent manner. To allow nations to more effectively achieve the different targets from the GBF and the UN Framework Convention on Climate Change [10], it will be crucial to (i) better understand how different types of governance mechanisms operate and under which circumstances they can provide the most beneficial conservation outcomes, and (ii) strengthen their management through funding mechanisms specific to each governance mechanism.”

In Abstract: Introduce acronyms in full first e.g. PAs

We thank the reviewer for pointing this out. We have now modified the abstract accordingly.

L46 if Pas only avoid 88% rather than the expected 100%, how is forest lost in a PA? what is the problem here?

As the reviewer highlights, PAs avoided 88% of the deforestation that would have been expected. No PA is 100% effective, and so some deforestation is always expected. But, a

value of 88% is high and consistent with other studies at regional and global level. We discuss this more in depth in the introduction in lines 63 to 69;

"Protected areas (PAs) are a critical tool for the protection of forests and the ecosystem services they provide. There is strong evidence that PAs reduce deforestation and forest degradation pantropically [11], and more than 18% of global land is now under some kind of protection regime [1,12]. Despite their expanding coverage and success, PAs also face many challenges. One third of PAs globally are under intense human pressure [13], more than half of protected forests have a substantial degree of anthropogenic disturbance [14], and 3% of the world's protected forest was lost in the first decade of the 20th century [15]."

L50 – they are actual, not potential. And what other mechanisms?

We use the term "potential" here, as these areas are existing governance mechanisms which meet many of the criteria to be defined as OECMs, but that in many instances (including our Peruvian Amazon case study) have not been officially listed as OECMs. We agree that this wording was unclear, so we have now modified the paragraph to provide more clarity. The paragraph now reads;

"Our study provides evidence of the complementarity of OECMs to PAs for biodiversity protection and of the long-term positive impacts of different governance mechanisms at preventing forest loss and reducing carbon emission. This information is key to advance in the identification and declaration of OECMs and to more effectively achieve global environmental goals."

We also discuss different governance mechanisms present in the Peruvian Amazon, their characteristics and their potential to be designated as OECMs in the introduction in lines 86 to 103. We have also added a paragraph in the introduction in lines 100 to 103 discussing other potential governance mechanisms that have been associated with positive biodiversity outcomes and with the potential to be designated as OECMs:

"Other governance mechanisms that have been associated with positive conservation outcomes and that could be listed as OECMs include sacred sites, military areas, private, urban and surfing reserves and university research areas among others [3]."

L60 Tropical forests do have much species richness, not just in vertebrates. It is also true they store carbon, but increasing work shows wetlands (even ones in temperate zones) are the most effective ecosystems at sequestering carbon.

We agree with the reviewer in that tropical forests have a high overall species richness, not just vertebrates. That is why we refer to biodiversity in the paragraph and not vertebrate species alone. We also agree with the reviewer that other types of ecosystems including wetlands play key roles in carbon sequestration. In order to account for this in that paragraph we now do not make comparisons with other ecosystems and instead just

highlight the importance of tropical forest as a key ecosystem in terms of biodiversity and carbon storage. The paragraph now reads;

"Tropical forests are among the world's most biodiverse ecosystems [16,17]. They also provide crucial services to humanity [18,19], including storing and sequestering large amounts of atmospheric carbon, which is key to directly regulating climate [20]. Despite this, the loss and degradation of tropical forests has increased over time [21,22], contributing up to 20% of the world's greenhouse gas emissions [20,23]."

L66 the figure is closer to 18%.

We thank the reviewer for pointing this out. We have now corrected the figure to 18% and updated the citations to;

- **UNEP-WCMC, and IUCN. "Protected Areas (WDPA)." Protected Planet, 2025. https://www.protectedplanet.net/en/thematic-areas/wdpa?tab=About.**
- **UNEP-WCMC, and IUCN. "World Database on Other Effective Area-Based Conservation Measures (WD-OCEM)." Protected Planet, 2025. https://www.protectedplanet.net/en/thematic-areas/oecms?tab=OECMs.**

L67 if Pas are not effective then maybe they are not working? What does low or medium integrity mean?

While PAs are not 100% effective, they do reduce deforestation and biodiversity loss. Moreover, they are regarded as a fundamental tool for the protection of forest ecosystems. We have now included a paragraph in lines 63 to 65 to highlight this fact;

"Protected areas (PAs) are a critical tool for the protection of forests and the ecosystem services they provide. There is strong evidence that PAs reduce deforestation and forest degradation pantropically [11], and more than 18% of global land is now under some kind of protection regime [1,12]."

We agree with the reviewer that low or medium integrity was unclear. We were referring to forest integrity categories from Grantham et al. (2020), but have now changed the sentence for clarity;

"One third of PAs globally are under intense human pressure [13], more than half of protected forests have a substantial degree of anthropogenic disturbance [14], and 3% of the world's protected forest was lost in the first decade of the 20th century [15]."

L75: What type of socioeconomic conflicts?

We thank the reviewer for pointing this out. We have now included some explicit examples of social conflicts to contextualize. The paragraph now reads;

"Additionally, the establishment of PAs has sometimes been associated with negative socioeconomic outcomes including displacement of local communities and restriction of access to local resources [24,25]. Moreover, in some circumstances, the designation of a PA might not be appropriate or feasible, so other governance mechanisms may be more effective in delivering positive conservation outcomes [26]."

L80 - 82: This is great, but some examples could be helpful.

We thank to the reviewer for this suggestion. We have now added an example in lines 89 to 92 to better explain the point. The new paragraph reads;

"There is growing evidence showing that ILs can be effective at reducing deforestation under certain management conditions [4–7]. For example, a study in the Brazilian Amazon found that deforestation and burned areas inside ILs were two and four times lower than outside, respectively [8]."

L80 perhaps cite the ipbes global assessment (2019) which has detail on this.

We thank the reviewer for pointing out this report. We have now included it as a citation in this paragraph.

L104 seems to repeat some of the discussion above.

We thank the reviewer for pointing this out. We have now removed reference to the non randomly distribution of PAs as this was mentioned in the previous paragraph. Now the paragraph reads;

"Despite their growing importance, rigorous evaluations of the effectiveness of potential OECMs and other alternative governance mechanisms in preventing forest loss, particularly relative to PAs, remain relatively sparse [27–30]. Many assessments fail to account for the non-random location of PAs and OECMs. Some studies, for example, compare areas that are protected to unprotected land in the study area [15,31], or to neighbouring buffers [32]. This can cause bias in the estimated impact of PAs as they tend to be located in places that are less likely to be cleared [26,33,34]. As countries continue to designate PAs and report OECMs in pursuit of the goals and targets in the UN Framework Convention on Climate Change [10] and the GBF, it is crucial to understand their contributions and complementarity to achieve the best conservation and climatic outcomes [2,28]."

L111 conservation and carbon surely?

We thank the reviewer for pointing out the lack of reference to carbon in this paragraph. We have now modified the paragraph to include a mention to climate as an indirect reference to the impact of carbon emission in climate change. The paragraph now reads;

"As countries continue to designate PAs and report OECMs in pursuit of the goals and targets in the UN Framework Convention on Climate Change [10] and the GBF, it is crucial to understand their contributions and complementarity to achieve the best conservation and climatic outcomes [2,28]. "

L 156 OECMs are defined by the CBD in Decision 14/8 as "a geographically defined area other than a Protected Area, which is governed and managed in ways that achieve positive and sustained long-term outcomes for the in-situ conservation of biodiversity, with associated ecosystem functions and services and where applicable, cultural, spiritual, socio–economic, and other locally relevant values". This is the reference that is preferable to use.

We thank the reviewer for this clarification. We have now added this citation to the paragraph. And provided a full definition of OECMs in the introduction in lines 74 to 85;

"It is now clear that PAs alone are unlikely to be sufficient for meeting the targets of the Kunming-Montreal Global Biodiversity Framework (GBF) [9], particularly target 3, which calls for protection of 30% of the planet by 2030 [9]. In response, much attention is now being placed on other effective area-based conservation measures (OECMs) that can deliver positive conservation outcomes to complement those provided by PAs [25,35]. For a governance mechanism to be listed as an OECM, it cannot be already classified as a PA, and must be a geographically defined area, which is governed and managed in ways that achieve positive and sustained long-term outcomes for the in-situ conservation of biodiversity [36–38]. More than 1.9 million km² of the Earth's surface has already been reported to the World Database on OECMs [1], representing approximately 1% of terrestrial areas (Fig. 1). Two thirds of this land is governed by federal or national agencies [2]. With OECMs rapidly gaining attention and traction in conservation policy, a clear understanding on the effectiveness of various OECM designations as conservation mechanisms is needed [2]."

L162 – such sites as you mention may have long-term potential for returning positive biodiversity and Carbon results?

We agree with the reviewer in that this aspect should be included in the text. We have now modified the paragraph to include this sentiment. The paragraph now reads;

"LCs, on the other hand, were included in a separate group as their main objective is commercial extraction of wood. While these concessions may have long-term potential for returning positive biodiversity and carbon outcomes, their overarching objective does not align with criterion six required for their identification [37] and criterion C required for their recognition and reporting as OECMs [36]. These two criteria specify that sites that are subject to environmentally damaging industrial activities cannot be listed as OECMs."

L173: How was the accuracy assessment conducted?

We have now included a brief description of the validation procedure and a citation to the full description of the method in lines 216 to 219. Now the paragraph reads;

"The accuracy of these maps was calculated by randomly selecting 3000 sample pixels and contrasting the estimated transitions from 2000 to 2011 (no change or forest loss) with visual observation of high resolution satellite images [39]. The estimated accuracy was 92.2% for forest loss and 99.5% for no change [39,40]."

L177 et sq this sems a very rough way to deal with Carbon…

We agree with the reviewer that these are estimated carbon densities which are not 100% accurate. However, the estimations are the most accurate available for Peru. Other products available are calculated at the continental or global level and thus have high uncertainty levels for use in a national scale analysis. The developers of the carbon estimations tested the accuracy of their estimations through a validation process. We have now provided more detail on how the carbon estimates were calculated and how the validation process was done as well as the accuracy of the estimations. The paragraph now reads;

"For carbon, we used high-resolution above ground carbon density estimations for Peru, generated by Asner et al. (2014) [41], to calculate metric tons (Mg) of carbon by forest pixel. These carbon estimations integrate countrywide ecosystem sampling using airborne light detection and ranging (LiDAR) with high-resolution satellite imaging [41]. Previous studies using LiDAR to estimate carbon density have found a precision and accuracy similar to estimates based on field measurements [42]. The carbon estimations in Asner et al. (2014) [41] had an agreement of 74% with field based carbon estimations from 51 vegetation plots in the study area."

L179 – it would be important to understand the extent of soil carbon that is left after clearing. Maybe it is extremely low, but it should be mentioned.

We agree with the reviewer that it is important to acknowledge that below ground carbon is not included in these estimations and how much is the proportion of such biomass. We have now included a paragraph in lines 233 to 236 mentioning this;

"In this study, "carbon emissions" refers to emissions "committed" at the time of disturbance or clearing, noting that there may be a time lag until they are "realized". Below ground carbon is not included in these estimations but has been estimated to account for an average 22% in forest ecosystems [43]."

L185 – I am unfamiliar with cofounding factors – should it be confounding?

We thank the reviewer for pointing this out. We have now changed the word to "confounding".

L198 – the statistical matching process seems appropriate but there are doubts as to the robustness of data being analysed based on the earlier descriptions.

As suggested by the reviewer we have now included more detail on the methodology used to generate the input data for the analysis, as well as the validation process and the accuracy of the datasets. This includes additional information on the validation process and accuracy of the forest cover dataset in line 216 to 221;

"The classification of a pixel into either forest or no forest is based on different Landsat images of the Peruvian Amazon for each year, which are then processed at a pixel level. The accuracy of these maps was calculated by randomly selecting 3000 sample pixels and contrasting the estimated transitions from 2000 to 2011 (no change or forest loss) with visual observation of high resolution satellite images [39]. The estimated accuracy was 92.2% for forest loss and 99.5% for no change [39,40]."

Information on the validation process and accuracy and limitations of the above ground carbon per hectare density estimates was also added to lines 224 to 236;

"For carbon, we used high-resolution above ground carbon density estimations for Peru, generated by Asner et al. (2014) [41], to calculate metric tons (Mg) of carbon by forest pixel. These carbon estimations integrate countrywide ecosystem sampling using airborne light detection and ranging (LiDAR) with high-resolution satellite imaging [41]. Previous studies using LiDAR to estimate carbon density have found a precision and accuracy similar to estimates based on field measurements [42]. The carbon estimations in Asner et al. (2014) [41] had an agreement of 74% with field based carbon estimations from 51 vegetation plots in the study area. We assumed that if a pixel was deforested, all its carbon was emitted into the atmosphere. Then we summed the amount and proportion of carbon emissions by year and cumulative between 2005 and 2021 for the whole landscape, and for each governance mechanism as well as for the selected control and treatment pixels after the matching analysis. In this study, "carbon emissions" refers to emissions "committed" at the time of disturbance or clearing, noting that there may be a time lag until they are "realized". Below ground carbon is not included in these estimations but has been estimated to account for an average 22% in forest ecosystems [43]."

L216 should be were, not where

We thank the reviewer for pointing out this grammar mistake. We have now corrected it.

L251 – it is interesting that PAs showed any loss at all – I think more could be made of this. See l67 comments

We thank the reviewer for pointing this out. There is strong scientific evidence showing that there is widespread human pressure inside PAs[44] and that PAs are not 100% effective preventing deforestation globally [6] and regionally [45,46]. Our results align with these previous findings. We discuss this in the introduction on lines 63 to 69;

"Protected areas (PAs) are a critical tool for the protection of forests and the ecosystem services they provide. There is strong evidence that PAs reduce deforestation and forest degradation pantropically [11], and more than 18% of global land is now under some kind of protection regime [1,12]. Despite their expanding coverage and success, PAs also face many

challenges. One third of PAs globally are under intense human pressure [13], more than half of protected forests have a substantial degree of anthropogenic disturbance [14], and 3% of the world's protected forest was lost in the first decade of the 20th century [15]."

We also mention this in the discussion on lines 382 to 388;

"In our analysis of the Peruvian Amazon we found that 3.5% of 2005 forest extent was lost between 2005-2021. Despite this widespread loss, PAs and other governance mechanisms have played a significant role in avoiding even greater declines in forest cover. We showed that PAs experienced forest loss rates eight times lower than similar areas without protection, with smaller but still substantial effects for NTCs and ILs (approximately 3x and 2x reductions, respectively). PAs, ILs and NTCs cover 17.4, 20 and 1.3% of the Peruvian Amazon's area, respectively, and their impact on reducing deforestation in the region is remarkable."

L283 et seq – these are interesting findings! I think more explanation would be warranted.

We thank the reviewer for pointing this out. We agree that these results are interesting, particularly the fact that the lowest median carbon density was for Mining Concessions. Despite this, these differences were not statistically significant. Due to the lack of statistical significance in these differences and space limitations constrains by the journal we have maintained the length of this section as it is now.

L300 this does not chime with the statements in 283 noted above or am I missing something?

The paragraph in line 283 in the results section describes the median carbon density of all the pixels inside each governance mechanism assessed. The paragraph in line 300 in the discussion section talks about the effectiveness of PAs and the other governance mechanisms assessed preventing forest loss. While these are topics that are related to some extent to one another, we discuss them separately, precisely to avoid confusion.

L314 – conservation funding... this is a complex topic – what funding is foreseen here? National? International? And it is surely positive conservation and carbon outcomes that are sought?

We have now clarified that the expected outcomes are not only in relation to biodiversity conservation but also to carbon sequestration. We also expanded the paragraph to include potential sources of funding as requested by the reviewer. The paragraph now reads;

"The contribution of these governance mechanisms to regional and global conservation and climate change mitigation goals should be quantified and acknowledged, especially in economic terms. Conservation funding can come from different sources including government programs and carbon and biodiversity markets [47]. This funding could then be channelled to maintain or increase the contributions that these governance mechanisms

provide to biodiversity conservation and carbon sequestration as well as to better understand under which circumstances they provide the best conservation outcomes."

L322 this seems a little confused – is the idea for carbon credits< or direct funding from GEF/WB?

We agree with the reviewer that this paragraph needed more clarity. What we wanted to highlight in this paragraph was the importance of estimating avoided carbon emissions for governance mechanisms to access potential funding sources. We have now modified the paragraph to make this sentiment clearer. The paragraph now reads;

"The estimation of the amount of carbon emissions avoided is a key measure to understand the role of these governance mechanisms in carbon cycles and regional climate regulation [35,48]. Additionally, these estimations are required to access REDD+ and other funding streams (e.g., from the Global Environmental Facility and the World Bank) [49,50]. Our results show that OECMs will be essential tools for achieving global climate and biodiversity goals."

L331-37 – this seems at variance with the lines above? And rather confused argumentation.

We agree with the reviewer that this paragraph was not clear. Now we have reformulated the section and removed some text to make it clearer. Now the paragraph reads;

"From 2005 to 2021, ILs avoided 42% of the forest loss and 41% of the carbon emissions that would be expected if those areas were unprotected. These results show that ILs can provide important conservation benefits in terms of preventing forest loss and reducing carbon emissions and our findings align with previous research conducted in the Amazon [5,29,51] and at global level [6]. While the effectiveness of ILs preventing deforestation and carbon emission is lower than that of PAs, they currently cover 20% of the Peruvian Amazon. It is thus key to support Indigenous Communities in co-designing and accessing financial mechanisms that would allow them to receive forest-based income through activities that do not harm biodiversity. It is also crucial to support these Indigenous Communities in the monitoring and control of unsustainable extractive activities like mining, or logging, and of illegal activities like cultivation of illicit crop, which are harmful to biodiversity [52–54]."

L341 – here the paper moves into the delicate territory of ILK, and the need for FPIC. I would suggest this reformulated or even omitted.

While FPIC is fundamental, here we are not referring specifically to the environmental and forestry policies inside their lands but to a broader regional, national and global level, and how ILK and the participation of indigenous people in environmental and forestry policy-making is key to achieve better conservation outcomes. We have now modified the paragraph to make this point clearer. Now it reads;

"The influence that Indigenous territorial governance and management has on the regional trends of deforestation and carbon emissions is substantial. In an environmental justice

logic, it is therefore also crucial to support the claims by Indigenous Peoples in the Amazon and globally, who frequently advocate for better recognition of their contribution to global conservation goals [6,52]*. Acknowledging their contribution through the opening of spaces for their direct participation in environmental and forestry policy-making and through the provision of funding to support their manifold approaches to land governance is necessary. Also, the identification of the specific management practices that are effective in preventing deforestation is crucial, so that they can be scaled to other comparable contexts in the Amazon and beyond."*

L351 et seq. Yes, these conclusions are significant. The problem with OECM is that it is difficult to see where they end…

While it is true that the future of OECMs at this stage is uncertain, in this particular section we are referring to LCs, and how even though they can provide positive outcomes for biodiversity they do not meet all the criteria for being designated as OECMs.

Specifically in relation to LCs, we are also cautious on their future potential for providing positive outcomes for biodiversity. We discuss this in lines 437 to 439 of the discussion section;

"Despite the potential benefits of well-managed LCs in comparison to other extractive activities, it is still important to note that any logging has a negative impact on tropical forest ecosystems, as the extraction of trees degrades the forest [55,56]***."***

And in lines 445 to 450;

"While there is evidence that the effectiveness of LCs in preventing forest loss is higher in areas with higher deforestation pressure [57]***, more studies on LCs are needed to understand what factors and which management conditions help reducing forest loss. Furthermore, a better understanding of how the extraction of wood affects the overall biodiversity and carbon density in such concessions is a priority*** [55,56,58]***."***

L357 – here it depends on intensity. Limited and targeted logging may have positive effects.

We agree with the reviewer on this, and our results point out to an overall positive effect of LCs avoiding deforestation. We mention this in lines 427 to 428;

"Further, we found that LCs avoided 24% of the deforestation and 18% of the expected carbon emissions between 2005 and 2021, compared to similar unmanaged areas."

Despite this we prefer to be cautious and also mention the negative impacts that LCs have on forest conservation. Specifically in lines 437 to 439;

"Despite the potential benefits of well-managed LCs in comparison to other extractive activities, it is still important to note that any logging has a negative impact on tropical forest ecosystems, as the extraction of trees degrades the forest [55,56]***."***

And lines 445 to 450;

"While there is evidence that the effectiveness of LCs in preventing forest loss is higher in areas with higher deforestation pressure [57], more studies on LCs are needed to understand what factors and which management conditions help reducing forest loss. Furthermore, a better understanding of how the extraction of wood affects the overall biodiversity and carbon density in such concessions is a priority [55,56,58]."

L375-76 this needs reformulating previous, and posterior appear to mean the same?

We thank the reviewer for pointing out the redundancy of this paragraph. Due to the request from the editor and reviewers to add other sections to the document and space limitations constrains by the journal we have now deleted this section as it was redundant.

L385 this may not be universally true… disturbed forest can have dense regrowth.

We thank the reviewer for pointing this out. Due to the request from the editor and reviewers to add other sections to the document and space limitations constrains by the journal we have now deleted this section.

L392 – this is a bold claim that may not be accurate.

We agree with the reviewer and now have reworded the sentence. Now it reads;

"The GBF includes a target of 30% of land protected by 2030 and refers to OECMs as a complementary conservation approach to PAs [9]. Our study highlights the importance of PAs and OECMs as tools for achieving global climate and biodiversity goals. At the global level we show that OECMs have higher human pressure levels than PAs, but much lower levels than unprotected land, and that many individual OECMs have very low human pressure levels. Our analysis at the level of the Peruvian Amazon provide robust evidence of long-term conservation impacts in both PAs and other governance mechanisms that have the potential to be listed as OECMs."

Reviewer #2 (Remarks to the Author):

Major comments

1. This manuscript addresses an important topic – the relative impact on deforestation rates and carbon emissions of different land management regimes. The topic has been studied by others (see Lambin, Blackman, etc.), but scientific understanding remains in its infancy.

We thank the reviewers for highlighting the novelty of this research and the overall positive feedback

2. The manuscript does not address *survivorship bias* in its sample of governance systems. That is, the sample does not appear to include interventions that were established and subsequently abandoned or revoked. If not, then the study likely overstates the impact of these interventions (e.g., protected areas) because it examines the impacts of ONLY those sites that persisted throughout the study period – and excludes those that did not "survive" until 2021 (the end of the study period). At the least, this should be acknowledged as a limitation of the study. See the robust literature on PADDD (Protected Area Downgrading, Downsizing, and Degazettement) by Mascia and others, including Mascia and Pailler (2010), Forrest et al (2014), Pack et al (2016), Tesfaw et al (2018), Golden Kroner et al (2019), and Keles et al (2020, 2023) and the PADDD data for Peru (available via PADDDtracker.org). We also know that forest certification/concessions, IL rights, and other forms of governance studied here can be revoked or modified.

We thank the reviewer for this comment and for the suggested literature on PADDD events. We agree that PADDD events and their impact on the effectiveness of conservation areas at preventing deforestation are an important aspect to take into account. However, in the particular case of the Peruvian Amazon there were no PADDD events for PAs [59] or ILs during the time period of the analysis. In the case of ILs, once they are designated they acquire this status in perpetuity[60,61]. In relation to concessions, we exclude the ones that became not operational before 2015, this only affected MCs as LCs and NTCs didn't have any case of concessions closed before 2015. We have now added a paragraph in the methodology in lines 253 to 257 to clarify these aspects;

"Also, forest concessions that stopped operating before 2015 were excluded. This only impacted MCs. There were no LCs or NTCs that stopped operating before 2015 in our dataset. There were no PA Downgrading, Downsizing, and Degazettement (PADDD) events recorded between 2005 and 2021 [59,62], so no PAs were excluded from the analysis based on this. For ILs, once designated they acquire this status in perpetuity [60,61], so again there were no sites to be removed."

Additionally, in order to acknowledge the potential effect of *survivorship bias* we now included a section in the discussion in lines 458 to 462, where we discuss the implications of downgrading, downsizing and degazetting events on PAs and on other governance mechanisms with potential to be listed as OECMs, and the impact this could have on biodiversity outcomes. In this paragraph we include most of the citation suggested by the reviewer;

"Second, while there were no PADDD events in the Peruvian Amazon during the time of our analysis, there is evidence that shows that these events are associated with increased deforestation [63] , human pressure [64], and ineffective protection [65]. It is thus crucial to improve the understanding of how PADDD events may impact the achievement of the GBF goals [9], and to assess the impacts of PADDD on OECMS and potential OECMS globally."

3. More generally, the temporal elements of the study are not clear. Is the conservation impact measured at t+1 (where t = date of establishment for the protected area, etc) or assessed based on the full time period of the ecological data (2000-2001)? Baseline is presumably pre-

establishment year or years, with impact as post-establishment, but it isn't clear that the data are analyzed in this way. At the least, clarification is necessary; if the impact isn't estimated on purely post-establishment years, then modifications to the analyses are required. This comment is relevant to all treatments/governance systems studied.

We thank the reviewer for pointing out the lack of clarity in these aspects in the manuscript. While we had information on forest cover loss between 2000 and 2021, many governance mechanisms were stablished between 2000 and 2005, so we decided to include all of the ones that had been established before 2005 in order to maximise the total area of different governance mechanisms we could assess. Due to this, initially we did two matching analyses, one using deforestation data between 2000 to 2021 and another one using deforestation data between 2005 and 2021. We explained this in lines 199 to 201 of the original manuscript and the results of the matching analysis using deforestation between 2005 and 2021 are shown in the supplementary material;

"We used forest loss between two time periods as alternative outcome variables for our matching analysis in order to assess the robustness of our results. Forest loss by pixel between 2000 and 2021 and forest loss by pixel between 2005 and 2021."

Both matching analyses provided similar results, so we showed the results of the analysis using deforestation between 2000 and 2021 in the main text and the results using deforestation between 2005 and 2021 in the supplementary information. The main difference is that for the analysis between 2005 and 2021, the difference between LCs and counterfactual pixels is not significant. In order to improve clarity, we have now only included the analysis for the 2005 to 2021 period. We have also now adapted the manuscript and the figures accordingly and removed the results for the matching analysis using deforestation between 2000 and 2021 to avoid confusion.

Particularly in relation to the method section in lines 252 related to the time frame used in the matching analysis. It now reads;

"We used forest loss between 2005 and 2021 as the outcome variable for our matching analysis."

4. The manuscript would be stronger if it clarified what residual forms of governance are considered as potential control sites. Given all of the governance systems that are considered treatments, it isn't clear what other forms of governance exist in the Peruvian Amazon.

We agree with the reviewer in this comment. While it is challenging to account for all the potential governance mechanisms present in the study area, here we endeavoured to include the most widespread types during the time frame of the analysis. Additionally, some of the governance mechanisms that we didn't include have not been systematically mapped which makes it challenging to account for them. We have now included a new caveat in the discussion in lines 451 to 458 where we have clarified this;

"The results of our analysis comparing the effectiveness of potential OECMs preventing

forest loss in the Peruvian Amazon provides an overview of the impact of these governance mechanisms avoiding deforestation and its associated carbon emissions, but there are some caveats that must be accounted for. First, for this analysis we assessed the most widely distributed governance mechanisms in the Peruvian Amazon, but there are other governance mechanisms with potential to be listed as OECMs, including private [66] *and regional*[67] *conservation areas, that we could not assess. It would be valuable to evaluate their impact on biodiversity conservation in future studies and assess their potential to be listed as OECMs."*

5. It would strengthen the manuscript if the discussion looked beyond Peru to consider the scientific and policy implications for tropical forests generally. Given the title focuses on a global question, the manuscript (including the discussion) should speak to the global.

We agree with the reviewer that the manuscript needed a broader perspective and a better discussion on how the Peruvian Amazon analysis and results could be upscaled. We have now included a new analysis at the global level describing the extent and intensity of human pressure within territories already designated as OECMs. We have also modified the abstracts, introduction and discussion sections to provide a more global view of all the results and to contextualize how these types of assessments can be standardized and replicated in other countries to identify and integrate OECMs more effectively into national and global conservation planning. Specifically, we modified the abstract to discuss the Peruvian Amazon case study results in the light of the new global analysis. Now the abstract reads;

"The Kunming-Montreal Global Biodiversity Framework (GBF) includes a target for protecting 30% of land by 2030 and refers to other effective area-based conservation measures (OECMs) as complementary to Protected Areas (PAs) for this target. To explore their utility for conservation, here we investigated the level of human pressure across the global OECM network. We also assessed the impact of different governance mechanisms with potential to be listed as OECMs - Indigenous Lands (ILs), Non-Timber Forest products Concessions (NTCs) and Logging Concessions (LCs) - as well as PAs on forest loss and its associated carbon emissions in the Peruvian Amazon from 2005 to 2021. We used a robust control intervention study design, with statistical matching, to account for the non-random spatial distribution of deforestation pressure and the governance mechanisms analysed. Globally, our results show that OECMs generally have higher human pressure levels than strictly protected PAs, but similar pressure levels to less strictly protected PAs. At the Peruvian Amazon level, we show that PAs were highly effective at preventing forest loss (88% avoided), but that NTCs and ILs were also effective (66% and 42%, respectively). Our study provides evidence of the complementarity of OECMs to PAs for biodiversity protection and of the long-term positive impacts of different governance mechanisms at preventing forest loss and reducing carbon emission. This information is key to advance in the identification and declaration of OECMs and to more effectively achieve global environmental goals."

We also now included a section in the introduction in lines 81 to 85 describing the characteristics of OECMs globally and the need to assess their effectiveness preventing biodiversity loss;

"More than 1.9 million km² of the Earth's surface has already been reported to the World Database on OECMs [1], representing approximately 1% of terrestrial areas (Fig. 1). Two thirds of this land is governed by federal or national agencies [2]. With OECMs rapidly gaining attention and traction in conservation policy, a clear understanding on the effectiveness of various OECM designations as conservation mechanisms is needed [2]."

We have modified some sections of the introduction to showcase previous work on different governance mechanisms and their contribution to biodiversity conservation in the light of global patterns of OECM coverage. For example, in line 86 to 88 for the description of ILs;

"There are a wide range of governance mechanisms currently recognised as OECMs [2,3]. One of those is Indigenous Lands (ILs). There is growing evidence showing that ILs can be effective at reducing deforestation under certain management conditions [4–7]."

Also we modified some sections of the Discussion to describe the results at the Peruvian Amazon level in the light of the global trends on OECMs coverage. Including in lines 470 to 486;

"The GBF includes a target of 30% of land protected by 2030 and refers to OECMs as a complementary conservation approach to PAs [9]. Our study highlights the importance of PAs and OECMs as tools for achieving global climate and biodiversity goals. At the global level we show that OECMs have higher human pressure levels than PAs, but much lower levels than unprotected land, and that many individual OECMs have very low human pressure levels. Our analysis at the level of the Peruvian Amazon provide robust evidence of long-term conservation impacts in both PAs and other governance mechanisms that have the potential to be listed as OECMs. While our results show that PAs are the most effective mechanism for preventing forest loss and carbon emissions, we also found that there are several other governance mechanisms that can deliver conservation benefits at different extents and with different management characteristics. These types of analysis could be replicated in other countries and landscapes to identify governance mechanisms that can be listed as OECMs. The creation of a unified framework on how to conduct these assessments under different conditions would facilitate such process in a consistent manner. To allow nations to more effectively achieve the different targets from the GBF and the UN Framework Convention on Climate Change [10], it will be crucial to (i) better understand how different types of governance mechanisms operate and under which circumstances they can provide the most beneficial conservation outcomes, and (ii) strengthen their management through funding mechanisms specific to each governance mechanism."

<u>Other comments</u>

6. Note that Lambin et al have examined the impact of "stacked" interventions. At the least, this is worth mentioning. "stacked interventions" may also merit inclusion in this analysis as a form of governance.

We thank the reviewer for raising this point and for providing the Lambin article information. In Peru, cases of overlapping allocations are mostly unintentional and

commonly due to lack of coordination between different government bodies, so we did not include them as an additional governance category. We have now included a new paragraph in the discussion in lines 462 to 469 where we discuss the implication of overlapping land tenures. We have also included the citation suggested by the reviewer in this paragraph;

"Third, while for our analysis we excluded cases where there was an overlap between different governance mechanisms, it is important to highlight that previous studies have found that in areas of overlapping land governance there is lower deforestation [68]. Cases of overlapping land allocations in Peru are mostly unintentional and commonly due to lack of coordination between different government bodies. This problem shows the need for an unified cadastre in the country [69]. More broadly, there is a need for a better understanding of the causes and consequences of overlapping land governance for biodiversity conservation [68]."

7. Note the work of Pfaff and others on the impacts of FSC certification on deforestation in Peru and elsewhere.

We agree with the reviewer on the relevance of Pfaff work for this manuscript. We discuss this topic in lines 427 to 434 and we do reference Pfaff work in Peru and other similar analysis in Borneo and Cameroon;

"Further, we found that LCs avoided 24% of the deforestation and 18% of the expected carbon emissions between 2005 and 2021, compared to similar unmanaged areas. While these results were not statistically significant and LCs do not meet all the criteria to be recognized as OECMs, these concessions do constitute a considerable proportion of the Peruvian Amazon (8.1%), and so they can still make a significant contribution to other conservation goals like target 10 of the GBF [9], which aims to ensure the sustainable management of forestry. Previous research shows that these concessions can have a positive impact on reducing forest loss, especially those certified under responsible forestry schemes [29,57,58,70]."

8. Note that OECMs are a label applied to area-based governance systems for conservation accounting purposes, not a form of governance themselves. The manuscript generally avoids conflating the governance system (e.g., "forest concessions") with the OECM label – please continue to make this distinction and perhaps even make the point more clearly and forcefully.

Based on the reviewer suggestion we have now added an explicit definition of how a governance mechanism can be listed as an OECM in lines 78 to 83;

"In response, much attention is now being placed on other effective area-based conservation measures (OECMs) that can deliver positive conservation outcomes to complement those provided by PAs [25,35]. For a governance mechanism to be listed as an OECM, it cannot be already classified as a PA, and must be a geographically defined area, which is governed and managed in ways that achieve positive and sustained long-term outcomes for the in-situ conservation of biodiversity [36–38]."

We have also made sure that when the term OECMs is used, it is clear that OECMs are governance mechanisms that acquire the designation as OECMs based on a series of requirements, and that in practice they maintain their original management and purpose.

9. Lines 72-74 imply that protected areas = "strict protection". In fact, protected areas include a wide range of governance and management systems, ranging from "fortress" to sustainable use.

We agree with the reviewer comment. We have now avoided the term "strict" and modified the paragraph to improve the clarity. Now it reads;

"Moreover, in some circumstances, the designation of a PA might not be appropriate or feasible, so other governance mechanisms may be more effective in delivering positive conservation outcomes [26]."

10. The manuscript seems to focus on above ground carbon. Please clarify and acknowledge below ground carbon…

We agree with the reviewer that it is important to acknowledge that below ground carbon is not included in our calculations. We have now included a paragraph in lines 233 to 236 mentioning this;

"In this study, "carbon emissions" refers to emissions "committed" at the time of disturbance or clearing, noting that there may be a time lag until they are "realized". Below ground carbon is not included in these estimations but has been estimated to account for an average 22% in forest ecosystems [43]."

11. What is meant by "artificial control groups"? (lines 212) This makes me think of synthetic controls, yet lines 229-240 suggest a more traditional propensity score approach to matching. Perhaps clarify, especially for readers with only a basic understanding of statistical matching methods.

For this analysis a propensity score matching approach was used. Based on the reviewer recommendation we have now modified this "statistical matching section" to more explicitly explain the process that was used. Now the paragraph in lines 266 to 268 reads;

"To assess the impacts of the governance mechanisms (treatments) on forest loss we used a control intervention study design, with matching analysis, identifying pixels with similar characteristics to the ones from the governance mechanisms (controls) based on a set of defined covariates [71,72]."

12. In the results, there are several points at which differences are reported… but then the manuscript notes that the differences are "not significant" (e.g., lines 280-281). If we're using statistical methods, do we talk about differences as meaningful if they aren't statistically significant? Please modify or justify.

It is common practice to report in the result section the statistics for all the analysis done. We report the results of the matching analysis and the significance of all the governance mechanisms assessed. This information is also explicit in Figure 3d. We do not further discuss the findings of the MCs in the discussion section due to them being non-significant. We have now modified the results section to make it clear that the differences for PAs, ILs, and NTCs are statistically significant while for MCs and LCS the differences are not. Specifically in the lines 350 to 355 we state;

"Our results show that PAs, ILs, NTCs and LCs decrease forest loss compared to the matched control areas. For PAs (p value <0.001), ILs (p value <0.001) and NTCs (p value <0.01), these differences were statistically significant, while for LCs (p value = 0.172) they were not (Fig 3 d). PAs were the most effective in preventing forest loss, avoiding 88% of expected loss, followed by NTCs (66%), ILs (42%), and LCs (24%). On the other hand, MCs showed a 26% increase in expected forest loss, compared to matched control areas. However, this increase was not statistically significant (Fig. 3 d)."

In the new analysis for this updated draft, the impact of LCs on deforestation is also non-significant. Previous analysis in other tropical landscapes have shown that in some instances LCs can have a positive effect preventing forest loss, so we discuss the LCs results in relation to other scientific information in the literature in the discussion section in lines 423 to 435. We highlight the fact that our results are non-significant there as well;

"Further, we found that LCs avoided 24% of the deforestation and 18% of the expected carbon emissions between 2005 and 2021, compared to similar unmanaged areas. While these results were not statistically significant and LCs do not meet all the criteria to be recognized as OECMs, these concessions do constitute a considerable proportion of the Peruvian Amazon (8.1%), and so they can still make a significant contribution to other conservation goals like target 10 of the GBF [9], which aims to ensure the sustainable management of forestry. Previous research shows that these concessions can have a positive impact on reducing forest loss, especially those certified under responsible forestry schemes [29,57,58,70]. The further expansion of this type of forest use and governance could prevent widespread illegal logging activities due to an incentive to defend forest assets, and timber profits, by excluding other illegal and legal actors interested in extractive activities (e.g. logging, mining, or land-grabbing) [57,70]. Despite the potential benefits of well-managed LCs in comparison to other extractive activities, it is still important to note that any logging has a negative impact on tropical forest ecosystems, as the extraction of trees degrades the forest [55,56]."

13. The results talk about total and % forest loss across governance types without normalizing these values to account for discrepancies in the total area governed. This leads the reader astray, especially since the total area (and proportion) of land area held under these different regimes is not emphasized in the manuscript. Better to have the normalized data in the main text and a supplemental figure or table with the absolute values.

We agree with the reviewer that area and percentage statistics for total area governed was lacking in the manuscript text. While this is included in Figures 3 and 4, this was not

explained in the figures legend. We have now included this information in the results section in lines 311 to 314;

"The extent of forest in the Peruvian Amazon in 2005 was 701,584 km² (Fig. 3). There was a total of 24 PAs, 2590 ILs, 973 NTCs, 482 LCs and 1085 MCs assessed with a forest extent of 122,096 km², 140,571 km 9,414 km², 57,047 km² and 1,811 km² respectively (Fig. 4). This corresponded to 17,4%, 20%, 1,3%, 8,1% and 0,3% of the Peruvian Amazon forest, respectively."

We purposely included the percentage of forest loss by year (Figure 3 b and Figure 4 b) as this is a standardized way of showing the results (0 to 100% of the total amount of forest present in each governance regime at 2005). We also included the absolute forest area loss (Figure 3 a and 4 a) to show the reader which governance regimes had the lowest and highest absolute loss of forest in km². All the Y axis are standardized so the reader can visually compare proportional and absolute differences between governance regimes. The MCs axis is not standardized as the proportional loss of forest is two orders of magnitude bigger than the other governance regimes while the absolute loss of forest is an order of magnitude smaller. The total amount of forest in km² is also provided in Figure 3 (for the Peruvian Amazon) and in Figure 4 (For each governance mechanism). We have now modified the legend of figures 3 and 4 to include a more detailed explanation of each figure. Figure 3 legend now reads;

"Figure 3. Yearly absolute area (A), percentage (B) and cumulative percentage (C) of forest lost and carbon emissions for the Peruvian Amazon between 2005 and 2021. Vertical bars show forest loss and red lines carbon emissions. In parenthesis is shown the total area of forest in 2005 in km² for the Peruvian Amazon. The left vertical axis shows absolute (km²) and proportional (%) forest loss. The right vertical axis shows absolute (tones) and proportional (%) carbon emissions. (D) Estimated proportional forest loss and carbon emissions inside Protected Areas (PAs), Indigenous land (ILs), Non-Timber Forest Products (NTCs), Logging and Mining Concessions (MCs) in the Peruvian Amazon from 2005 to 2021 against analogous matched areas. black lines, 95% CI; Percentage values on top of each governance mechanism reflect the decrease (green) or increase (red) in deforestation proportional to the matched control pixels. Statistically difference between control and treatment were calculated with a logistic regression, *$p < 0.05$, **$p < 0.01$, ***$p < 0.001$. (E) Median and dispersion of the carbon density (tonnes of CO_2 per 30 m pixels) in the area base interventions, the wider landscape without any area base intervention and the matched control pixels. Statistically difference between the groups were calculated with a Kruskal–Wallis test *$p < 0.05$, **$p < 0.01$, ***$p < 0.001$. In parentheses is the sample size corresponding to the number of pixels in the treatment as well as the control used for the matching analysis for each governance mechanism assessed."

Figure 4 legend now reads;

"Figure 4. Yearly absolute area (A), percentage (B) and cumulative percentage (C) of forest lost and carbon emissions inside Protected Areas, Indigenous Land, Non-Timber Forest Products, Logging and Mining concessions in the study area between 2005 and 2021. Each governance mechanisms is depicted in a different colour. In parenthesis is shown the total area of forest in 2005 in km² for each governance mechanism. The left vertical axis shows

absolute (km²) and proportional (%) forest loss. The right vertical axis shows absolute (tons) and proportional (%) carbon emissions. Vertical bars show forest loss and red lines carbon emissions. All the vertical axes are standardized with the exception of mining concessions."

14. The writing is generally ok. Occasionally, however, awkward phrasing is distracting and can hinder understanding. It would strengthen the manuscript to have a careful copy edit by a native English speaker who is a very strong writer.

We have now a native English speaker among the authors who has proofread the new manuscript to make sure the grammar and phrasing is smooth.

Reviewer #3 (Remarks to the Author):

I co-reviewed this manuscript with one of the reviewers who provided the listed reports. This is part of the Nature Communications initiative to facilitate training in peer review and to provide appropriate recognition for Early Career Researchers who co-review manuscripts.

We thank the reviewer for the feedback and suggestions provided. We have generated new analysis, modified the figures and edited the document extensively to account for the reviewers' comments.

References:

1. UNEP-WCMC & IUCN. World Database on Other Effective Area-based Conservation Measures (WD-OCEM). *Protected Planet* https://www.protectedplanet.net/en/thematic-areas/oecms?tab=OECMs (2025).
2. Jonas, H. D. *et al.* Global status and emerging contribution of other effective area-based conservation measures (OECMs) towards the '30x30' biodiversity Target 3. *Front. Conserv. Sci.* **5**, 1447434 (2024).
3. Cook, C. N. Diverse approaches to protecting biodiversity: The different conservation measures discussed as possible other effective area-based conservation measures. *Conserv. Lett.* e13027 (2024) doi:10.1111/conl.13027.
4. *The Global Assessment Report of the Intergovernmental Science-Policy Platform on Biodiversity and Ecosystem Services*. (Intergovernmental Science-Policy Platform on Biodiversity and Ecosystem Services (IPBES), Bonn, 2019).
5. Alejo, C. *et al.* Are indigenous territories effective natural climate solutions? A neotropical analysis using matching methods and geographic discontinuity designs. *PLoS ONE* **16**, (2021).
6. Sze, J. S., Carrasco, L. R., Childs, D. & Edwards, D. P. Reduced deforestation and degradation in Indigenous Lands pan-tropically. *Nat. Sustain.* **5**, 123–130 (2022).
7. Vélez, M. A., Robalino, J., Cardenas, J. C., Paz, A. & Pacay, E. Is collective titling enough to protect forests? Evidence from Afro-descendant communities in the Colombian Pacific region. *World Dev.* **128**, 2000–2005 (2020).
8. Nepstad, D. *et al.* Inhibition of Amazon Deforestation and Fire by Parks and Indigenous Lands. *Conserv. Biol.* **20**, 65–73 (2006).
9. CBD. *Kunming-Montreal Global Biodiversity Framework*. https://www.cbd.int/doc/decisions/cop-15/cop-15-dec-04-en.pdf (2022).
10. UNFCCC. *Adoption of the Paris Agreement. Proposal by the President Draft Decision -/CP.21*. (2015).

11. Sze, J. S., Carrasco, L. R., Childs, D. & Edwards, D. P. Reduced deforestation and degradation in Indigenous Lands pan-tropically. *Nat. Sustain.* **5**, 123–130 (2022).
12. UNEP-WCMC & IUCN. Protected Areas (WDPA). *Protected Planet* https://www.protectedplanet.net/en/thematic-areas/wdpa?tab=About (2025).
13. Jones, K. R. *et al.* One-third of global protected land is under intense human pressure. *Science* **360**, 788–791 (2018).
14. Grantham, H. S. *et al.* Anthropogenic modification of forests means only 40% of remaining forests have high ecosystem integrity. *Nat. Commun.* 1–10 (2020) doi:10.1038/s41467-020-19493-3.
15. Heino, M. *et al.* Forest loss in protected areas and intact forest landscapes: A global analysis. *PLoS ONE* **10**, 1–21 (2015).
16. Pillay, R. *et al.* Tropical forests are home to over half of the world's vertebrate species. *Front. Ecol. Environ.* **20**, 10–15 (2022).
17. Lewis, S. L., Edwards, D. P. & Galbraith, D. Increasing human dominance of tropical forests. *Science* **349**, 827–832 (2015).
18. Mori, A. S., Lertzman, K. P. & Gustafsson, L. Biodiversity and ecosystem services in forest ecosystems: a research agenda for applied forest ecology. *J. Appl. Ecol.* **54**, 12–27 (2017).
19. Strand, J. *et al.* Spatially explicit valuation of the Brazilian Amazon Forest's Ecosystem Services. *Nat. Sustain.* **1**, 657–664 (2018).
20. Maxwell, S. L. *et al.* Degradation and forgone removals increase the carbon impact of intact forest loss by 626%. *Sci. Adv.* **5**, (2019).
21. Albert, J. S. *et al.* Human impacts outpace natural processes in the Amazon. *Science* (2023) doi:10.1126/science.abo5003.
22. Lapola, D. M. *et al.* The drivers and impacts of Amazon forest degradation. *Science* **349**, (2023).
23. Achard, F. *et al.* Pan-tropical monitoring of deforestation. *Environ. Res. Lett.* **2**, 045022 (2007).
24. Schleicher, J. *et al.* Protecting half of the planet could directly affect over one billion people. *Nat. Sustain.* **2**, 1094–1096 (2019).
25. Oldekop, J. A., Holmes, G., Harris, W. E. & Evans, K. L. A global assessment of the social and conservation outcomes of protected areas. *Conserv. Biol.* **30**, 133–141 (2016).
26. Negret, P. J., Venegas, R., Sonter, L. J., Possingham, H. P. & Maron, M. Conservation planning for retention, not just protection. *Glob. Change Biol.* **30**, e17211 (2024).
27. Donald, P. F. *et al.* The prevalence, characteristics and effectiveness of Aichi Target 11′s "other effective area-based conservation measures" (OECMs) in Key Biodiversity Areas. *Conserv. Lett.* **12**, (2019).
28. Dudley, N. *et al.* The essential role of other effective area-based conservation measures in achieving big bold conservation targets. *Glob. Ecol. Conserv.* **15**, e00424 (2018).
29. Schleicher, J., Peres, C. A., Amano, T., Llactayo, W. & Leader-Williams, N. Conservation performance of different conservation governance regimes in the Peruvian Amazon. *Sci. Rep.* **7**, 1–10 (2017).
30. Cook, C. N. Progress developing the concept of other effective area-based conservation measures. *Conserv. Biol.* **38**, e14106 (2024).
31. Móstiga, M., Armenteras, D., Vayreda, J. & Retana, J. Two decades of accelerated deforestation in Peruvian forests: a national and regional analysis (2000–2020). *Reg. Environ. Change* **24**, 42 (2024).
32. Joppa, L. & Alexander, P. Reassessing the forest impacts of protection. *Ann. N. Y. Acad. Sci.* **1185**, 135–149 (2010).
33. Joppa, L. N. & Pfaff, A. Global protected area impacts. *Proc. R. Soc. B Biol. Sci.* **278**, 1633–1638 (2011).
34. Joppa, L. N. & Pfaff, A. High and far: Biases in the location of protected areas. *PLoS ONE* **4**, 1–6 (2009).
35. Maxwell, S. L. *et al.* Area-based conservation in the twenty-first century. *Nature* **586**, 217–227 (2020).

36. IUCN. *Recognising and Reporting Other Effective Area-Based Conservation Measures*. (IUCN, International Union for Conservation of Nature, 2019). doi:10.2305/IUCN.CH.2019.PATRS.3.en.
37. Jonas, H. D., MacKinnon, K., Marnewick, D. & Wood, P. *Site-Level Tool for Identifying Other Effective Area-Based Conservation Measures (OECMs)*. (IUCN, International Union for Conservation of Nature, 2023). doi:10.2305/WZJH1425.
38. CBD. *Protected Areas and Other Effective Area-Based Conservation Measures*. https://www.cbd.int/doc/decisions/cop-14/cop-14-dec-08-en.pdf (2018).
39. Potapov, P. V. *et al.* National satellite-based humid tropical forest change assessment in Peru in support of REDD+ implementation. *Environ. Res. Lett.* **9**, 2000–2010 (2014).
40. MINAM. *Protocolo Metodológico Para La Detección de La Pérdida de Bosque*. (2021).
41. Asner, G. P. *et al.* Targeted carbon conservation at national scales with high-resolution monitoring. *Proc. Natl. Acad. Sci. U. S. A.* **111**, E5016–E5022 (2014).
42. Asner, G. P. *et al.* A universal airborne LiDAR approach for tropical forest carbon mapping. *Oecologia* **168**, 1147–1160 (2012).
43. Ma, H. *et al.* The global distribution and environmental drivers of aboveground versus belowground plant biomass. *Nat. Ecol. Evol.* **5**, 1110–1122 (2021).
44. Jones, K. R. *et al.* One-third of global protected land is under intense human pressure. *Science* **360**, 788–791 (2018).
45. Negret, P. J. *et al.* Effects of spatial autocorrelation and sampling design on estimates of protected area effectiveness. *Conserv. Biol.* **34**, 1452–1462 (2020).
46. Graham, V. *et al.* Southeast Asian protected areas are effective in conserving forest cover and forest carbon stocks compared to unprotected areas. *Sci. Rep.* **11**, 1–12 (2021).
47. Wauchope, H. S. *et al.* What is a unit of nature? Measurement challenges in the emerging biodiversity credit market. *Proc. R. Soc. B Biol. Sci.* **291**, 20242353 (2024).
48. Duncanson, L. *et al.* The effectiveness of global protected areas for climate change mitigation. *Nat. Commun.* **14**, 2908 (2023).
49. Graham, V. *et al.* Southeast Asian protected areas are effective in conserving forest cover and forest carbon stocks compared to unprotected areas. *Sci. Rep.* **11**, 1–12 (2021).
50. West, T. A. P. *et al. Action Needed to Make Carbon Offsets from Tropical Forest Conservation Work for Climate Change Mitigation Thales*. (2023).
51. Duarte, D. P. & Walker, B. Reducing natural vegetation loss in Amazonia critically depends on the formal recognition of indigenous territories. **279**, (2023).
52. Camino, M. *et al.* Indigenous Lands with secure land-tenure can reduce forest-loss in deforestation hotspots. *Glob. Environ. Change* **81**, 102678 (2023).
53. Dos Santos, A. M., Da Silva, C. F. A., De Melo, S. N., De Almeida Junior, P. M. & Bueno, L. F. Influence of deforestation inside and outside indigenous lands in the Brazilian Amazon Biome. *Reg. Environ. Change* **22**, 77 (2022).
54. Siqueira-Gay, J., Soares-Filho, B., Sanchez, L. E., Oviedo, A. & Sonter, L. J. Proposed Legislation to Mine Brazil's Indigenous Lands Will Threaten Amazon Forests and Their Valuable Ecosystem Services. *One Earth* **3**, 356–362 (2020).
55. Clark, C. J., Poulsen, J. R., Malonga, R. & Elkan, P. W. Logging concessions can extend the conservation estate for central African tropical forests. *Conserv. Biol.* **23**, 1281–1293 (2009).
56. Pearson, T. R. H., Brown, S. & Casarim, F. M. Carbon emissions from tropical forest degradation caused by logging. *Environ. Res. Lett.* **9**, 034017 (2014).
57. Panlasigui, S., Rico-Straffon, J., Pfaff, A., Swenson, J. & Loucks, C. Impacts of certification, uncertified concessions, and protected areas on forest loss in Cameroon, 2000 to 2013. *Biol. Conserv.* **227**, 160–166 (2018).
58. Gaveau, D. L. A. *et al.* Reconciling Forest Conservation and Logging in Indonesian Borneo. *PLoS ONE* **8**, e69887 (2013).
59. World Wildlife Fund. PADDDtracker.org. *PADDDtracker* https://www.padddtracker.org/ (2025).
60. Congress of the Republic of Peru. *Ley de Comunidades Nativas y de Desarrollo Agrario de la Selva y Ceja de Selva - Nº 22175*. https://www.pj.gob.pe/wps/wcm/connect/fbb50c804c7e5783adf4efe93f7fa794/Decreto+Ley+221

75+Ley+de+Comunidades+Nativas+y+desarrollo+agrario+de+las+regiones+de+la+Selva+y+Ceja+de+Selva.pdf?MOD=AJPERES&CACHEID=fbb50c804c7e5783adf4efe93f7fa794 (1991).
61. Congress of the Republic of Peru. *Constitucion Politica del Peru*. https://www.oas.org/juridico/spanish/per_res17.pdf (1993).
62. Golden Kroner, R. E. *et al.* The uncertain future of protected lands and waters. *Science* **364**, 881–886 (2019).
63. Forrest, J. L. *et al.* Tropical Deforestation and Carbon Emissions from Protected Area Downgrading, Downsizing, and Degazettement (PADDD). *Conserv. Lett.* **8**, 153–161 (2015).
64. Pack, S. M. *et al.* Protected area downgrading, downsizing, and degazettement (PADDD) in the Amazon. *Biol. Conserv.* **197**, 32–39 (2016).
65. Tesfaw, A. T. *et al.* Land-use and land-cover change shape the sustainability and impacts of protected areas. *Proc. Natl. Acad. Sci.* **115**, 2084–2089 (2018).
66. Peruvian State. Áreas de Conservación Privada (ACP). *National Open Data Platform* https://www.datosabiertos.gob.pe/dataset/%C3%A1reas-de-conservaci%C3%B3n-privada-acp (2025).
67. Peruvian State. Áreas de Conservacion Regional (ACR). *National Open Data Platform* https://www.datosabiertos.gob.pe/dataset/%C3%A1reas-de-conservacion-regional-acr (2025).
68. Anderson, C. M., Asner, G. P., Llactayo, W. & Lambin, E. F. Overlapping land allocations reduce deforestation in Peru. *Land Use Policy* **79**, 174–178 (2018).
69. *Catastro formación, investigación y empresa: selección de ponencias del I Congreso Internacional sobre Catastro Unificado y Multipropósito*. (Universidad de Jaén, Jaén, 2010).
70. Rico-Straffon, J. *et al.* Forest concessions and eco-certifications in the Peruvian Amazon: Deforestation impacts of logging rights and logging restrictions. *J. Environ. Econ. Manag.* **118**, 102780 (2023).
71. Negret, P. J. *et al.* Spatial autocorrelation and sampling design affect estimates of protected area effectiveness. *Conserv. Biol.* **34**, 1452–1462 (2020).
72. Schleicher, J. *et al.* Statistical matching for conservation science. *Conserv. Biol.* **00**, 1–12 (2019).

u^b UNIVERSITÄT BERN

WYSS ACADEMY FOR NATURE

__Response to reviewers' comments__

We have responded in **bold** to all the comments.

Reviewer #1 (Remarks to the Author):

I value the intense work undertaken by the authors in response to the reviews. I am content now that this could be a good ms for publication.

We thank the reviewer for the positive feedback and his suggestion for acceptance.

Reviewer #2 (Remarks to the Author):

Major comments

1. Methods: Further clarification is required regarding treatment and control site selection. As I understand it: (i) the study area is the biogeographic boundary of the Peruvian Amazon; (ii) the treatment sites were all established prior to 2006 and fall into one of 5 governance systems (PAs, ILs, NTCs, LCs, or MCs); and (iii) potential control sites include the entire non-treatment area of the Peruvian Amazon, except for a buffer of 1 km (NTCs, LCs, MCs and ILs) and 5 km (PAs) around treatment areas.

This is correct

This implies that the following areas are potential control sites:

a. Sites with multiple "stacked" treatments (e.g., indigenous lands with a logging concession)

We did remove the treatment sites with overlapping governance mechanisms. This is explained in the methods section in lines 282 to 285;

"We used forest loss between 2005 and 2021 as the outcome variable for our matching analysis. We excluded from the analysis those sites with governance mechanisms established after 2005, areas of overlap between sites with different governance mechanisms and sites that previously had one of the governance mechanisms in place but did not during the study period."

b. Sites with other area-based governance systems in place with conservation intent or potential (e.g., payments for ecosystem services)

There are no spatially explicit datasets for the Peruvian Amazon that comprehensively capture which properties have conservation intent or potential (Lagneaux et al., 2024), so we did not include them in our analysis. We mention now this in methods in lines 285 to 287;

"There are no spatially explicit datasets for the Peruvian Amazon that comprehensively capture region specific sites with governance mechanisms that have conservation potential (Lagneaux et al., 2024)***, so these are not included in our analysis."***

We also mention this in the discussion in lines 490 to 498;

"The results of our analysis comparing the effectiveness of potential OECMs preventing forest loss in the Peruvian Amazon provides an overview of the impact of these governance mechanisms avoiding deforestation and its associated carbon emissions, but there are some caveats that must be accounted for. First, for this analysis we assessed the most widely distributed governance mechanisms in the Peruvian Amazon, but there are other governance mechanisms with potential to be listed as OECMs, including private (Peruvian State, 2025a) ***and regional*** (Peruvian State, 2025b) ***conservation areas, that we could not assess. While there are no systematic datasets on the distribution and characteristics of sites with these governance mechanisms*** (Lagneaux et al., 2024)***, it would be valuable to evaluate their impact on biodiversity conservation in future studies and assess their potential to be listed as OECMs."***

c. Sites that previously had one of these 5 "treatment" governance systems in place, but did not during the study period (e.g., PADDDed protected areas, canceled forest concessions).

Pixels in sites that had any of the five "treatment" governance mechanisms in place before the study period but did not during the study period have now been removed from the analysis.

d. Sites that established one of these 5 "treatment" governance systems during the period 2006-2025.

Pixels in sites where any of the five "treatment" governance systems was stablished after 2006 have now been removed from the analysis.

Is this list of potential control sites accurate? None of these potential control sites (a-d) can be considered true controls that are independent of the treatment, since they themselves are governance treatments that may affect the endpoints examined in this study (forest cover and carbon emissions). The estimated impacts resulting from such an analysis is confounded by such an analysis of "false controls".

Please omit any false controls from the analysis and, in the methods, describe the residual forms of forest governance that remain within the pool of prospective control sites/pixels. Also, please clarify whether the pool of potential control sites is the same for all treatments or if there are different pools of potential control sites for each of the 5 treatments.

We have now removed the potential false controls in the study area to the best of our capabilities. We also describe the potential residual forms of governance mechanisms that remain within the pool of prospective control pixels in lines 282 and 287;

"We used forest loss between 2005 and 2021 as the outcome variable for our matching analysis. We excluded from the analysis those sites with governance mechanisms established after 2005. Areas of overlap between sites with different governance mechanisms and sites that previously had one of the governance mechanisms in place but did not during the study

period. There are no spatially explicit datasets for the Peruvian Amazon that comprehensively capture region specific sites with governance mechanisms that have conservation potential (Lagneaux et al., 2024)***, so these are not included in our analysis."***

The pool of potential control pixels is the same for all the five governance mechanisms. This is explained in lines 325 to 329;

"We matched each treatment observation to a unique control observation to ensure each governance mechanism pixel was paired with only one control pixel that had not been matched previously. Matched pairs of pixels had to be within 0.2 SD of the propensity scores (Austin, 2011; Lunt, 2014)***. Pixels could be reselected as control samples for each independent governance mechanism analysis."***

2. Methods. Line 265. As noted in my previous review, survivorship bias (i.e., only examining impact of interventions that survive/persist until the conclusion of the study period) can bias research findings (since "survivors" are dissimilar from non-survivors, given that the survivors have survived to the end of the study period!). Accordingly, in this case, mining concessions that stopped, were revoked, etc., during the study period should remain within the "treatment" group and results should be reported accordingly. (Since the team has already completed analysis with some discontinued mining concessions examined for impact, these "survivors only" results should be included in the supplemental materials.)

We have now included the sites that stopped operating between 2005 and 2021 in the treatment group for the results of the main analysis. We have also generated a supplementary analysis excluding the sites with governance mechanisms that stopped operating between 2005 and 2021. Both analyses show similar results (Fig 3 d - e & S Fig. 2). We mention this in methods in lines 287 to 290;

"Sites that stopped operating between 2005 and 2021 were included in the analysis. A supplementary analysis excluding the sites that stopped operating between 2005 and 2021 provided similar results (Supplementary Fig. 2)."

3. Methods: A control-intervention study design with matching is stronger than such a design without matching, but obviously not as strong as before-after-control-intervention. One method that helps ensure the validity of control-intervention designs is to test for and show parallel trends in outcome variables prior to the study period, yet I do not see mention of this in the methods. Am I missing it? If not, please conduct and include such a parallel trends analysis in the study design or justify why such an analysis is not necessary in this study.

We have now included the deforestation trends for each pixel at local (1 km radius) and landscape (10 km radius) level from 2000 to 2005 as covariates in the matching analysis. In this way our matched pixels now have comparable deforestation trends prior to the study period. This is described in methods in lines 275 to 279;

"We also included the deforestation trends around each pixel between 2000 and 2005 at local (one-kilometre radius) and landscape (ten-kilometre radius) scale as covariates in the

matching analysis to ensure our matched pixels had comparable deforestation trends prior to the study period (Table 1).”

4. Methods: lines 275+. Although commonly used when evaluating forest outcomes of conservation initiatives, the use of pixel-level comparisons to estimate treatment effects raises questions about pseudo-replication. That is, because protected areas, indigenous lands, and other area-based forest governance systems are managed at the site/location/entire jurisdiction level (e.g., an entire protected area, rather than at the level of a pixel within a protected area), the pixel-level observations within a protected area are not independent of each other… and, thus, measurements of 500 pixels across 5 protected areas is an assessment of 5 independent treatments (not 500).
With these concerns in mind, the manuscript needs to specify the number of sites/locations/jurisdictions (e.g., protected areas) included for each treatment category, as well as the number of total observations made (pixels) for each treatment category and the distribution of these observations for each category across the number of sites. The manuscript also needs to address the issue of pseudo-replication, either by modifying its methods and/or by justifying its approach to sampling and analysis.

We have now accounted for spatial autocorrelation and pseudo-replication in our analysis by explicitly testing for autocorrelation between pixels and removing the pixels with high autocorrelation. We used a global Moran’s I to estimate the level of spatial autocorrelation between pixels in our case study area and a local Moran’s I to locally identify the pixels with the highest spatial autocorrelation in the landscape. We then iteratively removed the pixels with higher autocorrelation until the global Moran’s I was non different from 0 at a distance below 1000 meters. This is a frequently used method to account for spatial autocorrelation (Negret et al., 2020; Zou et al., 2025). This is explained in lines 314 to 320;

“We explicitly accounted for spatial autocorrelation and pseudo-replication by estimating the residual spatial autocorrelation of these models (Moran’s I) and then removing the pixels highly autocorrelated from the analysis. We did this through the use of a global Moran’s I to estimate the level of spatial autocorrelation between pixels in our case study area (the Peruvian Amazon) and a local Moran’s I to identify locally the pixels with the highest spatial autocorrelation (Anselin, 1995)***. We then iteratively removed the pixels with the highest autocorrelation until the global Moran’s I was non different from 0 at a distance below 1000 meters*** (Negret et al., 2020; Zou et al., 2025) ***(Supplementary Information 1).”***

We have also now done additional post-matching analysis at the site level to contrast with the analysis done at the pixel level. This is explained now in lines 336 to 340;

“We estimated summary statistics by calculating average deforestation based on pixels (which up-weigh larger sites). We also did the summary statistics at site level by first summarizing deforestation rates in all pixels within each site before estimating the overall effects based on average values across sites (which up-weighs small sites) (Geldmann et al., 2019)***.”***

For all governance mechanisms with the exception of IL, effectiveness patterns for the different governance mechanisms were similar. For IL the differences are due to the high proportion of small sites being ineffective at reducing deforestation while fewer large size sites showing effectiveness preventing deforestation (Fig. 5). This is explained in the results section in lines 394 to 400;

"When the results were aggregated at the site level, we found similar trends to the ones found at the pixel level, except for ILs which showed higher average forest loss per site in the treatment areas than in controls (Supplementary Table 2 & Fig. 5). For ILs there is a higher number of smaller sites than larger ones (Supplementary Fig. 6) and those sites represent a higher proportion of the total pixels used in the analysis (Supplementary Fig. 7). These smaller sites tend to be ineffective preventing deforestation, so the mean avoided deforestation by site is strongly influenced by those small ineffective sites, despite larger sites tending to be effective in reducing deforestation (Fig. 5)."

Explicitly in relation to the request to specify the number of sites/locations/jurisdictions (e.g., protected areas) included for each treatment category, we do this now in Figure 5 in the top frame in parenthesis after the name of each treatment category, this is described in the figure legend. For the number of total observations made (pixels) for each treatment category, we specify this now in Figure 3 in the bottom frame in parenthesis after the name of each treatment category, this is described in the figure legend. We have also now generated additional figures describing the number of sites by ranges of site area as well as the number of pixels by sites by ranges of site area for each governance mechanisms (S Fig. 6 & 7).

It is important to highlight that while the use of the pixel or site to present the results has to be defined in a case by case basis, showing the results at the pixel level is predominately used when measuring deforestation and land cover change (Brodie et al., 2025; Engert et al., 2024; Geldmann et al., 2019; Graham et al., 2021; Negret et al., 2020; Zou et al., 2025)**.**

5. Results/methods – statistical matching (lines 343-353). The covariate imbalances for non-timber concessions (NTCs) are quite high, making use of these statistical models to estimate treatment effects highly concerning. Given the sensitivity of estimated treatment effects to match specifications, and the match imbalances reported here, further model refinement is necessary and/or further justification is required for reporting results that do not adhere to basic guidelines for use of propensity score matching methods. It may make sense to omit the NTC treatment group, since including a bad match could bias the reader's view of the entire study.

Likewise, given the substantial imbalance in "distance to settlement" for protected areas and their controls, further model refinement is necessary and/or further justification is required for inclusion of results from the protected area (PA) treatment.

We re-ran the analysis and incorporated additional steps into the matching procedure to ensure that, for all governance mechanisms assessed, covariate imbalances were reduced to below 25%. This process is now described in the Methods section in lines 325–336;

"We matched each treatment observation to a unique control observation to ensure each governance mechanism pixel was paired with only one control pixel that had not been matched previously. Matched pairs of pixels had to be within 0.2 SD of the propensity scores (Austin, 2011; Lunt, 2014)***. Pixels could be reselected as control samples for each independent governance mechanism analysis.***
We discarded any treatment pixel where the distance in propensity scores between treatment and control was >0.2 to remove potential outliers (Geldmann et al., 2019; Lunt, 2014)***. We then calculated the indices of covariate imbalance for every matching analysis for each governance mechanism to assess the robustness of the matching analysis reducing bias in the control units. Absolute scores >25% are considered an indication of a possible imbalance for that specific variable*** (Negret et al., 2020; Olmos and Govindasamy, 2015; Zou et al., 2025)***. Overall, there was notable improvements reducing bias in the control units through the matching analysis (Supplementary Fig. 3)."***

6. Results & discussion – All mention of PAs and NTCs in the results and discussion need to be reconsidered based on refinements to study design and statistics. Discussion of logging concessions (LCs) and their potential as a conservation strategy also needs to be revisited, given the non-significant impact on forests and carbon emissions reported.

We re-ran the analysis and incorporated additional steps into the matching procedure to ensure that, for all governance mechanisms assessed, covariate imbalances were reduced to below 25%. For our new analysis all the differences between treatment and controls were significant (Fig. 3). Our results now indicate that, overall, LCs experience higher deforestation than controls; however, considerable variability exists across sites (Figure 5). We describe this new results in lines 405 to 405;

"For LCs, smaller sites tended to be ineffective at preventing deforestation, and some larger sites were effective."

And in the discussion section in lines 433 to 436;

"While our results show that different governance mechanisms in the Peruvian Amazon can reduce forest loss, we also found that variation in effectiveness by site is high and that effectiveness can vary with site size. Smaller ILs and LCs tend to be less effective preventing forest loss, while larger NTCs tend to be more effective."

7. Discussion – Please discuss the study's findings in the context of previous research, rather than reiterating/re-summarizing the study's results (e.g., lines 387-393). There is mention of prior research by others, but it would strengthen the manuscript to go farther. What previous findings does this study reaffirm? For both science and policy, what prior findings/conventional wisdom does this study challenge, what new questions does it raise, and new directions does it suggest?

We have removed the sections of the discussion that were redundant with the results section and substantially revised it based on the findings of the new analysis. The revised

discussion addresses both the new and previous results within the context of the most up-to-date literature on the subject.

Other major comments:
8. Methods: The global analysis distinguishes between protected areas in IUCN categories I-II and those in categories III-VI. Given the differences among protected areas in these two subgroupings at the global level, the Peru-specific component of the manuscript should make a similar distinction or justify why such a distinction is not appropriate in the study design and analysis. At the least, descriptive data and discussion of regarding the Peruvian conservation treatments should include IUCN categories for the relevant protected areas.

We now present the results for protected areas in the Peruvian Amazon separated into two groups—IUCN categories I–II and categories III–VI—consistent with the approach used in the global analysis and in other previous papers (Jones et al., 2018). We mention this in the methods in lines 213 to 216;

"As it was the case for the global analysis here we also grouped PAs into the ones with strict biodiversity conservation objectives (IUCN categories I and II) and the ones permitting certain human activities and sustainable resource extraction (IUCN categories III to VI) (Jones et al., 2018)***."***

9. Results: Lines 320-322 – The numbers here seem high (e.g., 2590 Indigenous Lands); do these numbers represent designated territories/legal designations, individual pixels, or something else? Please clarify. See Qin et al (2024): One Earth 7(6) 962-975

The numbers here represent the number of sites included for this governance mechanisms for the analysis. After re-structuring the methods there are now 1250 sited under Indigenous governance included in the analysis. We now provide the information of the number of sites included in the analysis for each governance mechanism in Figure 5.

We have now explicitly clarified the definition of what we mean by a governance mechanism in the introduction in lines 91 to 93;

"Here we define governance mechanisms as the system of laws, norms, rules, policies and practices that determine how landscapes and resources are managed and regulated in a spatially defined area (Zhang et al., 2023)***."***

We have also clarified throughout the manuscript whether the analyses were conducted at the pixel level or at the site level.

10. Results – line 346-347: imbalanced matching for PAs. Does this mean that pixelated observations within PAs were closer to settlements or farther from settlements, relative to the pixels against which they were matched? This obviously matters, given the impact estimate of dramatically reduced deforestation because of protected area establishment.

We re-ran the analysis and incorporated additional steps into the matching procedure to ensure that, for all governance mechanisms assessed, covariate imbalances were reduced to below 25% (S Fig. 3).

11. Discussion. Lines 380-381. "…we can conclude that…" This conclusion seems misguided, based on inference from the analysis. We know that OECMs and PAs are nonrandomly located (e.g., Joppa & Pfaff's "high and far" analysis), so this observation re: pressure is perhaps simply another way to confirm their observation, rather than an estimate of the impact of these conservation initiatives on human behavior/land use. Appropriate inference and interpretation of the global analysis is particularly important in this manuscript, given the use of matching methods to control spatial biases and allow for more rigorous causal inference.

We have now rephrased the paragraph and clarified that our results at the global level do not estimate the effectiveness of PAs or OECMs preventing biodiversity loss. The paragraph now reads;

"At the global scale, our results show that while OECMs and PAs face considerable human pressure levels, they still have far lower human pressure than unprotected land. Previous work has shown that PAs face widespread levels of intense human pressure (Jones et al., 2018)***, but there is also evidence that they can reduce deforestation and habitat loss*** (Sze et al., 2022)***, and contain higher biodiversity than unprotected areas*** (Gray et al., 2016)***. Here, we showed that, on average, OECMs have similar human pressure levels to PAs, particularly those in IUCN categories III-VI. Less degraded ecosystems, such as those within many PAs and OECMS, support an exceptional confluence of globally significant environmental values relative to the ones that have experienced damaging human actions*** (Watson et al., 2018)***, so both OECMs and PAs have strong potential to deliver positive conservation outcomes. However, delivering on this potential will require concerted actions to overcome the many challenges facing area-based conservation efforts, such as underfinancing and under staffing*** (Coad et al., 2019; Lessmann et al., 2024)***, PADDD*** (Forrest et al., 2015; Golden Kroner et al., 2019)***, and resource extraction*** (Durán et al., 2013)***. Moreover, 42% of all reported OECMs were previously classified as PAs*** (Cook et al., 2024)***, so a better understanding of the causes, consequences and conservation impact of such changes is needed. Importantly, our global analysis does not consider the non-random location of PAs and OECMs and how this affects the effectiveness of these conservation mechanisms preventing biodiversity loss, which should be a priority for future research at the global scale."***

Minor comments

12. Title: "…area-based governance mechanisms…"?

We have now modified the tittle accordingly.

13. Keywords: include "protected areas"? omit something else?

We have now included Protected Areas as a keyword.

14. Lines 55-57: the MS notes later that logging concession cannot be classified as OECMs because of their extractive orientation, yet highlight them here with "…potential to be listed as OECMs…" Please clarify/reconcile discrepancy.

We have now modified this sentence to clarify that LCs do not have the potential to be classified as OECMs. The paragraph now reads;

"We also assessed the impact of two different governance mechanisms with potential to be listed as OECMs - Indigenous Lands (ILs) and Non-Timber Forest Products Concessions (NTCs) as well as Logging Concessions (LCs), Mining Concessions (MCs) and PAs on forest loss and its associated carbon emissions in the Peruvian Amazon from 2005 to 2021."

15. Lines 78, 80, etc. Is "protection" used intentionally? "Protection" sometimes implies an exclusionary fortress model, whereas 'conservation' often implies a more diverse and potentially inclusive set of governance systems/strategies.

We have changed protection to conservation in this paragraph.

16. Lines 84-85. Note Fedele, et al. 2021. Global Environmental Change (71): more than 2 billion people in tropics depend on nature to meet their basic needs.

We have now included the citation in this paragraph.

17. Lines 92-93. Note that Cook et al (OneEarth 7(3): 412-430) find that many protected areas around the world that have been reclassified as OECMs, now that there is a new accounting system that will allow governments to get credit for them. This observation should be considered and addressed with respect to the global analysis of human pressures within OECMs and PAs.

We thank the reviewer for pointing this out. We have now included an additional paragraph in the introduction in lines 101 to 107 acknowledging this;

"More than 1.9 million km² of the Earth's surface has already been reported to the World Database on OECMs (UNEP-WCMC and IUCN, 2025)***, representing approximately 1% of total terrestrial area (Fig. 1). Two thirds of this area is governed by federal or national agencies*** (Jonas et al., 2024)***. With OECMs rapidly gaining attention and traction in conservation policy, a clear understanding of the effectiveness of various OECM designations as conservation mechanisms is needed*** (Jonas et al., 2024)***. It is also important to improve our understanding of the causes and consequences of PAs and OECMs downgrading, downsizing, and degazettement, given increasing reports of PAs being reclassified as OECMs*** (Cook et al., 2024)***."***

We have also included a paragraph in the discussion in lines 416 to 420 stating the need for more research on this topic:

"However, delivering on this potential will require concerted actions to overcome the many challenges facing area-based conservation efforts, such as underfinancing and under staffing (Coad et al., 2019; Lessmann et al., 2024)***, PADDD*** (Forrest et al., 2015; Golden Kroner et al., 2019)***, and resource extraction*** (Durán et al., 2013)***. Moreover, 42% of all reported OECMs were previously classified as PAs*** (Cook et al., 2024)***, so a better understanding of the causes, consequences and conservation impact of such changes is needed."***

18. Line 96: "this land" – which land? unclear antecedent. Does the MS refer to the entirety of the earth's surface or to the 1% that is within recognized OECMs?

We have now changed the word land for area to improve clarity.

19. Line 100. This is misleading. Not all indigenous lands are recognized as OECMs. Please clarify.

We have now rephrased the paragraph to improve its clarity. Now the paragraph reads;

"Sites recognized as OECMs are governed through diverse mechanisms, including land management by Indigenous Peoples (Cook, 2024; Jonas et al., 2024)***. Emerging evidence demonstrates that Indigenous Lands (ILs), when managed under suitable conditions, can be highly effective in limiting deforestation*** (Alejo et al., 2021; Brondízio et al., 2019; Sze et al., 2022; Vélez et al., 2020)***."***

20. Lines 100-116: reference to Qin et al (2024): One Earth 7(6) 962-975 seems relevant here and throughout the manuscript.

We have now included this citation here and in other sections of the manuscript.

21. Line 108: "ranging from", rather than "including"?

We have changed the wording accordingly

22. Lines 114-115: "could be listed.." could all of these governance systems be recognized as OECMs, given the criteria governing what can be recognized and what cannot?

Yes, these are examples of governance mechanisms of sites that have already been recognized or have the potential to be recognized as OECMs (Cook, 2024; Qin et al., 2024)**. We have now changed the wording to make this paragraph clearer. Now it reads;**

"Other governance mechanisms that have been associated with positive conservation outcomes and that have the potential to be classified as OECMs include those on sacred sites, military areas, private, urban and surfing reserves and university research areas among others (Cook, 2024; Qin et al., 2024)***."***

23. Method: Lines 231. In what way is reforestation/forest regrowth addressed within the study design and analysis?

The estimation of forest regrowth and reforestation was not an objective of this paper. The forest data used for this analysis was the official forest cover data for the country from the Peruvian Ministry of the Environment and the Ministry of Agriculture and Irrigation. This dataset does not include regrowth or reforestation information.

24. Lines 262+. Phrasing about "governance mechanisms" is misleading/confusing. "Governance mechanism" or "governance system" should refer an entire class or category of initiatives (e.g., "protected areas" or "indigenous lands"), with individual "designations" or "sites" or "locations" or "individual protected areas" representing the manifestation/implementation of these mechanisms on the ground. As currently phrased, "mechanism" appears to refer to individual sites, rather than the general systems/classification/family of initiatives to which these sites belong.

The manuscript has been revised for clarity, with a clearer separation between governance mechanisms and sites. We also now define governance mechanisms in the introduction in lines 91 to 93;

"Here we define governance mechanisms as the system of laws, norms, rules, policies and practices that determine how landscapes and resources are managed and regulated in a spatially defined area (Zhang et al., 2023)***."***

We have also now done the analysis both at the site and governance mechanism level providing a more nuanced understanding of the effectiveness of individual sites and of all the sites under a particular governance mechanism.

25. Line 268. The phrase "once designated they acquire this status in perpetuity" can probably be omitted. Protected areas are also designated "in perpetuity" and are conceived as such, yet we have seen widespread PADDD in Peru and around the world during the 20th and 21 centuries. Assertions of legal designations in perpetuity is not the same as empirical research to verify this point; please ensure empirically that no indigenous lands have been downsized or disestablished during the study period.

As mentioned in a previous response, we have now conducted an analysis including the sites that stopped operating between 2005 and 2021 and a supplementary analysis excluding the sites that stopped operating between 2005 and 2021. Both analyses provided similar results (S Fig. 2). This is explained in line 289 to 291;

"Sites that stopped operating between 2005 and 2021 were included in the analysis. A supplementary analysis excluding the sites that stopped operating between 2005 and 2021 provided similar results (Supplementary Fig. 2)."

26. Lines 356-358. It seems inappropriate to say that the "results show that … LCs decrease forest loss compared to matched controls" but that these differences were not statistically

significant.

Description of the impact of mining concessions is similarly problematic, especially since no p-value is reported in the results.

We have now modified the analysis and the wording of the results. The modelled effects of each governance mechanism on deforestation based on the new analysis are significant. The models estimated effect, and its significance are independent from the calculations of the rate of deforestation in the sites under each governance mechanisms and in the controls. We now explain the modeling approach in the method section in lines 346 to 349;

"To assess the effect of each governance mechanisms reducing deforestation we used general linear mixed models with a binomial response (loss=1, no loss=0) and the status of each pixel as the predictor variable (inside vs. outside a site with a particular governance mechanism)."

We have also modified Figure 3 to make the distinction between the statistical significance of the effect (model estimate) of each governance mechanism on deforestation and the difference in deforestation rates between the treatments and the control pixels.

See this article on reporting numeric differences that are not statistically significant: https://onlinelibrary.wiley.com/doi/pdf/10.1111/jan.14283.

We thank the reviewer for pointing out this article.

27. Writing: Further precision in language is necessary to communicate clearly with readers. Please re-read for vague/unclear antecedents, misleading statements, etc. (e.g., Line 54: "their" is unclear; line 74 "they" is unclear, line 111 "their", etc.) Please review MS in full and specify clearly. Perhaps ask non-authors to read the manuscript and highlight ambiguous language.

We have revised the specific paragraphs highlighted by the reviewer to improve their clarity. In addition, the manuscript has been thoroughly proofread by the co-authors, including native English speakers.

References:

Alejo, C., Meyer, C., Walker, W.S., Gorelik, S.R., Josse, C., Aragon-Osejo, J.L., Rios, S., Augusto, C., Llanos, A., Coomes, O.T., Potvin, C., 2021. Are indigenous territories effective natural climate solutions? A neotropical analysis using matching methods and geographic discontinuity designs. PLoS ONE 16. https://doi.org/10.1371/journal.pone.0245110

Anselin, L., 1995. Local Indicators of Spatial Association—LISA. Geographical Analysis 27, 93–115. https://doi.org/10.1111/j.1538-4632.1995.tb00338.x

Austin, P.C., 2011. Optimal caliper widths for propensity-score matching when estimating differences in means and differences in proportions in observational studies. Pharmaceutical Statistics 10, 150–161. https://doi.org/10.1002/pst.433

Brodie, J.F., Deith, M.C.M., Burns, P., Goetz, S., Cunningham, C., Hill, J.K., Reynolds, G., Mohd-Azlan, J., 2025. The contribution of other effective area-based conservation

measures (OECMs) to protecting global biodiversity. Nat Commun 16, 7886. https://doi.org/10.1038/s41467-025-63205-8
Brondízio, E.S., Settele, J., Díaz, S., Ngo, H.T. (Eds.), 2019. The global assessment report of the intergovernmental science-policy platform on biodiversity and ecosystem services. Intergovernmental Science-Policy Platform on Biodiversity and Ecosystem Services (IPBES), Bonn.
Coad, L., Watson, J.E., Geldmann, J., Burgess, N.D., Leverington, F., Hockings, M., Knights, K., Di Marco, M., 2019. Widespread shortfalls in protected area resourcing undermine efforts to conserve biodiversity. Frontiers in Ecol & Environ 17, 259–264. https://doi.org/10.1002/fee.2042
Cook, C.N., 2024. Diverse approaches to protecting biodiversity: The different conservation measures discussed as possible other effective area-based conservation measures. Conservation Letters. e13027. https://doi.org/10.1111/conl.13027
Cook, C.N., Lemieux, C.J., Golden Kroner, R., 2024. Increasing transparency and accountability in global protected and conserved area reporting. One Earth 7, 421–430. https://doi.org/10.1016/j.oneear.2024.01.022
Durán, A.P., Rauch, J., Gaston, K.J., 2013. Global spatial coincidence between protected areas and metal mining activities. Biological Conservation 160, 272–278. https://doi.org/10.1016/j.biocon.2013.02.003
Engert, J.E., Campbell, M.J., Cinner, J.E., Ishida, Y., Sloan, S., Supriatna, J., Alamgir, M., Cislowski, J., Laurance, W.F., 2024. Ghost roads and the destruction of Asia-Pacific tropical forests. Nature 629, 370–375. https://doi.org/10.1038/s41586-024-07303-5
Forrest, J.L., Mascia, M.B., Pailler, S., Abidin, S.Z., Araujo, M.D., Krithivasan, R., Riveros, J.C., 2015. Tropical Deforestation and Carbon Emissions from Protected Area Downgrading, Downsizing, and Degazettement (PADDD). CONSERVATION LETTERS 8, 153–161. https://doi.org/10.1111/conl.12144
Geldmann, J., Manica, A., Burgess, N.D., Coad, L., Balmford, A., 2019. A global-level assessment of the effectiveness of protected areas at resisting anthropogenic pressures. Proceedings of the National Academy of Sciences of the United States of America 1–7. https://doi.org/10.1073/pnas.1908221116
Golden Kroner, R.E., Qin, S., Cook, C.N., Krithivasan, R., Pack, S.M., Bonilla, O.D., Cort-Kansinally, K.A., Coutinho, B., Feng, M., Martínez Garcia, M.I., He, Y., Kennedy, C.J., Lebreton, C., Ledezma, J.C., Lovejoy, T.E., Luther, D.A., Parmanand, Y., Ruíz-Agudelo, C.A., Yerena, E., Morón Zambrano, V., Mascia, M.B., 2019. The uncertain future of protected lands and waters. Science 364, 881–886. https://doi.org/10.1126/science.aau5525
Graham, V., Geldmann, J., Adams, V.M., Negret, P.J., Sinovas, P., Chang, H.-C., 2021. Southeast Asian protected areas are effective in conserving forest cover and forest carbon stocks compared to unprotected areas. Scientific Reports 11, 1–12. https://doi.org/10.1038/s41598-021-03188-w
Gray, C.L., Hill, S.L.L., Newbold, T., Hudson, L.N., Börger, L., Contu, S., Hoskins, A.J., Ferrier, S., Purvis, A., Scharlemann, J.P.W., 2016. Local biodiversity is higher inside than outside terrestrial protected areas worldwide. Nat Commun 7, 12306. https://doi.org/10.1038/ncomms12306
Jonas, H.D., Bingham, H.C., Bennett, N.J., Woodley, S., Zlatanova, R., Howland, E., Belle, E., Upton, J., Gottlieb, B., Kamath, V., Lessmann, J., Delli, G., Dubois, G., Ahmadia, G., Claudet, J., Cook, C., Deza, J., Grorud-Colvert, K., Gurney, G., Lemieux, C.J., Ruiz, L., 2024. Global status and emerging contribution of other effective area-based conservation measures (OECMs) towards the '30x30' biodiversity Target 3. Front. Conserv. Sci. 5, 1447434. https://doi.org/10.3389/fcosc.2024.1447434
Jones, K.R., Allan, J.R., Maxwell, S.L., Fuller, R.A., Venter, O., Watson, J.E.M., Negret, P.J., 2018. One-third of global protected land is under intense human pressure. Science 360, 788–791. https://doi.org/10.1126/science.aap9565

Lagneaux, E.G., Quaedvlieg, J., Sabogal, D., Jansen, M., 2024. Understanding the diversity of private conservation in the Peruvian Amazon. Conservat Sci and Prac e13228. https://doi.org/10.1111/csp2.13228

Lessmann, J., Geldmann, J., Fajardo, J., Marquet, P.A., 2024. The role of funding in the performance of Latin America's protected areas. Proc. Natl. Acad. Sci. U.S.A. 121, e2307521121. https://doi.org/10.1073/pnas.2307521121

Lunt, M., 2014. Selecting an Appropriate Caliper Can Be Essential for Achieving Good Balance With Propensity Score Matching. American Journal of Epidemiology 179, 226–235. https://doi.org/10.1093/aje/kwt212

Negret, P.J., Marco, M.D., Sonter, L.J., Rhodes, J., Possingham, H.P., Maron, M., 2020. Effects of spatial autocorrelation and sampling design on estimates of protected area effectiveness. Conservation Biology 34, 1452–1462. https://doi.org/10.1111/cobi.13522

Olmos, A., Govindasamy, P., 2015. Propensity Scores: A Practical Introduction Using R. Journal of MultiDisciplinary Evaluation 11, 1556–8180.

Peruvian State, 2025a. Áreas de Conservación Privada (ACP) [WWW Document]. National Open Data Platform. URL https://www.datosabiertos.gob.pe/dataset/%C3%A1reas-de-conservaci%C3%B3n-privada-acp

Peruvian State, 2025b. Áreas de Conservacion Regional (ACR) [WWW Document]. National Open Data Platform. URL https://www.datosabiertos.gob.pe/dataset/%C3%A1reas-de-conservacion-regional-acr

Qin, S., He, Y., Golden Kroner, R.E., Shrestha, S., Henriques Coutinho, B., Karmann, M., Ledezma, J.C., Martinez, C., Morón-Zambrano, V., Ulloa, R., Yerena, E., Bernard, C., Bull, J.W., Mendoza, E., De Pracontal, N., Reytar, K., Veit, P., Olsson, E., Matallana-Tobón, C.L., Wily, L.A., Mascia, M.B., 2024. An inclusive, empirically grounded inventory facilitates recognition of diverse area-based conservation of nature. One Earth S2590332224001349. https://doi.org/10.1016/j.oneear.2024.03.005

Sze, J.S., Carrasco, L.R., Childs, D., Edwards, D.P., 2022. Reduced deforestation and degradation in Indigenous Lands pan-tropically. Nature Sustainability 5, 123–130. https://doi.org/10.1038/s41893-021-00815-2

UNEP-WCMC, IUCN, 2025. World Database on Other Effective Area-based Conservation Measures (WD-OCEM) [WWW Document]. Protected Planet. URL https://www.protectedplanet.net/en/thematic-areas/oecms?tab=OECMs

Vélez, M.A., Robalino, J., Cardenas, J.C., Paz, A., Pacay, E., 2020. Is collective titling enough to protect forests? Evidence from Afro-descendant communities in the Colombian Pacific region. World Development 128, 2000–2005. https://doi.org/10.1016/j.worlddev.2019.104837

Watson, J.E.M., Evans, T., Venter, O., Williams, B., Tulloch, A., Stewart, C., Thompson, I., Ray, J.C., Murray, K., Salazar, A., McAlpine, C., Potapov, P., Walston, J., Robinson, J.G., Painter, M., Wilkie, D., Filardi, C., Laurance, W.F., Houghton, R.A., Maxwell, S., Grantham, H., Samper, C., Wang, S., Laestadius, L., Runting, R.K., Silva-Chávez, G.A., Ervin, J., Lindenmayer, D., 2018. The exceptional value of intact forest ecosystems. Nature Ecology and Evolution 2, 599–610. https://doi.org/10.1038/s41559-018-0490-x

Zhang, Y., West, P., Thakholi, L., Suryawanshi, K., Supuma, M., Straub, D., Sithole, S.S., Sharma, R., Schleicher, J., Ruli, B., Rodríguez-Rodríguez, D., Rasmussen, M.B., Ramenzoni, V.C., Qin, S., Pugley, D.D., Palfrey, R., Oldekop, J., Nuesiri, E.O., Nguyen, V.H.T., Ndam, N., Mungai, C., Milne, S., Mabele, M.B., Lucitante, S., Lucitante, H., Liljeblad, J., Kiwango, W.A., Kik, A., Jones, N., Johnson, M., Jarrett, C., James, R.S., Holmes, G., Gibson, L.N., Ghoddousi, A., Geldmann, J., Gebara, M.F., Edwards, T., Dressler, W.H., Douglas, L.R., Dimitrakopoulos, P.G., Davidov, V., Compaoré-Sawadogo, E.M.F.W., Collins, Y.A., Cepek, M., Burow, P.B., Brockington, D., Balinga, M.P.B., Austin, B.J., Astuti, R., Ampumuza, C., Agyei, F.K., 2023. Governance and Conservation Effectiveness in Protected Areas and

Indigenous and Locally Managed Areas. Annu. Rev. Environ. Resour. 48, 559–588. https://doi.org/10.1146/annurev-environ-112321-081348
Zou, Y., Crowther, T.W., Smith, G.R., Ma, H., Mo, L., Bialic-Murphy, L., Potapov, P., Gawecka, K.A., Xu, C., Negret, P.J., Lauber, T., Wu, Z., Rebindaine, D., Zohner, C.M., 2025. Fragmentation increased in over half of global forests from 2000 to 2020. Science 389.

Response to reviewers' comments

We have responded in **bold** to all the comments.

Reviewer #2 (Remarks to the Author):

1. Thank you for the extensive careful revisions. The manuscript is much stronger. See below for remaining questions and concerns.

We sincerely thank the reviewer for their careful evaluation of our revised manuscript and for the positive feedback. We greatly appreciate the time and effort invested in reviewing our work and are pleased that the revisions have strengthened the manuscript. Below, we respond to the remaining questions and concerns in detail.

Major comments:

2. Potential control sites: Lines 294-295 are the most critical in the entire paper, as the matching process for control sites will dramatically shape estimated treatment effects. Though the pool of potential control sites/pixels is probably clear in the minds of the authors, these potential control sites are still not clear to the reader. Please include one or more figures (e.g., real or cartoon map; flow diagram, etc) that illustrate the residual forests/governance mechanisms that are eligible for matching as control pixels.

We thank the reviewer for emphasizing the importance of clearly defining the pool of potential control sites. In response, we revised the Methods section (Lines 316-321) to explicitly clarify that potential control pixels were drawn from forested areas not assigned to any of the assessed governance mechanisms. We also acknowledge that additional governance arrangements with conservation potential exist in the Peruvian Amazon, but decided not to include them in the analysis because they were implemented after 2005 or lack consistent spatial information at the Amazon scale. The section now reads;

"Potential control pixels were drawn from forested areas that were not assigned to any of the assessed governance mechanisms. While additional governance mechanisms with conservation potential exist in the Peruvian Amazon, they were not included in the analysis because they were implemented after 2005 or lack consistent spatial information at the Amazon scale (Lagneaux et al., 2024) ***(Supplementary Table 2). A flow diagram of the process for the definition of the pool of control pixels is presented in Supplementary figure 4."***

Also following the reviewer's suggestion, we added a new supplementary flow diagram (Supplementary Fig. 4) illustrating the sequential process used to define the pool of potential treatment and control pixels, including exclusions of treatment areas, overlapping designations, post-2005 designations, and buffer zones. We also included a supplementary table summarizing the main residual governance mechanisms that could occur within the control pool, along with their general characteristics (Supplementary Table 2).

Together, these additions clarify the definition of control sites, transparently acknowledge residual governance complexity, and visually document the treatment and control selection process.

We have also included a paragraph in the Discussion (Lines 545–552) highlighting this as a caveat of the analysis;

"Our analysis comparing the effectiveness of potential OECMs preventing forest loss in the Peruvian Amazon provides an overview of the impact of these governance mechanisms avoiding deforestation and its associated carbon emissions, but there are some caveats. First, for this analysis we assessed the most widely distributed governance mechanisms in the Peruvian Amazon, but other governance mechanisms that could potentially be listed as OECMs, including private (Peruvian State, 2025a) ***and regional*** (Peruvian State, 2025b) ***conservation areas, were not included in our assessment. In future studies, it would be valuable to evaluate their impact on biodiversity conservation and assess their potential to be listed as OECMs."***

3. Spatial extent of land classifications used in the analysis: Please include a supplemental table that lists the total area (km2) and percentage of the study area (i.e., Peruvian Amazon) represented by each of the classifications used to identify or exclude potential treatment pixels and control pixels. In other words, how much land was within the buffer zones for each governance mechanism? How much land was within the "established after 2005" category for each mechanism? How much land was a viable treatment for each mechanism? How much was a potential control? Etc. It's hard to understand exactly what's going on in the analysis (and how to interpret the results) without this descriptive info.

We have now included this table as supplementary material. We mention it in lines 312-314;

"The amount (km²) and percentage of land excluded from the control and treatment areas is shown in Supplementary Table 1."

4. Lines 394-400 – Please clarify why the pixel-level results differ from the site-level results. Is there a disproportionate distribution of pixels to a small number of large sites that skews the analysis at the pixel level? Need to explain site level variation in efficacy… this is important both scientifically and from policy/practice standpoint, since "sites" are managed (not pixels). And if site size matters, then shouldn't this be captured within the matching methods?? If not, justify why not… b/c size (or distance to boundary of area-based governance system?) is either an explanatory variable or a matching variable.

We thank the reviewer for raising this important point regarding differences between pixel-level and site-level results.

The reasons for the divergence between pixel-level and site-level results are now explicitly explained in the Results section (Lines 431–444);

"When the results were aggregated at the site level, we found trends broadly consistent with the pixel-level analysis, with the exception of Indigenous Lands (ILs), which showed higher average forest loss per site in treatment areas than in controls (Supplementary Table 5; Fig. 4; Supplementary Fig. 10). This divergence reflects how observations are weighted in pixel versus site level summaries. Pixel-level analyses up-weight larger sites because they contain more pixels and therefore capture the overall governance mechanism impact on landscape-level forest loss trends, whereas site-level analyses weight all sites equally and better reflect variation in effectiveness at the scale at which management decisions are implemented. As a result, site-level averages are more strongly influenced by the performance of numerous small sites, while pixel-level results are driven by fewer large sites that contain a greater share of forest area. For ILs there is a higher number of smaller sites than larger ones (Supplementary Fig. 11) and those sites represent a higher proportion of the total pixels used in the analysis (Supplementary Fig. 12). These smaller sites tend to be ineffective preventing deforestation, so the mean avoided deforestation by site is strongly influenced by those small ineffective sites, despite larger sites tending to be effective in reducing deforestation (Fig. 4)."

We further elaborate on site-level variation in effectiveness in the Discussion (Lines 482–484), where we show that effectiveness varies substantially across sites and is associated with site size for several governance mechanisms;

"While our results show that different governance mechanisms in the Peruvian Amazon can reduce forest loss, we also found that conservation effectiveness varies substantially across sites within the same governance mechanism and that site size can be associated with this variation."

Regarding the inclusion of site size in the matching procedure, we note that matching was conducted at the pixel level using pixel-specific covariates associated with deforestation risk and intervention placement. Site size is a site-level attribute and therefore cannot be assigned to control pixels, which do not belong to a defined site. As such, site size cannot be included as a matching covariate without violating the logic of pixel-level counterfactual matching. Instead, we examine the role of site size by aggregating post-matching results at the site level, allowing us to assess how effectiveness varies with site size (Fig. 4).

While we agree that understanding how site size and other site-level characteristics shape conservation outcomes is a critical research frontier, a systematic analysis of these drivers is beyond the scope of this study. We now explicitly highlight this as a priority direction for future research in the Discussion in lines 484 to 491;

"Because sites are the operational units of management, understanding how site-level characteristics such as size, boundary configuration, accessibility, and governance capacity are associated with conservation outcomes is important for policy and practice. In this study, matching was conducted at the pixel level to control for spatial confounding in deforestation risk, which precludes the inclusion of site-level attributes such as size in the matching process. Further research examining how site-level characteristics relate to variation in effectiveness would improve understanding the conditions under which different governance mechanisms deliver conservation benefits."

Other comments

5. Abstract: Talk about findings for extractive governance systems, too?

We have now included the results for LCs and MCs in the abstract in lines 66 to 67;

"On the other hand, LCs and MCs showed a 13% and 24% increase in expected forest loss, respectively."

6. Introduction: highlight "conservation abandonment" generally as a challenge in conservation, as it affects all forms of conservation initiatives through both abdication of management responsibilities (e.g., paper parks) and rollbacks of formal protections (e.g., PADDD). See/cite new paper by Pienkowski et al on this. (Pienkowski, et al. Conservation abandonment is a policy blind spot. Nat Ecol Evol (2025). https://doi.org/10.1038/s41559-025-02910-5).

We thank the reviewer for bringing this into our attention. We have now added a paragraph in the introduction in lines 94 to 99 to acknowledge the importance of accounting for abandonment in conservation;

"Regardless of the type of area-based conservation measure implemented, however, conservation outcomes may erode over time through processes of conservation abandonment, including both the abdication of management responsibilities (e.g., "paper parks") and the reversal or weakening of formal protections, such as downgrading, downsizing, and degazettement. As such, conservation abandonment should be considered when planning conservation actions (Pienkowski et al., 2025)***."***

7. Probably makes sense to mention "paper parks" in this intro… and long-term financing for "permanence" in the discussion/conclusion.

We now mention paper parks as an example of the abdication of management responsibilities (lines 94 – 99);

"Regardless of the type of area-based conservation measure implemented, however, conservation outcomes may erode over time through processes of conservation abandonment, including both the abdication of management responsibilities (e.g., "paper parks") and the reversal or weakening of formal protections, such as downgrading, downsizing, and degazettement. As such, conservation abandonment should be considered when planning conservation actions (Pienkowski et al., 2025)***."***

We also now do reference to long term financing in the discussion (lines 579 – 581);

"In parallel, it is crucial to ensure long term financing and the development of mechanisms that address conservation abandonment to ensure that both OECMs and PAs deliver sustained conservation benefits over the long term (Pienkowski et al., 2025)***."***

8. Note that "reclassification" of PAs as OECMs is not PADDD. Reclassification is a change in conservation accounting and reporting; PADDD represents a substantive change in law/policy/regulations, with substantive impacts on the rules governing a site. (Reclassification may lead to PADDD, but they are procedurally and substantively different.)

We thank the reviewer for bringing this to our attention. We have revised the document to clearly distinguish between reclassification and PADDD. Specifically, we now differentiate between changes in conservation accounting and reporting (e.g., reclassification of sites as OECMs) and substantive legal or policy changes that alter governance rules (i.e., PADDD). We have removed any text suggesting that reclassification constitutes or implies PADDD, to avoid conflating procedural reporting changes with regulatory modifications that affect conservation outcomes. In addition, we have revised the introduction and discussion to focus on the broader concept of conservation abandonment, as suggested by the reviewer, to more appropriately frame risks to long-term conservation outcomes across governance mechanisms.

9. Probably makes sense to cite the original paper on PADDD (Mascia & Pailler, 2011, Conservation Letters 4:1.)

We have now included the citation as suggested by the reviewer.

10. Lines 353-364 – please report human pressure values for both sets of PAs (1-2 and 3-6) in the text.

We now report on all PA categories separately in this section. The section (lines 387 – 394) now reads:

"The average human footprint score within PAs and OECMS is 1.00 and 1.39, respectively (Supplementary Fig. 1a), compared to a global average of 6.06 (Sanderson et al., 2022)***. PAs in IUCN category I-II had lower average human footprint scores (0.52) than those in category III-VI (1.27), or those without a reported category (0.97; Supplementary Fig. 1a). Within individual OECMs, average human footprint levels vary widely, ranging from 0 (no observed human pressure) to above 6 (high pressure; Supplementary Fig 1b). OECMs have higher mean human pressure than PAs across all IUCN categories, with significant differences found between all categories (Supplementary Table 3), and larger differences seen between OECMS and PAs in IUCN category I-II (Supplementary Fig. 1a)."***

11. Lines 372-373: Clarify please. Is this true for PAs in proportional or absolute terms or both?

In this sentence, we were referring to proportional terms. We have clarified this now in the text. Now it reads;

"On the other hand, PAs showed the lowest yearly and cumulative proportional loss of forest between 2005 and 2021 (Fig. 4 b & c)."

12. Lines 382-84: Clarify please. Is there a difference or not (i.e., "similar")? Seems like the manuscript is saying both "yes" and "no".

We thank the reviewer for bringing this to our attention. We have revised the paragraph to improve clarity, and it now reads:

"Our results show that PAs, ILs and NTCs decrease forest loss compared to the matched control areas. PAs were the most effective in preventing forest loss with those in categories I and II having a higher effectiveness than those in categories III and VI (avoiding 60% and 49% of expected loss respectively)."

13. 405-6: MCs were "ineffective"? or did they accelerate deforestation?

We have now revised the paragraph for clarity. The paragraph now reads;

"In contrast, MCs in general increased forest loss compared to matched control areas (Fig. 4 b)."

14. Table 2 – report PA results both aggregated (as shown) and disaggregated into the subcategories used elsewhere in the manuscript (e.g., Fig 1; I-II, III-VI).

We now report on all PA categories in this table. As requested by the reviewer this table is now in supplementary material as Supplementary Table 4.

15. Figure 2. Unclear what "darker colours" are – they all look the same. To make this point, show both "darker" and "lighter" in the figure legend.

We thank the reviewer for bringing this to our attention. The darker colours in the figure (now Figure 1) do not represent additional governance classes; rather, they indicate areas that were forested in 2005, while the lighter colours represent areas without forest in 2005. In the previous version, the contrast between darker and lighter tones was not sufficiently clear. We have now revised the figure to improve the visual distinction between these colour tones and enhance readability. We have also updated the legend to clarify this distinction. The legend now reads:

"Map of the Peruvian Amazon showing the extent of Protected Areas, Indigenous Lands, Non-Timber Forest Product Concessions, Logging Concessions, and Mining Concessions included in the analysis. Darker shades indicate areas that were forested in 2005, while lighter shades represent areas without forest in 2005."

16. Figure 3 panels A, B, and C are hard to read and interpret, since each panel includes multiple labeled Y axes. Suggest splitting fig 3 into 2 figures – one figure with panels a, b, and c vertically arrayed and a second figure with panels D and E.

Based on the reviewer suggestion we have revised Figures 3 and 4 to improve clarity and readability, including restructuring the panels to reduce visual complexity and make the axes and results easier to interpret.

Reviewer #4 (Remarks to the Author):

Scientific research is relevant.
The proposed methodology is clear in each of its steps and provides sufficient detail for replicating the work in other areas of interest.
It presents its results adequately and illustrates its findings in the figures.
Its results are compared with recent research on the topic.
Regarding its conclusions, they highlight the importance of evaluating these governance mechanisms. Further research could be conducted on South America and how these mechanisms are effective in the Global South compared to other parts of the world.

We sincerely thank the reviewer for their positive evaluation of our manuscript. We appreciate the recognition of the relevance of the research, the clarity and reproducibility of the methodology, and the presentation of the results and figures. We are also pleased that the discussion and conclusions were viewed as well-supported and aligned with recent literature.
We agree that further comparative research in South America and across the Global South would be valuable and represents an important avenue for future work. In response, we have incorporated an additional paragraph in the Discussion (Lines 581 – 590) highlighting the need for comparative analyses across regions to better understand how governance mechanisms perform under different socio-political and ecological contexts;

"Building on this, further research is needed to better understand how the effectiveness of different governance mechanisms varies across regions, particularly within South America and more broadly across the Global South. Governance arrangements operate within distinct socio-political, economic, and ecological contexts, which may influence their conservation performance and long-term stability. Comparative analyses across countries and regions would help identify the institutional, financial, and socio-environmental conditions under which area-based conservation measures are most effective. Such work would not only improve understanding of regional variation in conservation outcomes, but also support more context-sensitive policy design and implementation, ensuring that governance mechanisms are tailored to local realities while contributing to global biodiversity and climate goals."

Reviewer #6 (Remarks to the Author):

The revised manuscript "Potential of different area-based governance mechanisms for achieving Global Biodiversity Framework goals" presents a global analysing of human footprint in protected areas and OECMs (as per WDPA database) and a region impact evaluation of multiple governance mechanisms avoided deforestation and carbon within the Peruvian Amazon. As a new reviewer I have largely tried to focus on whether the revisions successfully address the reviewer requests. The authors have addressed reviewer queries mostly but some further revisions are required in particular to address the substantive request around considering baseline trends before intervention dates. However, I did find that some of the new text introduced in response to reviewer 2 is problematic and ask for some further major revision to that text. I also note that I have major reservations around the inclusion of the global analysis and that my view is it detracts from the robust impact evaluation results. I encourage the authors to remove the analysis or move it solely to supplementary materials and to further address the reviewer 2 comments on the global results and consider my additional points. Lastly, I make line edit comments on the introduction which I found could be improved further.

We thank the reviewer for the careful and thorough evaluation of our revised manuscript and for the constructive feedback provided. We have responded in detail to each of the concerns raised (see full responses above).

In summary, we have: (1) strengthened the treatment–control design by explicitly demonstrating comparable pre-intervention trends using additional supplementary figures; (2) revised and clarified newly introduced text to avoid conceptual ambiguities, particularly regarding OECMs, PADDD, and reclassification; (3) improved the logical framing and clarity of the Introduction; and (4) addressed concerns regarding pseudo-replication and transparency in site size distributions through additional tables and figures.

Regarding the global analysis, we have carefully reconsidered its placement in response to the reviewer's reservations. To ensure that the manuscript remains focused on the more rigorous regional impact evaluation, we have moved the global human footprint analysis to the Supplementary Material and revised the main text accordingly to clarify its scope and limitations.

We believe these revisions substantially improve the clarity, focus, and rigor of the manuscript.

<u>New comments from myself presumed reviewer 3:</u>

Line 91 – the new text defining governance mechanisms is useful but doesn't flow well here. I'd suggest that you revise line 90 to better point out that other conservation tools/area based measures such as OECMs, ICCAs, etc are available in addition to PAs and that collectively these represent a subset of very many governance mechanisms potentially available to deliver on effective and equitable conservation. Then you can define governance mechanisms.

We thank the reviewer for this helpful suggestion. We have revised the paragraph in the Introduction to better situate OECMs, ICCAs, and other area-based measures within the

broader landscape of governance mechanisms before providing the definition. The revised text (Lines 90 – 101) now reads:

"Moreover, in some circumstances, the designation of a PA may not be appropriate or feasible (Negret et al., 2024)***, and other area-based conservation measures, such as OECMs, Indigenous and Community Conserved Areas (ICCAs), and other locally governed initiatives, may provide effective and equitable conservation outcomes*** (Cook, 2024; H. D. Jonas et al., 2024; Pathak Broome, 2024)***. Collectively, these represent a subset of the diverse governance mechanisms available to support conservation objectives*** (Maxwell et al., 2020)***. Regardless of the type of area-based conservation measure implemented, however, conservation outcomes may erode over time through processes of conservation abandonment, including both the abdication of management responsibilities (e.g., "paper parks") and the reversal or weakening of formal protections, such as downgrading, downsizing, and degazettement (DDD)*** (Golden Kroner et al., 2019; Mascia and Pailler, 2011)***. Conservation abandonment should therefore be considered when planning and evaluating conservation actions*** (Pienkowski et al., 2025)***. Here we define governance mechanisms as the system of laws, norms, rules, policies and practices that determine how landscapes and resources are managed and regulated in a spatially defined area*** (Zhang et al., 2023)***."***

Line 95 – line uses the term 'protection' however the term used in the GBF target is actually 'effectively conserved and managed' which is an important nuance as the target treats a diversity of governance mechanisms (protected areas, OECMs, and ICCAs) as equal rather than hierarchical. I suggest given scope of this study it is important to be careful in terminology here and to use more precise phrasing from the target.

We have now changed "protection" for "effective conservation and management" in this paragraph.

Line 105 – 107 – I find this new text around OECM DDD problematic – I suggest deleting this text. The imperative surely is to study in rigorous ways the outcomes of governance mechanisms and ideally through experimental approaches that enable credible comparisons of the governance mechanisms to be able to support the best mechanisms for a particular context to deliver on social and ecological goals. This study does that by designing comparative impact evaluations to study outcomes across 6 mechanisms. In doing so it reveals some mechanisms that decrease forest loss and some that increase forest loss and thus some that may contribute to conservation outcomes (and perhaps be aligned with OECM criteria) and some that are not. This should be the emphasis of the introduction – to set up the need for such credible evidence bases.

We have removed the paragraph as suggested. We have also revised the Introduction to more clearly articulate the study's objective and research question, emphasizing the importance of rigorous comparative impact evaluations to generate credible evidence on the effectiveness of different governance mechanisms in delivering conservation outcomes.

Line 108 - ILs aren't inherently OECMs - any one area owned and managed by Indigenous People's would have to be enrolled as an OECM under the OECM criteria. But equally that area may instead be listed as an ICCA. Why the emphasis here on IP and Communities and that they must inherently be enrolled as an OECM. Given this study conducts an impact evaluation to test forest outcomes across governance mechanisms I'd encourage the authors to consider a broader introductory framing emphasizing the very many useful and appropriate governance mechanisms that can deliver outcomes but that need NOT have to be declared as PA nor OECM but can still be recognized as contributing to conservation area based targets. It's useful to know which governance mechanisms deliver positive outcomes (as this study demonstrates0 and within these any one community or landholder could then choose via informed and continual consent to enrol their area as a particular recognized mechanism such as an OECM.

We have revised the paragraph in the Introduction to clarify that Indigenous Lands are not inherently OECMs and to adopt a broader framing of governance mechanisms. The revised text emphasizes that many governance arrangements, whether or not formally designated as PAs or OECMs, can deliver meaningful conservation outcomes, and that formal recognition is distinct from demonstrated ecological performance. This adjustment aligns the Introduction more closely with the impact evaluation focus of the study and avoids implying that Indigenous territories must be enrolled under any particular designation. The paragraph (lines 114 – 134) now reads;

"Sites recognized as OECMs can be governed through diverse mechanisms, but many governance arrangements that are not formally designated as PAs or OECMs may also deliver meaningful conservation outcomes (Cook, 2024; Harry D. Jonas et al., 2024)***. For example, land management by Indigenous Peoples, including Indigenous Lands (ILs) , has been shown, under suitable conditions, to be highly effective in limiting deforestation*** (Alejo et al., 2021; Brondízio et al., 2019; Sze et al., 2022; Vélez et al., 2020)***. A study in the Brazilian Amazon found that deforestation and burned areas inside ILs were two and four times lower than outside, respectively*** (Nepstad et al., 2006)**.**

Forest concessions, in which a government temporarily provides public forested land access, management and exclusion rights to non-government actors, represent another governance mechanism that can provide positive outcomes for biodiversity conservation while generating income for local communities (Tegegne et al., 2019)***. There are at least 122 million hectares of forest concessions in West and Central Africa, Latin America and Southeast Asia*** (Tegegne et al., 2019)***, with different uses ranging from industrial extraction, including timber extraction or logging (logging concessions; LCs)*** (Azevedo Ramos et al., 2015; Clark et al., 2009; Karsenty and Ferron, 2017)***, to non-timber uses such as agroforestry*** (Nunes et al., 2012)***, tourism and conservation*** (Schleicher, 2018)***, hereafter referred to as Non-Timber Forest Product Concessions (NTCs). The effectiveness of forest concessions in reducing deforestation varies depending on concession type and political context, but there is strong evidence of positive conservation outcomes under adequate management and supportive political circumstances*** (Azevedo Ramos et al., 2015; Radachowsky et al., 2012; Schleicher, 2018)***. Other governance mechanisms associated with positive conservation outcomes include sacred sites, military areas, private, urban and surfing reserves, and university research areas*** (Cook, 2024; Qin et al., 2024)***. Whether such sites are formally recognized as PAs, reported as OECMs, or remain under other designations, their conservation contribution ultimately depends on their ecological outcomes and governance performance."***

Line 135-140 - as a style preference I'd prefer the authors state what they sought to answer as a research question or aim rather than just telling me what they did. What is the pressing knowledge need that you seek to address? Presumably this paper seeks to quantify the effectiveness of a diversity of governance mechanisms in halting deforestation, and thus hopefully protecting biodiversity, and it does so in the Peruvian Amazon. This contributes an important empirical evidence base for understanding which governance mechanisms might be best placed in diverse contexts to deliver on the ultimate goals of halting extinctions and protecting biodiversity into the future. To do this it conducts impact evaluations of avoided deforestation comparing multiple governance mechanisms over the same temporal period, applying robust counter factual methods to account for covariates, and thus creates like for like impact estimates for these diverse mechanisms.

We thank the reviewer for this suggestion. We have now revised the paragraph in the Introduction to more clearly articulate the study's central research aim in response to the reviewer's comment. Specifically, we reframed the section to emphasize the pressing knowledge need, namely generating robust empirical evidence on the comparative effectiveness of diverse governance mechanisms in reducing forest loss and supporting biodiversity and climate goals. The revised paragraph (lines 149– 156) is provided below;

"Here, we aim to generate robust empirical evidence on the effectiveness of diverse governance mechanisms in reducing forest loss and associated carbon emissions, thereby informing which mechanisms are best positioned to contribute to biodiversity and climate goals. At the global scale, we assess the extent and intensity of human pressure within sites already listed as OECMs compared to PAs. Focusing on the Peruvian Amazon, we conduct comparative impact evaluations across multiple governance mechanisms using statistical matching to account for their non-random spatial distribution and underlying deforestation risk. Specifically, we quantify and compare the impacts of PAs, ILs, NTCs, LCs, and MCs on forest loss and associated carbon emissions between 2005 and 2021."

In addition to these new revisions from myself I summarize in the next section where, in my view, the authors responses to reviewer 2 have not been sufficient. I use the reviewer 2 exact requests and author responses to note what hasn't yet been addressed in full.

Reviewer 3 requests for further author revision to address Reviewer #2 comments:

Methods: Further clarification is required regarding treatment and control site selection.
While revisions have made some improvements to address reviewer 2 requests, main text methods remain unclear. At line 210 a concise statement at the very start of this methods section should articulate there are 6 possible interventions that are non overlapping spatially which they investigate. These are PA IUCN I-II, PA IUCN III-VI, IL, NTC, LC, MC. These should be defined in practical terms, logic - why they are relevant to study here – data selected, as well as theory of change in terms of why they would or would not be expected to result in changes to forest cover (as the outcome variable here) and longer term biodiversity.

We thank the reviewer for his suggestion. We revised the Methods section to explicitly clarify treatment and control pixels selection. The section (Lines 316-321) now reads;

"Potential control pixels were drawn from forested areas that were not assigned to any of the assessed governance mechanisms. While additional governance mechanisms with conservation potential exist in the Peruvian Amazon, they were not included in the analysis because they were implemented after 2005 or lack consistent spatial information at the Amazon scale (Lagneaux et al., 2024) ***(Supplementary Table 2). A flow diagram of the process for the definition of the pool of control pixels is presented in Supplementary figure 4."***

To improve transparency, we added a new supplementary flow diagram (Supplementary Fig. 4) illustrating the sequential process used to define the pool of potential control and treatment pixels, including exclusions of treatment areas, overlapping designations, post-2005 designations, and buffer zones. We also include a supplementary table summarizing the main residual governance mechanisms that may still be present within the control pool, along with their general characteristics (Supplementary Table 2).

Together, these additions clarify the definition of control and treatment sites, transparently acknowledge residual governance complexity, and visually document the control selection process.

Regarding the description of the governance mechanisms assessed, we agree that additional clarity was needed. We have revised the Introduction (Lines 114–139) to provide clearer definitions and more detailed descriptions of each governance mechanism evaluated in the study, including their characteristics, relevance to the analysis, and their potential influence on forest cover and biodiversity loss. While we acknowledge the importance of clearly defining the interventions, we expanded this discussion in the Introduction rather than the Methods section, as we consider the conceptual framing and description of governance mechanisms to be better situated there.

"Sites recognized as OECMs can be governed through diverse mechanisms, but many governance arrangements that are not formally designated as PAs or OECMs may also deliver meaningful conservation outcomes (Cook, 2024; Harry D. Jonas et al., 2024)***. For example, land management by Indigenous Peoples, including Indigenous Lands (ILs) , has been shown, under suitable conditions, to be highly effective in limiting deforestation*** (Alejo et al., 2021; Brondízio et al., 2019; Sze et al., 2022; Vélez et al., 2020)***. A study in the Brazilian Amazon found that deforestation and burned areas inside ILs were two and four times lower than outside, respectively*** (Nepstad et al., 2006)**.**

Forest concessions, in which a government temporarily provides public forested land access, management and exclusion rights to non-government actors, represent another governance mechanism that can provide positive outcomes for biodiversity conservation while generating income for local communities (Tegegne et al., 2019)***. There are at least 122 million hectares of forest concessions in West and Central Africa, Latin America and Southeast Asia*** (Tegegne et al., 2019)***, with different uses ranging from industrial extraction, including timber extraction or logging (logging concessions; LCs)*** (Azevedo Ramos et al., 2015; Clark et al., 2009; Karsenty and Ferron, 2017)***, to non-timber uses such as agroforestry*** (Nunes et al., 2012)***, tourism and conservation*** (Schleicher, 2018)***, hereafter referred to as Non-Timber***

Forest Product Concessions (NTCs). The effectiveness of forest concessions in reducing deforestation varies depending on concession type and political context, but there is strong evidence of positive conservation outcomes under adequate management and supportive political circumstances (Azevedo Ramos et al., 2015; Radachowsky et al., 2012; Schleicher, 2018)***. Other governance mechanisms associated with positive conservation outcomes include sacred sites, military areas, private, urban and surfing reserves, and university research areas*** (Cook, 2024; Qin et al., 2024)***. Whether such sites are formally recognized as PAs, reported as OECMs, or remain under other designations, their conservation contribution ultimately depends on their ecological outcomes and governance performance.***

In contrast, mining concessions (MC) are primarily established for extractive purposes and are generally associated with increased deforestation and habitat degradation (Caballero Espejo et al., 2018; Giljum et al., 2022)***. Such activities can lead to substantial forest loss and fragmentation, with consequent negative effects on biodiversity and ecosystem integrity*** (Sonter et al., 2018)***. These differences underscore that governance mechanisms vary considerably in their conservation outcomes, depending on their objectives and management context."***

We have also incorporated a concise statement clarifying the governance mechanisms evaluated in the study (Lines 149–156). Although the reviewer suggested placing this clarification at the start of the Methods section, we included it in the Introduction, where we now more clearly articulate the study's aims and scope, consistent with earlier reviewer feedback. The revised text explicitly states that we conduct comparative impact evaluations across six non-overlapping governance mechanisms, PA IUCN I–II, PA IUCN III–VI, ILs, NTCs, LCs, and MCs, and briefly explains the analytical focus of the study. The relevant paragraph is provided below.;

"Here, we aim to generate robust empirical evidence on the effectiveness of diverse governance mechanisms in reducing forest loss and associated carbon emissions, thereby informing which mechanisms are best positioned to contribute to biodiversity and climate goals. At the global scale, we assess the extent and intensity of human pressure within sites already listed as OECMs compared to PAs. Focusing on the Peruvian Amazon, we conduct comparative impact evaluations across multiple governance mechanisms using statistical matching to account for their non-random spatial distribution and underlying deforestation risk. Specifically, we quantify and compare the impacts of PAs, ILs, NTCs, LCs, and MCs on forest loss and associated carbon emissions between 2005 and 2021."

Section on treatments line 209 - MC are entirely absent from the methods section starting line 209 so should be detailed

We have now included a new paragraph with a detail description of MC in the introduction section (lines 135 – 139);

"In contrast, mining concessions (MC) are primarily established for extractive purposes and are generally associated with increased deforestation and habitat degradation (Caballero Espejo et al., 2018; Giljum et al., 2022)***. Such activities can lead to substantial forest loss and fragmentation, with consequent negative effects on biodiversity and ecosystem integrity*** (Sonter et al., 2018)***. These differences underscore that governance mechanisms vary***

considerably in their conservation outcomes, depending on their objectives and management context."

<u>Controls –</u>
The authors have omitted false controls and improved detail in the methods. However, the new text:

"We matched each treatment observation to a unique control observation to ensure each governance mechanism pixel was paired with only one control pixel that had not been matched previously. Matched pairs of pixels had to be within 0.2 SD of the propensity scores (Austin, 2011; Lunt, 2014). *Pixels could be reselected as control samples for each independent governance mechanism analysis."*

Is confusing. How can each governance mechanism pixel be paired with one control pixel that has **not** been matched previously but also allow for pixels to be **reselected as control samples for each independent governance mechanism.** The first sentence implies the pool of control pixels can only be used once, the second implies no replacement for individual matching experiments but allowing controls to be reselected across independent governance mechanisms analyses. Please clarify text.

We have restructured the paragraph to clarify the matching procedure and resolve the ambiguity identified by the reviewer. The revised text now clearly distinguishes between matching without replacement within each governance mechanism analysis and the reuse of the same overall control pool across independent analyses. The revised paragraph is provided below;

"For each governance mechanism, we conducted an independent matching analysis in which each treatment pixel was paired with a single control pixel from the pool of eligible controls. Matched pairs of pixels had to be within 0.2 SD of the propensity scores (Austin, 2011; Lunt, 2014)***. Matching was performed without replacement within each governance mechanism analysis, meaning that a control pixel could only be used once in that specific matching exercise. However, the same overall pool of eligible control pixels was used across the separate matching analyses conducted for each governance mechanism."***

Methods: As reviewer 2 noted, A control-intervention study design with matching is stronger than such a design without matching, but not as strong as before-after-control-intervention. Reviewer 2 requested that the authors show trends in outcome variables for the control and intervention pools of pixels for each analysis and to test whether these trends are parallel. This is a simple and essential analysis to build credibility in the analyses. Yet the authors haven't implemented it. Rather they included trend as a covariate. I'm not convinced that is methodologically appropriate and it requires further discussion around why it is in fact a reasonable covariate. But this is separate to testing that trend lines pre intervention are the same across the two samples. The authors should implement the recommended test to show parallel trends in outcome variables prior to the study period.

We agree that demonstrating comparable pre-intervention trends is essential to strengthen causal inference. In our matching framework, we explicitly incorporated

deforestation trends between 2000 and 2005 as covariates at both the local scale (1-km radius) and the broader landscape scale (10-km radius). By matching treatment and control pixels on these pre-intervention deforestation trends, alongside other confounding variables, we ensured that matched pixels exhibited similar deforestation trajectories prior to the study period (2005–2021).

To further demonstrate this, we have included three supplementary figures (Supplementary Figs. 6–8). Figure S6 presents cumulative deforestation trajectories over time, illustrating that treatment and control pixels followed similar trends prior to 2005. Figures S7 and S8 present empirical cumulative distribution functions (ECDFs) of pre-2005 deforestation at the local and landscape scales, before and after matching. Greater overlap between treatment and control curves indicates more similar baseline deforestation distributions. In the post-matching panels, the treatment and control curves closely overlap, demonstrating improved balance in pre-intervention deforestation trends. .

We have clarified this in the Methods section (Lines 364–369). The revised paragraph is provided below.

"By including deforestation trends between 2000 and 2005 at both the local (1-km radius) and landscape (10-km radius) scales as matching variables, we ensured that treatment and control pixels had similar patterns of forest loss before the study period began. In other words, matched pixels were already experiencing comparable deforestation trends prior to 2005. This improves confidence that differences observed after 2005 are attributable to governance mechanisms rather than pre-existing differences in forest loss patterns (Supplementary Figs. 6–8)."

4. Methods: lines 314+ - reviewer 2 noted that the methods raise issues of spatial autocorrelation and pseudo replication. The authors have revised and included methods to address spatial autocorrelation. However, the methods don't actually address pseudo replication (testing for Moran's I corrects for autocorrelation within a site but not that you may only have a small number of sites that are very large thus lending itself towards pseudo replication). The text line 314 "We explicitly accounted for spatial autocorrelation and pseudo-replication by estimating the residual spatial autocorrelation of these models (Moran's I) and then removing the pixels highly autocorrelated from the analysis." Should be revised to be "We explicitly accounted for spatial autocorrelation by estimating the residual spatial autocorrelation of these models (Moran's I) and then removing the pixels highly autocorrelated from the analysis.". The authors should report explicitly in a simple table the number, size and distribution of size of each intervention type. Some of this can be inferred in figure 5 but this isn't transparent enough. A reader should be able to rapidly refer to a table or numerical reporting of these details as they directly influence credibility of results given the likelihood of pseudo replication influencing some analyses and results.

We have revised the text as suggested to accurately reflect the methodological approach. The sentence now reads:

"We explicitly accounted for spatial autocorrelation by estimating the residual spatial autocorrelation of these models (Moran's I) and then removing the pixels highly autocorrelated from the analysis."

We have added additional supplementary material to clearly report the number and size distribution of intervention and control sites. Supplementary Table 1 provides the total area (km²) and percentage of the study area represented by each governance mechanism, including exclusions and the final area used for analysis.

We have also included two supplementary figures. Supplementary Figure 11 presents histograms of the number of sites across size classes for each governance mechanism, and Supplementary Figure 12 presents histograms of the number of pixels on the sites on a particular size range. Together, these additions provide a transparent summary of the number, size, and size distribution of intervention types.

11. Discussion. In response to reviewer requests the authors introduced this new paragraph for which I highlight text that I found to lack a level of precision needed to address complex and intertwined issues:
"At the global scale, our results show that while OECMs and PAs face considerable human pressure levels, they still have far lower human pressure than unprotected land. Previous work has shown that PAs face widespread levels of intense human pressure (Jones et al., 2018)*, but there is also evidence that they can reduce deforestation and habitat loss* (Sze et al., 2022)*, and contain higher biodiversity than unprotected areas* (Gray et al., 2016)*. Here, we showed that, on average, OECMs have similar human pressure levels to PAs, particularly those in IUCN categories III-VI. Less degraded ecosystems, such as those within many PAs and OECMS, support an exceptional confluence of globally significant environmental values relative to the ones that have experienced damaging human actions* (Watson et al., 2018)*, so both OECMs and PAs have strong potential to deliver positive conservation outcomes. However, delivering on this potential will require concerted actions to overcome the many challenges facing area-based conservation efforts, such as underfinancing and under staffing* (Coad et al., 2019; Lessmann et al., 2024)*, PADDD* (Forrest et al., 2015; Golden Kroner et al., 2019)*, and resource extraction* (Durán et al., 2013)*. Moreover, 42% of all reported OECMs were previously classified as PAs* (Cook et al., 2024)*, so a better understanding of the causes, consequences and conservation impact of such changes is needed. Importantly, our global analysis does not consider the non-random location of PAs and OECMs and how this affects the effectiveness of these conservation mechanisms preventing biodiversity loss, which should be a priority for future research at the global scale."*

I make comment for each highlighted text item below:

they still have far lower human pressure than unprotected land. – include a reference please. You don't actually show this in your analysis as you only summarize HFI for PA and OECM not areas that are neither of these area based measures.

We thank the reviewer for this comment. We recognize that the original wording was unclear and have revised the sentence in Lines 387–388 to improve clarity. We have also

added a reference for the global mean human pressure values. The revised sentence now reads:

'The average HFP score within PAs and OECMS is 1.00 and 1.39, respectively (Supplementary Fig. 1a), compared to a global average of 6.06 (Sanderson et al., 2022)***.'***

OECMs have similar human pressure levels to PAs, particularly those in IUCN categories III-VI. – You present a box plot with averages and scatter plot of points but you don't actually test for differences in distributions – you are making this conclusion based on a visual inspection presumably that the average for OECM is closest to PA III-VI and scatters are similar-ish? This analysis in general is highly descriptive without clear connection to your more rigorous impact evaluation. If you are including it needs to be elevated in rigour and clarified in purpose. How does a single time step summary of average human footprint relate to then a n impact evaluation for avoided deforestation across multiple mechanisms. I'd argue it does not. The measures of outcome are not quantitatively related (and correlation between HFI and deforestation does not make for a sound reasoning particularly when there are global deforestation layers that could be used instead if a global summary analysis was essential for this study). The mechanisms are not related other than tangentially in that the authors are seeking to make the case that some governance mechanisms tested in the Peruvian amazon case study may be appropriate for consideration as OECM and they then test for impacts across these and PAs. The geographic distribution is non overlapping -the global results don't overlap with Peru as Peru does not have OECM in the database as shown in the map. I would argue this global analysis detracts from the study and would suggest it should be removed. If the authors disagree they'd need to revise the text to create a far more logical rationale of research questions to link the analyse and improve their rigour.

Thank you for raising these important points. It is worth noting that this global analysis was added to the paper in response to a request from another reviewer. However, we acknowledge that its presentation and explanation required further refinement. As requested by the reviewer, we have moved the tables and figures of the results of the global analysis to the Supplementary Material and conducted additional analyses to strengthen its statistical rigor. We have now conducted a Wilcoxon rank sum test to determine the significance of differences between mean human pressure values for OECMS and PAs of different IUCN categories. We selected this nonparametric test because the data did not satisfy the normality assumption required for a one-way ANOVA. Additional text has been added to the Methods section (Lines 227–231) to explain this new analysis:

"To examine differences in human pressure levels within OECMs and PAs, we conducted Wilcoxon rank-sum tests to assess the significance of differences in mean human footprint values across OECM and PA categories. OECM/PA category was treated as the predictor variable and mean human footprint as the response variable. The Wilcoxon rank-sum test, a nonparametric alternative to a one-way ANOVA, was used because the data did not meet the normality assumption required for parametric analysis."

We do believe that this analysis adds important contextual information at a global scale on the level of human pressure within OECMs and Protected Areas. There is no formal relationship between the global and Peruvian analyses, but understanding the overall

levels of human pressure within OECMs vs PAs does provide the reader with a more comprehensive understanding of the current status of land within these conservation areas. The reason we used human pressure data rather than global deforestation layers as suggested by the reviewer, was that deforestation data present challenges for a global assessment as many PAs/OECMS do not contain forest. The reviewer also states that there is no geographical overlap between the global and Peruvian analysis. This is incorrect, as there are OECMs in Peru which we included in the analysis but due to their small size they are difficult to visualise in the global map shown in Supplementary Figure 1. We have added explanation of this in the figure caption.

Moreover, 42% of all reported OECMs were previously classified as PAs (Cook et al., 2024)***, so a better understanding of the causes, consequences and conservation impact of such changes is needed.*** – due to the preceding sentence emphasizing PADDD, this text reads as though the OECMS previously classified as PAs now reclassified as OECMs have experienced a PADDD (downgrade) event. Was this the intent by the authors? OECMs are not less than PAs – they are designed to be seen as equivalent not hierarchical in order or preference. This study should be developing evidence for which potential governance mechanisms might deliver equivalent outcomes. I'd suggest this sentence and context should be removed or revised to avoid this implication. A counter argument of a negative impact from reclassification is that there were be positive outcomes for people and nature - A change from PA to OECM now that countries can report OECMs could be seen as a good thing – we know have terminology that is inclusive and appropriate for describing particular governance mechanisms. Perhaps this is a PA upgrade?

We have revised the document to clearly distinguish between reclassification and PADDD. In particular, the Discussion paragraph referring to the proportion of OECMs previously classified as PAs has been modified to remove any implication that such reclassification constitutes or implies a PADDD event. We now explicitly differentiate between changes in conservation accounting and reporting (e.g., reclassification of sites as OECMs) and substantive legal or policy changes that alter governance rules (i.e., PADDD). We have removed any wording suggesting that reclassification represents a weakening of protection, to avoid confusing reporting changes with actual changes in legal status or management on the ground. In addition, we have revised the Introduction and Discussion to focus more appropriately on the broader concept of conservation abandonment, as suggested in other reviewer comments, and to ensure that OECMs are not presented as hierarchical or subordinate to PAs. The revised paragraph (Lines 460–472) is provided below;

"However, delivering on this potential will require concerted actions to overcome the many challenges facing area-based conservation efforts, such as underfinancing and understaffing (Coad et al., 2019; Lessmann et al., 2024)***, PADDD*** (Forrest et al., 2015; Golden Kroner et al., 2019)***, conservation abandonment*** (Pienkowski et al., 2025)***, and resource extraction*** (Durán et al., 2013)***. In addition, recent work shows that 42% of reported OECMs were previously classified as PAs***(Cook et al., 2024)***. These changes reflect shifts in how sites are reported and categorized within international conservation frameworks, rather than necessarily changes in their legal protection or management. A clearer understanding of the implications of such reporting changes for conservation accountability and outcome***

assessment is therefore needed. Importantly, our global analysis does not assess the legal or governance changes associated with site reclassification, but instead evaluates human pressure within areas currently reported as PAs or OECMs. It also does not account for the non-random spatial location of these areas and how this may influence their effectiveness in preventing biodiversity loss. Addressing these limitations should be a priority for future research at the global scale."

Related to this point. Reviewer 2 comment on (previous) Lines 92-93. Note that Cook et al (OneEarth 7(3): 412-430) find that many protected areas around the world that have been reclassified as OECMs, now that there is a new accounting system that will allow governments to get credit for them. This observation should be considered and addressed with respect to the global analysis of human pressures within OECMs and PAs.

IN response to this the authors state:

We thank the reviewer for pointing this out. We have now included an additional paragraph in the introduction in lines 101 to 107 acknowledging this;

"More than 1.9 million km² of the Earth's surface has already been reported to the World Database on OECMs (UNEP-WCMC and IUCN, 2025)*, representing approximately 1% of total terrestrial area (Fig. 1). Two thirds of this area is governed by federal or national agencies* (Jonas et al., 2024)*. With OECMs rapidly gaining attention and traction in conservation policy, a clear understanding of the effectiveness of various OECM designations as conservation mechanisms is needed* (Jonas et al., 2024)*. It is also important to improve our understanding of the causes and consequences of PAs and OECMs downgrading, downsizing, and degazettement, given increasing reports of PAs being reclassified as OECMs* (Cook et al., 2024)*."*

However as with the discussion text – this introduction text clearly insinuates that OECMs reclassified to PAs are equivalent to PADDD events. Please revise. The reviewer 2 comment was instead (in my view) asking the authors to recognize that they have conducted a single time step analysis of averaging HFI in area based measures attributing 'likeness' of OECMs and PA classes but without recognizing that actually 42% of OECMS were in fact in PA classifications previously. What does this reclassification thus mean for a conclusion drawn that OECMs are 'like' PAs in terms of their human footprint profiles. Simply put this issue goes to the root failure of the simple summary analysis. Again, I feel the global analysis is too simplistic to add value. If it remains the authors would need to recognize and confront some of the problems with this simplistic summary of the severe limitations in some of the inferences they attempt to make from it.

We have revised the document to clearly distinguish between reclassification and PADDD. Specifically, we now differentiate between changes in conservation reporting (e.g., listing sites as OECMs) and actual legal or policy changes that weaken or alter protection (i.e., PADDD, conservation abandonment). We have removed any wording suggesting that reclassification constitutes or implies PADDD, to avoid confusing reporting changes with real changes in protection on the ground. In addition, we revised the Introduction and

Discussion to focus more explicitly on the need for rigorous comparative impact evaluations of governance mechanisms, rather than linking reclassification to PADDD events.

In terms of our global analysis comparing human pressure levels in OECMS and PAs, it is again important to note that this section was added in response to requests from another reviewer. That reviewer felt a global analysis would add helpful context for the Peruvian case study, and we believe that with the improvements outlined above it does so. To our knowledge, there have been no published assessments of human pressure across both OECMs and PAs, and so we believe this analysis adds context that could not be found elsewhere in the literature. We do not claim to assess the effectiveness of PAs or OECMs over time, nor through comparison to control sites. Rather, we assess the current state of human pressure within sites designated as PAs or OECMS at the global scale, to help contextualise the reader as to the state of land within these different conservation mechanisms. We have moved the figures and tables to the supplementary material as requested, and also now explicitly highlight the limitations of this analysis on lines 467-472, which reads:

"Importantly, our global analysis does not assess the legal or governance changes associated with site reclassification, but instead evaluates human pressure within areas currently reported as PAs or OECMs. It also does not account for the non-random spatial location of these areas and how this may influence their effectiveness in preventing biodiversity loss. Addressing these limitations should be a priority for future research at the global scale."

References:

Alejo, C., Meyer, C., Walker, W.S., Gorelik, S.R., Josse, C., Aragon-Osejo, J.L., Rios, S., Augusto, C., Llanos, A., Coomes, O.T., Potvin, C., 2021. Are indigenous territories effective natural climate solutions? A neotropical analysis using matching methods and geographic discontinuity designs. PLoS ONE 16. https://doi.org/10.1371/journal.pone.0245110

Austin, P.C., 2011. Optimal caliper widths for propensity-score matching when estimating differences in means and differences in proportions in observational studies. Pharmaceutical Statistics 10, 150–161. https://doi.org/10.1002/pst.433

Azevedo Ramos, C., Macedo Silva, J.N., Merry, F., 2015. The evolution of Brazilian forest concessions. Elementa 1–8. https://doi.org/10.12952/journal.elementa.000048

Brondízio, E.S., Settele, J., Díaz, S., Ngo, H.T. (Eds.), 2019. The global assessment report of the intergovernmental science-policy platform on biodiversity and ecosystem services. Intergovernmental Science-Policy Platform on Biodiversity and Ecosystem Services (IPBES), Bonn.

Caballero Espejo, J., Messinger, M., Román-Dañobeytia, F., Ascorra, C., Fernandez, L., Silman, M., 2018. Deforestation and Forest Degradation Due to Gold Mining in the Peruvian Amazon: A 34-Year Perspective. Remote Sensing 10, 1903. https://doi.org/10.3390/rs10121903

Clark, C.J., Poulsen, J.R., Malonga, R., Elkan, P.W., 2009. Logging concessions can extend the conservation estate for central African tropical forests. Conservation Biology 23, 1281–1293. https://doi.org/10.1111/j.1523-1739.2009.01243.x

Coad, L., Watson, J.E., Geldmann, J., Burgess, N.D., Leverington, F., Hockings, M., Knights, K., Di Marco, M., 2019. Widespread shortfalls in protected area resourcing undermine

efforts to conserve biodiversity. Frontiers in Ecol & Environ 17, 259–264. https://doi.org/10.1002/fee.2042
Cook, C.N., 2024. Diverse approaches to protecting biodiversity: The different conservation measures discussed as possible other effective area-based conservation measures. Conservation Letters. e13027. https://doi.org/10.1111/conl.13027
Cook, C.N., Lemieux, C.J., Golden Kroner, R., 2024. Increasing transparency and accountability in global protected and conserved area reporting. One Earth 7, 421–430. https://doi.org/10.1016/j.oneear.2024.01.022
Durán, A.P., Rauch, J., Gaston, K.J., 2013. Global spatial coincidence between protected areas and metal mining activities. Biological Conservation 160, 272–278. https://doi.org/10.1016/j.biocon.2013.02.003
Forrest, J.L., Mascia, M.B., Pailler, S., Abidin, S.Z., Araujo, M.D., Krithivasan, R., Riveros, J.C., 2015. Tropical Deforestation and Carbon Emissions from Protected Area Downgrading, Downsizing, and Degazettement (PADDD). Conservation Letters 8, 153–161. https://doi.org/10.1111/conl.12144
Giljum, S., Maus, V., Kuschnig, N., Luckeneder, S., Tost, M., Sonter, L.J., Bebbington, A.J., 2022. A pantropical assessment of deforestation caused by industrial mining. Proceedings of the National Academy of Sciences of the United States of America 119, 1–7. https://doi.org/10.1073/pnas.2118273119
Golden Kroner, R.E., Qin, S., Cook, C.N., Krithivasan, R., Pack, S.M., Bonilla, O.D., Cort-Kansinally, K.A., Coutinho, B., Feng, M., Martínez Garcia, M.I., He, Y., Kennedy, C.J., Lebreton, C., Ledezma, J.C., Lovejoy, T.E., Luther, D.A., Parmanand, Y., Ruíz-Agudelo, C.A., Yerena, E., Morón Zambrano, V., Mascia, M.B., 2019. The uncertain future of protected lands and waters. Science 364, 881–886. https://doi.org/10.1126/science.aau5525
Jonas, Harry D., Bingham, H.C., Bennett, N.J., Woodley, S., Zlatanova, R., Howland, E., Belle, E., Upton, J., Gottlieb, B., Kamath, V., Lessmann, J., Delli, G., Dubois, G., Ahmadia, G., Claudet, J., Cook, C., Deza, J., Grorud-Colvert, K., Gurney, G., Lemieux, C.J., Ruiz, L., 2024. Global status and emerging contribution of other effective area-based conservation measures (OECMs) towards the ‘30x30’ biodiversity Target 3. Front. Conserv. Sci. 5, 1447434. https://doi.org/10.3389/fcosc.2024.1447434
Jonas, H. D., Wood, P., Woodley, S. (Eds.), 2024. Guidance on other effective area-based conservation measures (OECMs). IUCN, International Union for Conservation of Nature. https://doi.org/10.2305/LAAW4624
Karsenty, A., Ferron, C., 2017. Recent evolutions of forest concessions status and dynamics in Central Africa. International Forestry Review 19, 10–26. https://doi.org/10.1505/146554817822295957
Lagneaux, E.G., Quaedvlieg, J., Sabogal, D., Jansen, M., 2024. Understanding the diversity of private conservation in the Peruvian Amazon. Conservat Sci and Prac e13228. https://doi.org/10.1111/csp2.13228
Lessmann, J., Geldmann, J., Fajardo, J., Marquet, P.A., 2024. The role of funding in the performance of Latin America’s protected areas. Proc. Natl. Acad. Sci. U.S.A. 121, e2307521121. https://doi.org/10.1073/pnas.2307521121
Lunt, M., 2014. Selecting an Appropriate Caliper Can Be Essential for Achieving Good Balance With Propensity Score Matching. American Journal of Epidemiology 179, 226–235. https://doi.org/10.1093/aje/kwt212
Mascia, M.B., Pailler, S., 2011. Protected area downgrading, downsizing, and degazettement (PADDD) and its conservation implications. Conservation Letters 4, 9–20. https://doi.org/10.1111/j.1755-263X.2010.00147.x
Maxwell, S.L., Cazalis, V., Dudley, N., Hoffmann, M., Rodrigues, A.S.L., Stolton, S., Visconti, P., Woodley, S., Kingston, N., Lewis, E., Maron, M., Strassburg, B.B.N., Wenger, A., Jonas, H.D., Venter, O., Watson, J.E.M., 2020. Area-based conservation in the twenty-first century. Nature 586, 217–227. https://doi.org/10.1038/s41586-020-2773-z

Negret, P.J., Venegas, R., Sonter, L.J., Possingham, H.P., Maron, M., 2024. Conservation planning for retention, not just protection. Global Change Biology 30, e17211. https://doi.org/10.1111/gcb.17211
Nepstad, D., Schwartzman, S., Bamberger, B., Santilli, M., Ray, D., Schlesinger, P., Lefebvre, P., Alencar, A., Prinz, E., Fiske, G., Rolla, A., 2006. Inhibition of Amazon Deforestation and Fire by Parks and Indigenous Lands. Conservation Biology 20, 65–73. https://doi.org/10.1111/j.1523-1739.2006.00351.x
Nunes, F., Soares-Filho, B., Giudice, R., Rodrigues, H., Bowman, M., Silvestrini, R., Mendoza, E., 2012. Economic benefits of forest conservation: Assessing the potential rents from Brazil nut concessions in Madre de Dios, Peru, to channel REDD+ investments. Environmental Conservation 39, 132–143. https://doi.org/10.1017/S0376892911000671
Pathak Broome, N., 2024. Recognising territories and areas conserved by Indigenous peoples and local communities (ICCAs) overlapped by protected areas. IUCN, International Union for Conservation of Nature. https://doi.org/10.2305/RSLY2962
Peruvian State, 2025a. Áreas de Conservación Privada (ACP) [WWW Document]. National Open Data Platform. URL https://www.datosabiertos.gob.pe/dataset/%C3%A1reas-de-conservaci%C3%B3n-privada-acp
Peruvian State, 2025b. Áreas de Conservacion Regional (ACR) [WWW Document]. National Open Data Platform. URL https://www.datosabiertos.gob.pe/dataset/%C3%A1reas-de-conservacion-regional-acr
Pienkowski, T., Clark, M., Mascia, M.B., Rivera-Hechem, M.I., Gelcich, S., Cook, C.N., Watrobska, C.M., Jagadish, A., Mills, M., 2025. Conservation abandonment is a policy blind spot. Nat Ecol Evol 10, 14–17. https://doi.org/10.1038/s41559-025-02910-5
Qin, S., He, Y., Golden Kroner, R.E., Shrestha, S., Henriques Coutinho, B., Karmann, M., Ledezma, J.C., Martinez, C., Morón-Zambrano, V., Ulloa, R., Yerena, E., Bernard, C., Bull, J.W., Mendoza, E., De Pracontal, N., Reytar, K., Veit, P., Olsson, E., Matallana-Tobón, C.L., Wily, L.A., Mascia, M.B., 2024. An inclusive, empirically grounded inventory facilitates recognition of diverse area-based conservation of nature. One Earth S2590332224001349. https://doi.org/10.1016/j.oneear.2024.03.005
Radachowsky, J., Ramos, V.H., McNab, R., Baur, E.H., Kazakov, N., 2012. Forest concessions in the Maya Biosphere Reserve, Guatemala: A decade later. Forest Ecology and Management 268, 18–28. https://doi.org/10.1016/j.foreco.2011.08.043
Sanderson, E., Fisher, K., Robinson, N., Sampson, D., Duncan, A., Royte, L., 2022. The March of the Human Footprint. https://doi.org/10.32942/OSF.IO/D7RH6
Schleicher, J., 2018. The environmental and social impacts of protected areas and conservation concessions in South America. Current Opinion in Environmental Sustainability 32, 1–8. https://doi.org/10.1016/j.cosust.2018.01.001
Sonter, L.J., Ali, S.H., Watson, J.E.M., 2018. Mining and biodiversity: Key issues and research needs in conservation science. Proceedings of the Royal Society B: Biological Sciences 285. https://doi.org/10.1098/rspb.2018.1926
Sze, J.S., Carrasco, L.R., Childs, D., Edwards, D.P., 2022. Reduced deforestation and degradation in Indigenous Lands pan-tropically. Nature Sustainability 5, 123–130. https://doi.org/10.1038/s41893-021-00815-2
Tegegne, Y.T., Cramm, M., Van Brusselen, J., Linhares-Juvenal, T., 2019. Forest concessions and the United Nations sustainable development goals: Potentials, challenges and ways forward. Forests 10, 1–21. https://doi.org/10.3390/f10010045
Vélez, M.A., Robalino, J., Cardenas, J.C., Paz, A., Pacay, E., 2020. Is collective titling enough to protect forests? Evidence from Afro-descendant communities in the Colombian Pacific region. World Development 128, 2000–2005. https://doi.org/10.1016/j.worlddev.2019.104837
Zhang, Y., West, P., Thakholi, L., Suryawanshi, K., Supuma, M., Straub, D., Sithole, S.S., Sharma, R., Schleicher, J., Ruli, B., Rodríguez-Rodríguez, D., Rasmussen, M.B., Ramenzoni, V.C., Qin, S., Pugley, D.D., Palfrey, R., Oldekop, J., Nuesiri, E.O.,

Nguyen, V.H.T., Ndam, N., Mungai, C., Milne, S., Mabele, M.B., Lucitante, S., Lucitante, H., Liljeblad, J., Kiwango, W.A., Kik, A., Jones, N., Johnson, M., Jarrett, C., James, R.S., Holmes, G., Gibson, L.N., Ghoddousi, A., Geldmann, J., Gebara, M.F., Edwards, T., Dressler, W.H., Douglas, L.R., Dimitrakopoulos, P.G., Davidov, V., Compaoré-Sawadogo, E.M.F.W., Collins, Y.A., Cepek, M., Burow, P.B., Brockington, D., Balinga, M.P.B., Austin, B.J., Astuti, R., Ampumuza, C., Agyei, F.K., 2023. Governance and Conservation Effectiveness in Protected Areas and Indigenous and Locally Managed Areas. Annu. Rev. Environ. Resour. 48, 559–588. https://doi.org/10.1146/annurev-environ-112321-081348

b UNIVERSITÄT BERN

WYSS ACADEMY FOR NATURE

<u>Response to reviewers' comments</u>

We have responded in **bold** to all the comments.

<u>Reviewer #6 (Remarks to the Author):</u>

1. Thank you for your further revisions. You have addressed my substantive comments well and navigated at times competing reviewer demands. The manuscript is improved but retains it's core messages well. I did notice a few potential typographical errors that the authors may wish to attend to.

We thank the reviewer for their positive assessment of the revised manuscript and for acknowledging our efforts to address the substantive comments and reconcile the different reviewer perspectives. We have carefully reviewed the manuscript and corrected typographical errors throughout the text to improve clarity and readability.

2. Line 87 - swapping between PAs (acronym) and spelled out protected areas - at times you swap between these or mix. Sentence on line 87 is a good example of this. Starts with "One third of PAs globally" and ends with "and 3% of forest within protected areas". I'd suggest trying to search text and clean up for consistency to make for a tighter read.

We thank the reviewer for this helpful suggestion. We have revised the manuscript to ensure consistent use of terminology throughout. Specifically, after the first definition of "protected areas (PAs)", we now use the acronym "PAs" consistently across the text. The sentence highlighted by the reviewer, along with other instances, has been corrected accordingly to improve clarity and readability.

3. Line 96-101 - this new text reads well but doesn't flow (in my opinion) well in this paragraph. I'd suggest this text might work better perhaps at line 116 and with an additional bridging sentence to frame how you will address here an understanding of relative effectiveness of governance mechanisms (that might potentially be aligned with OECM criteria).

We thank the reviewer for this constructive suggestion. We have removed the paragraph from lines 96–101 and relocated it to lines 109–120, as recommended. In doing so, we have revised the surrounding text and added a bridging sentence to ensure a clearer connection with the narrative in this section, particularly in framing the assessment of the relative effectiveness of different governance mechanisms in the context of OECMs. The revised paragraph now reads:

"Beyond the formal designation of area-based governance mechanisms, an important challenge lies in understanding how effectively sites under different governance mechanisms deliver conservation outcomes over time. Regardless of the type of area-based governance mechanism, conservation outcomes may erode through processes of conservation abandonment, including both the abdication of management responsibilities (e.g., "paper parks") and the reversal or weakening of formal protections, such as downgrading, downsizing, and degazettement [30,31]. Conservation abandonment should therefore be explicitly considered when planning and evaluating conservation actions [32]. These dynamics highlight the need to assess not only the extent but also the relative effectiveness of different

governance mechanisms, particularly in the context of emerging frameworks such as OECMs, which emphasize sustained and demonstrable biodiversity outcomes[20,21,29]. In this context, understanding how sites under different governance mechanisms contribute to conservation outcomes across different settings becomes particularly important."

4. Line 100 - you introduce the term downgrading, downsizing, and degazettement (DDD) as one part of abandonment but then in discussion revert back to just referencing DDD. I find the use of DDD instead of full words very distracting. Although I am very familiar with PADDD, encountering DDD as an acronym within sentences made communication disjointed. Given your introduction defines abandonment and groups DDD within that definition I suggest in remainder of text replacing DDD with 'abandonment'.

We thank the reviewer for this helpful suggestion. We have revised the manuscript to replace all mentions of "DDD" with "abandonment" throughout the text, following its definition in the Introduction, to improve clarity and readability.

5. Table s4 and Figure S1A - I found it hard to understand how these number match -given a much larger % of OECM area is in low pressure (~71%) relative to IUCN class III - VI (46%) I was expecting the box plots of visually have a clearer pattern of this density of points in lower class for OECMs and an equivalent or lower mean for OECM relative to multiple use PAs but Figure S1A seems to portray the opposite. Double check numbers and perhaps add some text in legends for clarity and include the mean HF values in Table S4 so a reader can at least verify across figures that the mean in the table matches the mean in the box plots.

Thank you for raising this point, we agree it was counterintuitive. The figures are correct, but showing different things. Figure S1A represents mean HFP values within each individual OECM and does not consider the area of land within each site. Table S4 measures the total area of land within OECMs under different human pressure categories. The reason there is so much land under low pressure in Table S4 is because of a few extremely large OECMs that have very low human pressure. We have added a new supplementary figure highlighting the relationship between OECM area and human pressure, highlighting these very large sites under low human pressure. We have also added text to the results section explaining this, and also to the captions of these figures.

6. Figure S4 - does a good job of complementing text methods. I did still find it hard to follow the volume of pixels in each of these stages kept or excluded and personally would have found it helpful to add that in so it's easier to then read results alongside this. However, as a methods workflow this figure does the job needed

We thank the reviewer for this helpful suggestion. To improve clarity regarding the number of pixels retained and excluded at each stage of the workflow, we have included the corresponding number of pixels for each step in Table S1, which complements Figure S4 and facilitates interpretation of the results.

The revised manuscript “Potential of different area-based governance mechanisms for achieving Global Biodiversity Framework goals” presents a global analysing of human footprint in protected areas and OECMs (as per WDPA database) and a region impact evaluation of multiple governance mechanisms avoided deforestation and carbon within the Peruvian Amazon. As a new reviewer I have largely tried to focus on whether the revisions successfully address the reviewer requests. The authors have addressed reviewer queries mostly but some further revisions are required in particular to address the substantive request around considering baseline trends before intervention dates. However, I did find that some of the new text introduced in response to reviewer 2 is problematic and ask for some further major revision to that text. I also note that I have major reservations around the inclusion of the global analysis and that my view is it detracts from the robust impact evaluation results. I encourage the authors to remove the analysis or move it solely to supplementary materials and to further address the reviewer 2 comments on the global results and consider my additional points. Lastly, I make line edit comments on the introduction which I found could be improved further.

New comments from myself presumed reviewer 3:

Line 91 – the new text defining governance mechanisms is useful but doesn’t flow well here. I’d suggest that you revise line 90 to better point out that other conservation tools/area based measures such as OECMs, ICCAs, etc are available in addition to PAs and that collectively these represent a subset of very many governance mechanisms potentially available to deliver on effective and equitable conservation. Then you can define governance mechanisms.

Line 95 – line uses the term ‘protection’ however the term used in the GBF target is actually 'effectively conserved and managed' which is an important nuance as the target treats a diversity of governance mechanisms (protected areas, OECMs, and ICCAs) as equal rather than hierarchical. I suggest given scope of this study it is important to be careful in terminology here and to use more precise phrasing from the target.

Line 105 – 107 – I find this new text around OECM DDD problematic – I suggest deleting this text. The imperative surely is to study in rigorous ways the outcomes of governance mechanisms and ideally through experimental approaches that enable credible comparisons of the governance mechanisms to be able to support the best mechanisms for a particular context to deliver on social and ecological goals. This study does that by designing comparative impact evaluations to study outcomes across 6 mechanisms. In doing so it reveals some mechanisms that decrease forest loss and some that increase forest loss and thus some that may contribute to conservation outcomes (and perhaps be aligned with OECM criteria) and some that are not. This should be the emphasis of the introduction – to set up the need for such credible evidence bases.

Line 108 - ILs aren't inherently OECMs - any one area owned and managed by Indigenous People's would have to be enrolled as an OECM under the OECM criteria. But equally that area may instead be listed as an ICCA. Why the emphasis here on IP and Communities and that they must inherently be enrolled as an OECM. Given this study conducts an impact evaluation to test forest outcomes across governance mechanisms I’d encourage the authors to consider a broader introductory framing emphasizing the very many useful and appropriate governance mechanisms that can deliver outcomes but that need NOT have to be declared as PA nor OECM but can still be recognized as contributing to conservation area based targets. It’s useful to know which governance mechanisms deliver positive outcomes (as this study demonstrates0 and within these any one community or landholder could then choose via informed and continual consent to enrol their area as a particular recognized mechanism such as an OECM.

Line 135-140 - as a style preference I'd prefer the authors state what they sought to answer as a research question or aim rather than just telling me what they did. What is the pressing knowledge need that you seek to address? Presumably this paper seeks to quantify the effectiveness of a diversity of governance mechanisms in halting deforestation, and thus hopefully protecting biodiversity, and it does so in the Peruvian Amazon. This contributes an important empirical evidence base for understanding which governance mechanisms might be best placed in diverse contexts to deliver on the ultimate goals of halting extinctions and protecting biodiversity into the future. To do this it conducts impact evaluations of avoided deforestation comparing multiple governance mechanisms over the same temporal period, applying robust counter factual methods to account for covariates, and thus creates like for like impact estimates for these diverse mechanisms.

In addition to these new revisions from myself I summarize in the next section where, in my view, the authors responses to reviewer 2 have not been sufficient. I use the reviewer 2 exact requests and author responses to note what hasn't yet been addressed in full.

Reviewer 3 requests for further author revision to address Reviewer #2 comments:

Methods: Further clarification is required regarding treatment and control site selection.

While revisions have made some improvements to address reviewer 2 requests, main text methods remain unclear. At line 210 a concise statement at the very start of this methods section should articulate there are 6 possible interventions that are non overlapping spatially which they investigate. These are PA IUCN I-II, PA IUCN III-VI, IL, NTC, LC, MC. These should be defined in practical terms, logic - why they are relevant to study here – data selected, as well as theory of change in terms of why they would or would not be expected to result in changes to forest cover (as the outcome variable here) and longer term biodiversity.

Section on treatments line 209 - MC are entirely absent from the methods section starting line 209 so should be detailed

Controls –

The authors have omitted false controls and improved detail in the methods. However, the new text:

"We matched each treatment observation to a unique control observation to ensure each governance mechanism pixel was paired with only one control pixel that had not been matched previously. Matched pairs of pixels had to be within 0.2 SD of the propensity scores (Austin, 2011; Lunt, 2014)**. *Pixels could be reselected as control samples for each independent governance mechanism analysis."***

Is confusing. How can each governance mechanism pixel be paired with one control pixel that has **not** been matched previously but also allow for pixels to be **reselected as control samples for each independent governance mechanism.** The first sentence implies the pool of control pixels can only be used once, the second implies no replacement for individual matching experiments but allowing controls to be reselected across independent governance mechanisms analyses. Please clarify text.

Methods: As reviewer 2 noted, A control-intervention study design with matching is stronger than such a design without matching, but not as strong as before-after-control-intervention. Reviewer 2 requested

that the authors show trends in outcome variables for the control and intervention pools of pixels for each analysis and to test whether these trends are parallel. This is a simple and essential analysis to build credibility in the analyses. Yet the authors haven't implemented it. Rather they included trend as a covariate. I'm not convinced that is methodologically appropriate and it requires further discussion around why it is in fact a reasonable covariate. But this is separate to testing that trend lines pre intervention are the same across the two samples. The authors should implement the recommended test to show parallel trends in outcome variables prior to the study period.

4. Methods: lines 314+ - reviewer 2 noted that the methods raise issues of spatial autocorrelation and pseudo replication. The authors have revised and included methods to address spatial autocorrelation. However, the methods don't actually address pseudo replication (testing for Moran's I corrects for autocorrelation within a site but not that you may only have a small number of sites that are very large thus lending itself towards pseudo replication). The text line 314 "We explicitly accounted for spatial autocorrelation and pseudo-replication by estimating the residual spatial autocorrelation of these models (Moran's I) and then removing the pixels highly autocorrelated from the analysis." Should be revised to be "We explicitly accounted for spatial autocorrelation by estimating the residual spatial autocorrelation of these models (Moran's I) and then removing the pixels highly autocorrelated from the analysis.". The authors should report explicitly in a simple table the number, size and distribution of size of each intervention type. Some of this can be inferred in figure 5 but this isn't transparent enough. A reader should be able to rapidly refer to a table or numerical reporting of these details as they directly influence credibility of results given the likelihood of pseudo replication influencing some analyses and results.

11. Discussion. In response to reviewer requests the authors introduced this new paragraph for which I highlight text that I found to lack a level of precision needed to address complex and intertwined issues:

"At the global scale, our results show that while OECMs and PAs face considerable human pressure levels, they still have far lower human pressure than unprotected land. Previous work has shown that PAs face widespread levels of intense human pressure (Jones et al., 2018)***, but there is also evidence that they can reduce deforestation and habitat loss*** (Sze et al., 2022)***, and contain higher biodiversity than unprotected areas*** (Gray et al., 2016)***. Here, we showed that, on average, OECMs have similar human pressure levels to PAs, particularly those in IUCN categories III-VI. Less degraded ecosystems, such as those within many PAs and OECMS, support an exceptional confluence of globally significant environmental values relative to the ones that have experienced damaging human actions*** (Watson et al., 2018)***, so both OECMs and PAs have strong potential to deliver positive conservation outcomes. However, delivering on this potential will require concerted actions to overcome the many challenges facing area-based conservation efforts, such as underfinancing and under staffing*** (Coad et al., 2019; Lessmann et al., 2024)***, PADDD*** (Forrest et al., 2015; Golden Kroner et al., 2019)***, and resource extraction*** (Durán et al., 2013)***. Moreover, 42% of all reported OECMs were previously classified as PAs*** (Cook et al., 2024)***, so a better understanding of the causes, consequences and conservation impact of such changes is needed. Importantly, our global analysis does not consider the non-random location of PAs and OECMs and how this affects the effectiveness of these conservation mechanisms preventing biodiversity loss, which should be a priority for future research at the global scale."***

I make comment for each highlighted text item below:

they still have far lower human pressure than unprotected land. – include a reference please. You don't actually show this in your analysis as you only summarize HFI for PA and OECM not areas that are neither of these area based measures.

OECMs have similar human pressure levels to PAs, particularly those in IUCN categories III-VI. – You present a box plot with averages and scatter plot of points but you don't actually test for differences in distributions – you are making this conclusion based on a visual inspection presumably that the average for OECM is closest to PA III-VI and scatters are similar-ish? This analysis in general is highly descriptive without clear connection to your more rigorous impact evaluation. If you are including it needs to be elevated in rigour and clarified in purpose. How does a single time step summary of average human footprint relate to then a n impact evaluation for avoided deforestation across multiple mechanisms. I'd argue it does not. The measures of outcome are not quantitatively related (and correlation between HFI and deforestation does not make for a sound reasoning particularly when there are global deforestation layers that could be used instead if a global summary analysis was essential for this study). The mechanisms are not related other than tangentially in that the authors are seeking to make the case that some governance mechanisms tested in the Peruvian amazon case study may be appropriate for consideration as OECM and they then test for impacts across these and PAs. The geographic distribution is non overlapping -the global results don't overlap with Peru as Peru does not have OECM in the database as shown in the map. I would argue this global analysis detracts from the study and would suggest it should be removed. If the authors disagree they'd need to revise the text to create a far more logical rationale of research questions to link the analyse and improve their rigour.

Moreover, 42% of all reported OECMs were previously classified as PAs (Cook et al., 2024)***, so a better understanding of the causes, consequences and conservation impact of such changes is needed.*** – due to the preceding sentence emphasizing PADDD, this text reads as though the OECMS previously classified as PAs now reclassified as OECMs have experienced a PADDD (downgrade) event. Was this the intent by the authors? OECMs are not less than PAs – they are designed to be seen as equivalent not hierarchical in order or preference. This study should be developing evidence for which potential governance mechanisms might deliver equivalent outcomes. I'd suggest this sentence and context should be removed or revised to avoid this implication. A counter argument of a negative impact from reclassification is that there were be positive outcomes for people and nature - A change from PA to OECM now that countries can report OECMs could be seen as a good thing – we know have terminology that is inclusive and appropriate for describing particular governance mechanisms. Perhaps this is a PA upgrade?

Related to this point. Reviewer 2 comment on (previous) Lines 92-93. Note that Cook et al (OneEarth 7(3): 412-430) find that many protected areas around the world that have been reclassified as OECMs, now that there is a new accounting system that will allow governments to get credit for them. This observation should be considered and addressed with respect to the global analysis of human pressures within OECMs and PAs.

IN response to this the authors state:

We thank the reviewer for pointing this out. We have now included an additional paragraph in the introduction in lines 101 to 107 acknowledging this;

"More than 1.9 million km² of the Earth's surface has already been reported to the World Database on OECMs (UNEP-WCMC and IUCN, 2025)***, representing approximately 1% of total terrestrial area (Fig. 1). Two thirds of this area is governed by federal or national agencies*** (Jonas et al., 2024)***. With OECMs rapidly gaining attention and traction in conservation policy, a clear understanding of the***

effectiveness of various OECM designations as conservation mechanisms is needed (Jonas et al., 2024)***. It is also important to improve our understanding of the causes and consequences of PAs and OECMs downgrading, downsizing, and degazettement, given increasing reports of PAs being reclassified as OECMs*** (Cook et al., 2024)***."***

However as with the discussion text – this introduction text clearly insinuates that OECMs reclassified to PAs are equivalent to PADDD events. Please revise. The reviewer 2 comment was instead (in my view) asking the authors to recognize that they have conducted a single time step analysis of averaging HFI in area based measures attributing 'likeness' of OECMs and PA classes but without recognizing that actually 42% of OECMS were in fact in PA classifications previously. What does this reclassification thus mean for a conclusion drawn that OECMs are 'like' PAs in terms of their human footprint profiles. Simply put this issue goes to the root failure of the simple summary analysis. Again, I feel the global analysis is too simplistic to add value. If it remains the authors would need to recognize and confront some of the problems with this simplistic summary of the severe limitations in some of the inferences they attempt to make from it.
