# Supplementary material for: Potential of different area-based governance mechanisms for achieving Global Biodiversity Framework goals
Source: Nat Commun. 2026 Aug 3;17:9337. doi: 10.1038/s41467-026-76225-9 (PMC13529737; doi:10.1038/s41467-026-76225-9)
Supplement: Supplementary file 3 [file 41467_2026_76225_MOESM1_ESM.docx]

**Potential of different area-based governance mechanisms for achieving Global Biodiversity Framework goals**

Pablo Jose Negret*^1,2,3,4^, Victor J. Rincon-Parra^5^, Kendall R. Jones^6^, Vanessa R. Rathbone^6^, Sidney Novoa^7^, Marvin Quispe^7^, Armando Valdés-Velásquez^1,8^, German Forero-Medina^9^, Judith Schleicher^10^, Tatsuya Amano^11,12^, Miguel Saravia^1^, Julie Gwendolin Zaehringer^1,2,3^

^1^ Wyss Academy for Nature, Switzerland

^2^ Centre for Development and Environment, University of Bern, Switzerland

^3^ Institute of Geography, University of Bern, Switzerland

^4^ Hawkesbury Institute for the Environment, University of Western Sydney, Australia

^5^ Département de biologie, Université de Sherbrooke, Sherbrooke, Canada

^6^ Wildlife Conservation Society, New York, USA

^7^ Asociación para la Conservación de la Cuenca Amazónica, Lima, Perú

^8^ Grupo de Investigación en Sistemas Socio-Ecológicos, Universidad Peruana Cayetano Heredia, Peru.

^9^ Wildlife Conservation Society, Cali, Colombia

^10^ Deutsche Gesellschaft für Internationale Zusammenarbeit (GIZ) GmbH, Eschborn, Germany

^11^ School of The Environment, The University of Queensland, Queensland, Australia

^12^ Centre for Biodiversity and Conservation Science, The University of Queensland, Queensland, Australia

*[pablo.negret@wyssacademy.org](mailto:pablo.negret@wyssacademy.org)

**List of Supplementary Items**

**Supplementary Notes**

Supplementary Note 1: Human Pressure in OECMs and PAs globally	1

Supplementary Note 2: Moran’s I, multicollinearity test and stepwise selection procedure	14

**Supplementary Tables**

Supplementary Table 1. Total area (km²) and percentage of the study area (Peruvian Amazon) represented by each classification used to identify or exclude potential treatment and control pixels.	2

Supplementary Table 2. Main residual governance mechanisms (relevant at scale) that could occur within the control pool, along with their general characteristics.	3

Supplementary Table 3. Variance inflation factor by variable for the matching analysis for Protected Areas (IUCN I–II).	4

Supplementary Table 4. Variance inflation factor by variable for the matching analysis for Protected Areas (IUCN III–VI).	4

Supplementary Table 5. Variance inflation factor by variable for the matching analysis for Non-timber forest product concessions.	5

Supplementary Table 6. Variance inflation factor by variable for the matching analysis for Indigenous Lands.	6

Supplementary Table 7. Variance inflation factor by variable for the matching analysis for Logging Concessions.	7

Supplementary Table 8. Variance inflation factor by variable for the matching analysis for Mining Concessions.	8

Supplementary Table 9. Results of Wilcoxon Rank Sum Test of differences in mean HFP values across OECMs and PAs of varying IUCN categories.	9

Supplementary Table 10. Land area with varying human pressure levels in 2020, across OECMs, PAs and unprotected land.	9

Supplementary Table 11. Outputs from independent binomial logistic regression models assessing the effect of each governance mechanism on pixel-level deforestation probability.	9

Supplementary Table 12. Difference between estimation of a governance mechanism’s effect on forest loss based on weighting by sites or by pixels.	10

**Supplementary Figures**

Supplementary Figure 1. Linear regression plot showing the relationship between the Venter et al. and Sanderson et al. human pressure data.	11

Supplementary Figure 2. Estimated proportional forest loss inside Protected Areas, Indigenous Land, Non-Timber Forest Products, Logging and Mining Concessions in the Peruvian Amazon (2005–2021) against matched areas.	12

Supplementary Figure 3. Flow diagram of the process for the selection of control and treatment pixels for the matching analysis for each governance mechanism.	13

Supplementary Figure 4. Multicollinearity assessment of covariates for the matching analysis for Protected Areas (IUCN I–II).	14

Supplementary Figure 5. Multicollinearity assessment of covariates for the matching analysis for Protected Areas (IUCN III–VI).	14

Supplementary Figure 6. Multicollinearity assessment of covariates for the matching analysis for Indigenous Lands.	15

Supplementary Figure 7. Multicollinearity assessment of covariates for the matching analysis for Non-timber forest product concessions.	15

Supplementary Figure 8. Multicollinearity assessment of covariates for the matching analysis for Logging Concessions.	16

Supplementary Figure 9. Multicollinearity assessment of covariates for the matching analysis for Mining Concessions.	16

Supplementary Figure 10. Stepwise selection process for the matching analysis for Protected Areas (IUCN I–II).	17

Supplementary Figure 11. Stepwise selection process for the matching analysis for Protected Areas (IUCN III–VI).	17

Supplementary Figure 12. Stepwise selection process for the matching analysis for Indigenous Land.	18

Supplementary Figure 13. Stepwise selection process for the matching analysis for Non-timber forest product concessions.	18

Supplementary Figure 14. Stepwise selection process for the matching analysis for Logging Concessions.	19

Supplementary Figure 15. Stepwise selection process for the matching analysis for Logging Concessions (alternative model).	19

Supplementary Figure 16. Stepwise selection process for the matching analysis for Mining Concessions.	20

Supplementary Figure 17. Global Moran’s I of forest loss for Protected Areas (IUCN I–II) across distance classes before and after adjustment.	21

Supplementary Figure 18. Global Moran’s I of forest loss for Protected Areas (IUCN III–VI) across distance classes before and after adjustment.	21

Supplementary Figure 19. Global Moran’s I of forest loss for Indigenous Lands across distance classes before and after adjustment.	22

Supplementary Figure 20. Global Moran’s I of forest loss for Non-timber forest product concessions across distance classes before and after adjustment.	22

Supplementary Figure 21. Global Moran’s I of forest loss for Logging Concessions across distance classes before and after adjustment.	23

Supplementary Figure 22. Global Moran’s I of forest loss for Mining Concessions across distance classes before and after adjustment.	23

Supplementary Figure 23. Indices of covariate imbalance before and after the matching runs for each governance mechanism.	24

Supplementary Figure 24. Distribution of propensity scores before and after matching for Protected Areas (IUCN I–II).	25

Supplementary Figure 25. Distribution of propensity scores before and after matching for Protected Areas (IUCN III–VI).	25

Supplementary Figure 26. Distribution of propensity scores before and after matching for Non-timber forest product concessions.	26

Supplementary Figure 27. Distribution of propensity scores before and after matching for Indigenous Land.	26

Supplementary Figure 28. Distribution of propensity scores before and after matching for Logging Concessions.	27

Supplementary Figure 29. Distribution of propensity scores before and after matching for Mining Concessions.	27

Supplementary Figure 30. Cumulative deforestation (%) from 2000 to 2021 for treatment and matched control pixels across governance mechanisms.	28

Supplementary Figure 31. Empirical cumulative distribution functions (ECDFs) of pre-intervention deforestation (2000–2005) within a 1 km² radius, before and after matching.	29

Supplementary Figure 32. Empirical cumulative distribution functions (ECDFs) of pre-intervention deforestation (2000–2005) within a 10 km² radius, before and after matching.	30

Supplementary Figure 33. Linear regression plot showing the relationship between OECM area and mean human footprint scores.	31

Supplementary Figure 34. Proportion of land under high pressure (>6.69 HFP) within protected area boundaries for countries that have submitted OECM data to WDPA.	32

Supplementary Figure 35. Estimated proportional forest loss inside Protected Areas (PAs), Indigenous Land (ILs), Non-Timber Forest Products (NTCs), Logging (LCs) and Mining Concessions (MCs) in the Peruvian Amazon (2005–2021).	33

Supplementary Figure 36. Histograms of the number of sites per size range, by governance mechanism and combined.	34

Supplementary Figure 37. Histograms of the number of pixels per site per size range, by governance mechanism and combined.	34

**Supplementary information:**

***Supplementary note 1:

Human Pressure in OECMs and PAs globally***

Regression between Human Footprint (HFP) data from Venter et al. ^1^ and Sanderson et al. ^2^

We used HFP data from Sanderson et al. ^2^ to assess the level of human pressure within OECMs and PAs globally. Because previous papers examining different categories of human pressure and their relationship with biodiversity loss were based on Venter et al. ^1^, we ran a spatial regression between the Sanderson and Venter HFP datasets to understand how well correlated they are with one another. Venter et al. ^1^ ranges from 0-50 and Sanderson et al. ^2^ ranges from 0-64. The linear model showed that Venter’s HFP is a significant predictor of Sanderson’s HFP Index with a p-value of < 0.2.2e-16 and an R^2^ value of 0.7966. Supplementary Figure 2 demonstrates the correlation of the two datasets.

We then used the regression equation from this model to determine values from Sanderson et al. ^2^ that correspond with previously used values from Venter et al. ^1^. This analysis showed that values from Venter et al. ^1^ of 3 and 7 correspond with values of 3.13 and 6.69 in Sanderson et al. ^2^, respectively. As such, we used these values to develop three categories of human pressure: low pressure (HFP 0-3.13), medium pressure (3.13-6.69), and high pressure (>6.69)

**Supplementary Table 1.** total area (km2) and percentage of the study area (i.e., Peruvian Amazon) represented by each of the classifications used to identify or exclude potential treatment and control pixels for the analysis.

|  | **PA I - II** | | | **PA III-IV** | | | **NTFC** | | | **IL** | | |
| --- | --- | --- | --- | --- | --- | --- | --- | --- | --- | --- | --- | --- |
|  | ***Area*** | ***Percentage (%)*** | ***Pixels*** | ***Area*** | ***Percentage (%)*** | ***Pixels*** | ***Area*** | ***Percentage (%)*** | ***Pixels*** | ***Area*** | ***Percentage (%)*** | ***Pixels*** |
| **Total initial area 2005** | 75502 | 9.7 | 84114076 | 51565 | 6.6 | 57319622 | 9552 | 1.2 | 10694099 | 151216 | 19.4 | 168110352 |
| **Sites stablished after 2005** | 25140 | 3.2 | 27962164 | 9784 | 1.3 | 10870178 | 7425 | 1.0 | 8260825 | 27099 | 3.5 | 30110880 |
| **Overlap** | 261 | 0.0 | 290645 | 746 | 0.1 | 828861 | 574 | 0.1 | 641491 | 3788 | 0.5 | 4218070 |
| **Buffer zones** | 31102 | 4.0 | 34620579 | 34244 | 4.4 | 38080544 | 7702 | 1.0 | 8597075 | 47074 | 6.0 | 52329750 |
| **Total area used for analysis** | 75241 | 9.6 | 83823431 | 50818 | 6.5 | 56490761 | 9279 | 1.2 | 10052608 | 147428 | 18.9 | 163892282 |
| **Forest 2005** | 73006 | 9.3 | 81041417 | 48312 | 6.2 | 53582207 | 9412 | 1.2 | 9823984 | 140671 | 18.0 | 152544248 |
| **Sampling pixels** |  |  | 176073 |  |  | 118710 |  |  | 22474 |  |  | 342461 |
| **Matched pixels** |  |  | 64431 |  |  | 46931 |  |  | 5214 |  |  | 256377 |

|  | **LC** | | | **MC** | | | **Control** | | |
| --- | --- | --- | --- | --- | --- | --- | --- | --- | --- |
|  | ***Area*** | ***Percentage (%)*** | ***Pixels*** | ***Area*** | ***Percentage (%)*** | ***Pixels*** | ***Area*** | ***Percentage (%)*** | ***Pixels*** |
| **Total initial area 2005** | 57919 | 7.4 | 64450821 | 2968 | 0.4 | 3314339 | 434805 | 55.6 | 480155171 |
| **Sites stablished after 2005** | 11291 | 1.4 | 12544822 | 11039 | 1.4 | 12304784 | 91480 | 11.7 | 96398502 |
| **Overlap** | 2080 | 0.3 | 2315491 | 905 | 0.1 | 1011275 | 6216 | 0.8 | 6899299 |
| **Buffer zones** | 18803 | 2.4 | 20906441 | 17616 | 2.3 | 19635329 | 107435 | 13.7 | 118768130 |
| **Total area used for analysis** | 58839 | 7.5 | 62135330 | 2639 | 0.3 | 2802878 | 269007 | 34.4 | 295704788 |
| **Forest 2005** | 57294 | 7.3 | 61145715 | 2049 | 0.3 | 2182400 | 227977 | 29.2 | 251419971 |
| **Sampling pixels** |  |  | 131356 |  |  | 4319 |  |  | 689604 |
| **Matched pixels** |  |  | 13745 |  |  | 1718 |  |  |  |

**Supplementary Table 2.** Main residual governance mechanisms (relevant at scale) that could occur within the control pool, along with their general characteristics.

| **Type of mechanism** | **Governance mechanism (English/Spanish)** | **Characteristics** | **Potential influence on forest cover** | **Information source** | **Link** |
| --- | --- | --- | --- | --- | --- |
| Sub-national conservation areas | Regional Conservation Areas / Áreas de Conservación Regional (ACR) | Conservation areas created and managed by regional governments; legally recognized and part of the national conservation system, but not national protected areas | Often associated with lower deforestation relative to unallocated land due to management and restrictions, though effectiveness varies by region | MINAM / Peruvian national open data platform | <https://www.datosabiertos.gob.pe/dataset/%C3%A1reas-de-conservacion-regional-acr> |
| Private conservation | Private Conservation Areas / Áreas de Conservación Privada (ACP) | Voluntary conservation areas on private or communal lands, formally recognized by the state | Typically lower deforestation due to conservation commitments and landholder incentives | MINAM / Peruvian national open data platform | <https://www.datosabiertos.gob.pe/dataset/%C3%A1reas-de-conservaci%C3%B3n-privada-acp> |
| State forest zoning | Permanent Production Forests without active concession / Bosques de Producción Permanente (BPP) sin concesión activa | Forest areas designated by the state for permanent forest production, regardless of whether a concession is currently allocated | Mixed effects; may reduce conversion in some areas but also represent land intended for future extractive use | SERFOR | [https://faolex.fao.org/docs/pdf/per183073anx.pdf https://geo.serfor.gob.pe/visor/](https://faolex.fao.org/docs/pdf/per183073anx.pdf) |
| Private rural land | Rural and private properties | These are private lands primarily used for subsistence and commercial farming. According to Peruvian law, they must maintain at least 30% of their forest coverage. | These sites range from 5 to 50 hectares in size, where deforestation is occurring primarily for agricultural expansion | MIDAGRI | <https://www.leyes.congreso.gob.pe/Documentos/DecretosLegislativos/00667.pdf> |
| Unallocated public forest | Unassigned forests or public domain lands / Bosques sin asignación o tierras de dominio público | Forest lands not assigned to any formal tenure or conservation category | Generally higher deforestation risk due to open access and future allocation pressures | Defined in Methods (this study) |  |

**Supplementary Table 3.** Variance inflation Factor by variable to define the cofounding variables for the matching analysis for Protected Areas in categories I and II.

| Var | group | VIF |
| --- | --- | --- |
| Anual_Prec | 1 | 4,19617913 |
| D17Set10 | 2 | 3,992664658 |
| D7Set1000 | 3 | 49,50184711 |
| D17Set1000 | 3 | 50,64197042 |
| D17Set10000 | 4 | 44,50020714 |
| D17Set5000 | 5 | 18,61537382 |
| D7Set5000 | 5 | 53,46230843 |
| D7Set10 | 6 | 6,183456052 |
| D7Set10000 | 7 | 14,34661468 |
| defor_2000_2005_10km2 | 8 | 1,769025892 |
| defor_2000_2005_1km2 | 9 | 1,278853123 |
| Departme_R | 10 | 6,921417004 |
| District_R | 10 | 14,56995062 |
| Department | 11 | 2,925805439 |
| Dis_Def | 12 | 1,43442186 |
| Dis_Rivers | 13 | 1,228318659 |
| Ecoregions | 14 | 3,797183164 |
| Elevation | 15 | 4,665480563 |
| National_R | 16 | 2,717306275 |
| Pop2000 | 17 | 1,93335571 |
| Pop2020 | 18 | 1,218792489 |
| Prec_Seas | 19 | 5,453210732 |
| Slope | 20 | 1,924347043 |
| Tra_Time00 | 21 | 3,894815681 |
| Tra_Time15 | 21 | 5,299528803 |

**Supplementary Table 4.** Variance inflation Factor by variable to define the cofounding variables for the matching analysis for Protected Areas in categories III and VI.

| Var | group | VIF |
| --- | --- | --- |
| Anual_Prec | 1 | 4,009870011 |
| D17Set10 | 2 | 5,033076797 |
| D7Set10 | 2 | 5,423887539 |
| D7Set1000 | 3 | 59,61210114 |
| D17Set1000 | 3 | 60,80335258 |
| D7Set10000 | 4 | 28,81414527 |
| D17Set10000 | 4 | 35,38162879 |
| D17Set5000 | 5 | 18,88822751 |
| D7Set5000 | 5 | 33,62678065 |
| defor_2000_2005_10km2 | 6 | 1,797042207 |
| defor_2000_2005_1km2 | 7 | 1,348187832 |
| Departme_R | 8 | 2,599898954 |
| Department | 9 | 5,758819268 |
| Dis_Def | 10 | 1,284782032 |
| Dis_Rivers | 11 | 1,240691944 |
| District_R | 12 | 7,147546228 |
| Ecoregions | 13 | 3,628094228 |
| Elevation | 14 | 4,658799073 |
| National_R | 15 | 2,756406586 |
| Pop2020 | 16 | 2,014600238 |
| Pop2000 | 16 | 2,136423731 |
| Prec_Seas | 17 | 7,679894898 |
| Slope | 18 | 2,523011239 |
| Tra_Time00 | 19 | 3,329298039 |
| Tra_Time15 | 20 | 4,398947848 |

**Supplementary Table 5.** Variance inflation Factor by variable to define the cofounding variables for the matching analysis for Non-timber forest product concessions.

| Var | group | VIF |
| --- | --- | --- |
| Anual_Prec | 1 | 6,647045015 |
| D17Set5000 | 1 | 8,110712554 |
| D17Set10 | 2 | 3,439722962 |
| D17Set1000 | 3 | 8,801536772 |
| D7Set10000 | 4 | 86,22243288 |
| D17Set10000 | 4 | 126,0695147 |
| D7Set10 | 5 | 7,049294855 |
| D7Set1000 | 6 | 3,752748673 |
| Departme_R | 7 | 13,8342944 |
| D7Set5000 | 7 | 33,10562607 |
| defor_2000_2005_10km2 | 8 | 3,074101042 |
| defor_2000_2005_1km2 | 9 | 1,730693598 |
| Department | 10 | 5,049811172 |
| Dis_Def | 11 | 1,689394066 |
| Dis_Rivers | 12 | 1,946321225 |
| District_R | 13 | 20,72781625 |
| Ecoregions | 14 | 1,852947117 |
| Elevation | 15 | 4,2102101 |
| National_R | 16 | 6,736317083 |
| Prec_Seas | 16 | 15,16676828 |
| Pop2000 | 17 | 3,698942279 |
| Pop2020 | 18 | 2,989904395 |
| Slope | 19 | 1,740763791 |
| Tra_Time00 | 20 | 5,638285932 |
| Tra_Time15 | 21 | 9,698304156 |

**Supplementary Table 6.** Variance inflation Factor by variable to define the cofounding variables for the matching analysis for Indigenous Lands.

| Var | group | VIF |
| --- | --- | --- |
| Anual_Prec | 1 | 4,009870011 |
| D17Set10 | 2 | 5,033076797 |
| D7Set10 | 2 | 5,423887539 |
| D7Set1000 | 3 | 59,61210114 |
| D17Set1000 | 3 | 60,80335258 |
| D7Set10000 | 4 | 28,81414527 |
| D17Set10000 | 4 | 35,38162879 |
| D17Set5000 | 5 | 18,88822751 |
| D7Set5000 | 5 | 33,62678065 |
| defor_2000_2005_10km2 | 6 | 1,797042207 |
| defor_2000_2005_1km2 | 7 | 1,348187832 |
| Departme_R | 8 | 2,599898954 |
| Department | 9 | 5,758819268 |
| Dis_Def | 10 | 1,284782032 |
| Dis_Rivers | 11 | 1,240691944 |
| District_R | 12 | 7,147546228 |
| Ecoregions | 13 | 3,628094228 |
| Elevation | 14 | 4,658799073 |
| National_R | 15 | 2,756406586 |
| Pop2020 | 16 | 2,014600238 |
| Pop2000 | 16 | 2,136423731 |
| Prec_Seas | 17 | 7,679894898 |
| Slope | 18 | 2,523011239 |
| Tra_Time00 | 19 | 3,329298039 |
| Tra_Time15 | 20 | 4,398947848 |

**Supplementary Table 7.** Variance inflation Factor by variable to define the cofounding variables for the matching analysis for Logging Concessions.

| Var | group | VIF |
| --- | --- | --- |
| Anual_Prec | 1 | 2,875102843 |
| D17Set10 | 2 | 3,114080471 |
| D7Set1000 | 3 | 36,03667605 |
| D17Set1000 | 3 | 37,09127967 |
| D7Set10000 | 4 | 12,62166669 |
| D17Set10000 | 4 | 18,29664534 |
| D17Set5000 | 5 | 17,20840376 |
| D7Set5000 | 5 | 24,23874893 |
| D7Set10 | 6 | 3,997579495 |
| defor_2000_2005_10km2 | 7 | 2,344897066 |
| defor_2000_2005_1km2 | 8 | 1,84413297 |
| Departme_R | 9 | 7,22183499 |
| District_R | 9 | 10,20597744 |
| Department | 10 | 4,889208827 |
| Dis_Def | 11 | 1,529117887 |
| Dis_Rivers | 12 | 1,330730248 |
| Ecoregions | 13 | 3,655053355 |
| Elevation | 14 | 4,091463316 |
| National_R | 15 | 2,91134303 |
| Pop2000 | 16 | 2,477589886 |
| Pop2020 | 17 | 2,113424291 |
| Prec_Seas | 18 | 7,060753129 |
| Slope | 19 | 2,302216865 |
| Tra_Time00 | 20 | 3,783402268 |
| Tra_Time15 | 21 | 6,597281578 |

**Supplementary Table 8.** Variance inflation Factor by variable to define the cofounding variables for the matching analysis for Mining Concessions.

| Var | group | VIF |
| --- | --- | --- |
| D17Set5000 | 1 | 4,019131323 |
| Anual_Prec | 1 | 7,166156435 |
| D17Set10 | 2 | 2,651653791 |
| D7Set1000 | 3 | 3,68213257 |
| D17Set1000 | 3 | 3,823929095 |
| D7Set10000 | 4 | 1136,097656 |
| D17Set10000 | 4 | 1212,072195 |
| D7Set10 | 5 | 2,997520001 |
| Department | 6 | 3,05981482 |
| D7Set5000 | 6 | 66,20762863 |
| defor_2000_2005_10km2 | 7 | 2,251361219 |
| defor_2000_2005_1km2 | 8 | 1,630356254 |
| Departme_R | 9 | 2,295305525 |
| Dis_Def | 10 | 1,243984502 |
| Dis_Rivers | 11 | 1,239424618 |
| District_R | 12 | 4,879385649 |
| Ecoregions | 13 | 2,22211039 |
| Elevation | 14 | 5,071807522 |
| National_R | 15 | 3,621669554 |
| Pop2020 | 16 | 1,779011641 |
| Pop2000 | 16 | 2,046144898 |
| Prec_Seas | 17 | 3,540129042 |
| Slope | 18 | 1,857786601 |
| Tra_Time00 | 19 | 2,260423798 |
| Tra_Time15 | 20 | 3,411173823 |

**Supplementary Table 9.** Results of Wilcoxon Rank Sum Test of differences in mean HFP values across OECMS and PAs of varying IUCN categories.

|  | **OECM** | **PA: IUCN I-II** | **PA: IUCN III-VI** |
| --- | --- | --- | --- |
| **PA: IUCN I-II** | < 0.05 | - | - |
| **PA: IUCN III-VI** | < 0.05 | < 0.05 | - |
| **PA: NA** | < 0.05 | < 0.05 | < 0.05 |

**Supplementary Table 10.** Land area with varying human pressure levels in year 2020, across OECMs, PAs and unprotected land. The large area under low human pressure in OECMS is primarily driven by a small number of very large OECMS with low pressure levels.

|  | **OECMs** | | **PAs: IUCN I-II** | | **PAs: IUCN III-VI** | | **PAs: NA** | | **Unprotected Land** | |  |
| --- | --- | --- | --- | --- | --- | --- | --- | --- | --- | --- | --- |
|  |  |  |  |  |  |  |  |  |  |  |  |
| **HII Category** | ***Area (km2)*** | ***Percentage (%)*** | ***Area (km2)*** | ***Percentage (%)*** | ***Area (km2)*** | ***Percentage (%)*** | ***Area (km2)*** | ***Percentage (%)*** | ***Area (km2)*** | ***Percentage (%)*** |  |
|  |  |  |  |  |  |  |  |  |  |  |  |
| ***High Pressure (6.69+)*** | 264’044 | 17.04 | 59’847 | 4.4 | 130’846 | 35.99 | 84’352 | 22.64 | 3’836’016 | 22.3 |  |
| ***Medium Pressure (3.13 – 6.69)*** | 190’844 | 12.32 | 154’853 | 11.38 | 65’624 | 18.05 | 58’151 | 15.6 | 2’261’169 | 13.14 |  |
| ***Low Pressure (0-3.13)*** | 1’094’417 | 70.64 | 1’145’523 | 84.22 | 167’073 | 45.96 | 230’156 | 61.76 | 11’107’392 | 64.5 |  |

**Supplementary Table 11.** Outputs from independent binomial logistic regression models assessing the effect of each governance mechanism on pixel-level deforestation probability by comparing treated and matched control pixels (deforestation ~ treatment). Reported p-values correspond to two-sided tests of the treatment coefficient; no adjustments for multiple comparisons were applied.

| **Governance** | **estimate** | **std error** | **z statistic** | **p value** | **conf low** | **conf high** | **P value round** | **sign** |
| --- | --- | --- | --- | --- | --- | --- | --- | --- |
| ***NTC*** | -0.3136688 | 0.08391802 | -3.7378004 | 0.00018564 | -0.4781451 | -0.1491925 | 2e-04 | *** |
| ***IL*** | -0.244229 | 0.01375425 | -17.756614 | 1.532E-70 | -0.2711868 | -0.2172711 | 2e-70 | *** |
| ***LC*** | 0.1487647 | 0.05396779 | 2.75654591 | 0.00584154 | 0.04298977 | 0.25453964 | 0.006 | ** |
| ***MC*** | 0.36280041 | 0.08128299 | 4.46342343 | 8.066E-06 | 0.20348867 | 0.52211214 | 8e-06 | *** |
| ***PA_I.II*** | -0.917672 | 0.06276296 | -14.621234 | 2.0564E-48 | -1.0406851 | -0.7946588 | 2e-48 | *** |
| ***PA_III.VI*** | -0.688828 | 0.06510523 | -10.580225 | 3.6807E-26 | -0.8164319 | -0.5612241 | 4e-26 | *** |

**Supplementary Table 12.** Difference between estimation of a governance mechanisms effect on forest loss based on weighing by sites or by pixels.

| **Governance** | **Avoid deforestation** | | | | | |
| --- | --- | --- | --- | --- | --- | --- |
|  | **Pixels** | | **Sites** | | **Higher** | |
|  | ***Inside*** | ***Outside*** | ***Inside*** | ***Outside*** | ***Pixels*** | ***Sites*** |
| ***Non-Timber Forest Products Concessions*** | 1.73 | -1.73 | 1.60 | -1.60 | Inside | Inside |
| ***Indigenous Land*** | 1.02 | -1.02 | -4.70 | 4.70 | Inside | Outside |
| ***Logging Concessions*** | -0.75 | 0.75 | -1.72 | 1.72 | Outside | Outside |
| ***Mining Concessions*** | -6.46 | 6.46 | -13.09 | 13.09 | Outside | Outside |
| ***Protected Areas I&II*** | 0.83 | -0.83 | 1.07 | -1.07 | Inside | Inside |
| ***Protected Areas III–VI*** | 0.75 | -0.75 | 0.97 | -0.97 | Inside | Inside |

**Supplementary Figure 1.** Linear regression plot showing the relationship between the Venter et al. ^1^ and Sanderson et al. ^2^ human pressure data.

**Supplementary Figure 2.** The estimated proportional forest loss inside Protected Areas, Indigenous land, Non-Timber Forest Products, Logging and Mining Concessions in the Peruvian Amazon from 2005 to 2021 against analogous matched areas. black lines, 95% CI; Percentage values on top of each governance mechanism reflect the decrease (green) or increase (red) in deforestation proportional to the matched control pixels. The model-estimate axis depicts the effect of the governance mechanism on forest loss based on a logistic regression (see methods), *p < 0.05, **p < 0.01, ***p < 0.001. Including all the sites **(a)** and excluding sites that stopped operating between 2005 and 2021 **(c).** Estimated proportional carbon emissions inside Protected Areas, Indigenous land, Non-Timber Forest Products, Logging and Mining Concessions in the Peruvian Amazon from 2005 to 2021 against analogous matched areas. black lines, 95% CI; Percentage values on top of each governance mechanism reflect the decrease (green) or increase (red) in deforestation or carbon emissions proportional to the matched control pixels. Including all the sites **(c)** and excluding sites that stopped operating between 2005 and 2021 **(d).** In parentheses is the sample size corresponding to the number of pixels in the treatment as well as the control used for the matching analysis for each governance mechanism assessed.

**Supplementary Figure 3.** Flow diagram of the process for the selection of the pool of control and treatment pixels for the matching analysis for each governance mechanisms assess in the Peruvian Amazon. Base map derived from Natural Earth data (public domain).

**Supplementary note 2.** Moran’s I, multicollinearity test and stepwise selection procedure to define the cofounding variables for the matching analysis to assess the impact of Protected Areas, Indigenous Land, Non-Timber Forest Products, Logging and Mining Concessions on forest loss between 2005 and 2021.

**Supplementary Figure 4.** Multicollinearity assessment of covariates used in the matching analysis for Protected Areas (IUCN I–II). The dashed line indicates the correlation threshold used to exclude highly correlated variables.

**Supplementary Figure 5.** Multicollinearity assessment of covariates used in the matching analysis for Protected Areas (IUCN III–VI). The dashed line indicates the correlation threshold used to exclude highly correlated variables.

**Supplementary Figure 6.** Multicollinearity assessment of covariates used in the matching analysis for Indigenous Lands. The dashed line indicates the correlation threshold used to exclude highly correlated variables.

**Supplementary Figure 7.** Multicollinearity assessment of covariates used in the matching analysis for Non-timber forest product concessions. The dashed line indicates the correlation threshold used to exclude highly correlated variables.

**Supplementary Figure 8.** Multicollinearity assessment of covariates used in the matching analysis for Logging Concessions. The dashed line indicates the correlation threshold used to exclude highly correlated variables.

**Supplementary Figure 9.** Multicollinearity assessment of covariates used in the matching analysis for Mining Concessions. The dashed line indicates the correlation threshold used to exclude highly correlated variables.

**Supplementary Figure 10.** Stepwise selection process to define the cofounding variables for the matching analysis for Protected Areas in categories I and II.

**Supplementary Figure 11.** Stepwise selection process to define the cofounding variables for the matching analysis for Protected Areas in categories III and VI.

**Supplementary Figure 12.** Stepwise selection process to define the cofounding variables for the matching analysis for Indigenous Land.

**Supplementary Figure 13.** Stepwise selection process to define the cofounding variables for the matching analysis for Non-timber forest product concessions.

**Supplementary Figure 14.** Stepwise selection process to define the cofounding variables for the matching analysis for Logging Concessions.

**Supplementary Figure 15.** Stepwise selection process to define the cofounding variables for the matching analysis for Logging Concessions.

**Supplementary Figure 16.** Stepwise selection process to define the cofounding variables for the matching analysis for Mining Concessions.

**Supplementary Figure 17.** Global Moran’s I of forest loss for Protected Areas (IUCN I–II) across distance classes before (initial) and after adjustment. Points indicate Moran’s I values; colours represent iteration and significance of spatial autocorrelation.

**Supplementary Figure 18.** Global Moran’s I of forest loss for Protected Areas (IUCN III–VI) across distance classes before (initial) and after adjustment. Points indicate Moran’s I values; colours represent iteration and significance of spatial autocorrelation.

**Supplementary Figure 19.** Global Moran’s I of forest loss for Indigenous Lands across distance classes before (initial) and after adjustment. Points indicate Moran’s I values; colours represent iteration and significance of spatial autocorrelation.

**Supplementary Figure 20.** Global Moran’s I of forest loss for Non-timber forest product concessions across distance classes before (initial) and after adjustment. Points indicate Moran’s I values; colours represent iteration and significance of spatial autocorrelation.

**Supplementary Figure 21.** Global Moran’s I of forest loss for Logging Concessions across distance classes before (initial) and after adjustment. Points indicate Moran’s I values; colours represent iteration and significance of spatial autocorrelation.

**Supplementary Figure 22.** Global Moran’s I of forest loss for Mining Concessions across distance classes before (initial) and after adjustment. Points indicate Moran’s I values; colours represent iteration and significance of spatial autocorrelation.

**Supplementary Figure 23.** Indices of covariate imbalance before and after the matching runs for each governance mechanism, to assess the robustness of the matching analysis reducing bias in the control units.

**Supplementary Figure 24.** Distribution of propensity scores before and after matching for Protected Areas (IUCN I–II). Bars show treated and control units.

**Supplementary Figure 25.** Distribution of propensity scores before and after matching for Protected Areas (IUCN III–VI).

**Supplementary Figure 26.** Distribution of propensity scores before and after matching for Non-timber forest product concessions.

**Supplementary Figure 27.** Distribution of propensity scores before and after matching for Indigenous Land.

**Supplementary Figure 28.** Distribution of propensity scores before and after matching for Logging Concessions.

**Supplementary Figure 29.** Distribution of propensity scores before and after matching for Mining Concessions.

**Supplementary Figure 30.** Cumulative deforestation (%) from 2000 to 2021 for treatment and matched control pixels across governance mechanisms. The shaded grey area indicates the pre-intervention period (2000–2005). Lines represent mean cumulative deforestation for treatment (yellow) and control (green) pixels, with shaded bands showing confidence intervals.

**Supplementary Figure 31.** Empirical cumulative distribution functions (ECDFs) of pre-intervention deforestation (2000–2005) measured within a 1 km² radius around each pixel, shown for treatment and control pixels before and after matching. Greater overlap between curves in the post-matching panels indicates improved similarity in landscape-scale pre-intervention deforestation trends.

**Supplementary Figure 32.** Empirical cumulative distribution functions (ECDFs) of pre-intervention deforestation (2000–2005) measured within a 10 km² radius around each pixel, shown for treatment and control pixels before and after matching. Greater overlap between curves in the post-matching panels indicates improved balance in landscape-scale pre-intervention deforestation trends.

**Supplementary Figure 33.** Linear regression plot showing the relationship between OECM area and mean human footprint scores. A log scale was used for OECM area as there is an small number of extremely large OECMS.

**Supplementary Figure 34.** Proportion of land under high pressure (>6.69 HFP) within protected area boundaries for countries that have submitted OECM data to WDPA. Inset maps show the proportion of land under high pressure (>6.69 HFP) within protected area boundaries for parts of **(b)** Canada, **(c)** Sweden, **(d)** Bhutan, **(e)** Colombia, **(f)** Morocco and Algeria, **(g)** Japan. Base map derived from Natural Earth data (public domain).

**Supplementary Figure 35.** The estimated proportional forest loss inside Protected Areas (PAs), Indigenous land (ILs), Non-Timber Forest Products (NTCs), Logging (LCs) and Mining Concessions (MCs) in the Peruvian Amazon from 2005 to 2021 against analogous matched areas. black lines, 95% CI; Percentage values on top of each governance mechanism reflect the decrease (green) or increase (red) in deforestation proportional to the matched control pixels. Results of the analysis done at the pixel level **(a)** and at the site level **(c).** Estimated proportional carbon emissions inside Protected Areas (PAs), Indigenous land (ILs), Non-Timber Forest Products (NTCs), Logging (LCs) and Mining Concessions (MCs) in the Peruvian Amazon from 2005 to 2021 against analogous matched areas. black lines, 95% CI; Percentage values on top of each governance mechanism reflect the decrease (green) or increase (red) in carbon emissions proportional to the matched control pixels. Results of the analysis done at the pixel level **(b)** and at the site level **(d).** In parentheses is the sample size corresponding to the number of pixels or sites in the treatment as well as the control used for the analysis for each governance mechanism assessed.

**Supplementary Figure 36.** Histograms of the number of sites per size range. Individually by governance mechanism **(a)** and together **(b)**. In parentheses is the sample size corresponding to the number of sites per governance mechanisms.

**Supplementary Figure 37.** Histograms of the number of pixels per sites on a particular size range. Individually by governance mechanism **(a)** and together **(b)**. In parentheses is the sample size corresponding to the number of sites per governance mechanisms.

**References**

1.	Venter, O. *et al.* Sixteen years of change in the global terrestrial human footprint and implications for biodiversity conservation. *Nature Communications* **7**, (2016).

2.	Sanderson, E. *et al.* The March of the Human Footprint. Preprint at https://doi.org/10.32942/OSF.IO/D7RH6 (2022).
